# Supplementary material for: Precision and accuracy of FEV1 measurements from the Vitalograph copd-6 mini-spirometer in a healthy Ugandan population
Source: PLoS One. 2021 Jun 28;16(6):e0253319. doi: 10.1371/journal.pone.0253319 (PMC8238209; doi:10.1371/journal.pone.0253319)
Supplement: S1 File — (PDF) [file pone.0253319.s001.pdf]

# S1 File: Deviations between analysis protocol and final analyses

## 1 Quality criteria

The analysis protocol [1] stated that to be included in our analyses, a participant had to have performed three good blows with the copd-6 (i.e., none of the three blows performed could have triggered warnings), and that we would use standard ATS quality criteria [2] for the MicroDL examinations. However, these strict quality criteria would lead us to discard > 50% of all spirometric examinations, potentially introducing selection bias into our analyses. We therefore decided to use looser quality criteria as listed below:

copd-6

- At least two blows without warnings
- We only used numerical results from blows without warnings
- Repeatability: Difference between best and second-best $FEV_1$ and $FEV_6$ < 0.25 liters.

MicroDL

- At least two acceptable blows according to ATS criteria [2]
- We only used numerical results from blows that were “usable” according to ATS criteria [2]
- Repeatability: Difference between best and second-best $FEV_1$ and FVC < 0.25 liters.

A post-hoc sensitivity analysis was performed where we used stricter repeatability criteria (see below).

## 2 Summary metrics

In the analysis protocol, we wrote a number of times that we would calculate “Median [95% prediction interval]” for variables. However, a prediction interval can be non-trivial to calculate if a variable is non-normally-distributed and some values are negative (as is the case with differences between spirometric indices from different devices). Hence, we decided to instead report median, $2.5^{th}$ percentile and $97.5^{th}$ percentile.

We also stated that we would report 95% confidence interval for the median (in practice, this would be the 95% confidence interval for the geometric mean). It is also non-trivial to calculate a geometric

mean with 95% confidence interval in case of skewed variables with negative values (as described above). Hence, we instead report arithmetic mean with 95% confidence interval.

# 3 Analyses of trends in bias

To characterize any trends in the FEV1 reported by the copd-6, we analyzed the difference between $FEV_1$ reported by the copd-6 and MicroDL devices as a function of the mean values from the two devices. The analysis protocol stated that we would analyze this in a linear regression model, i.e. a model of the following structure:

$$d = \beta_0 + \beta_1 \times m + \varepsilon$$

where $d$ is the difference between the two devices, $m$ is the mean, $\beta_0$ is the intercept, $\beta_1$ is the regression slope and $\varepsilon$ is an error term.

During data cleaning it became clear that a considerable proportion of the PEXADU project population were related to each other. To take into account this interdependence of data and to avoid underestimating the sizes of standard errors, we therefore decided to analyze the data in a mixed effect model of the following structure:

$$d = \beta_0 + \beta_1 \times m + \gamma + \varepsilon$$

where $\gamma$ is a random effect for family. We defined a family as a group of people where all persons were genetically related to at least one other member of the group.

To take non-linearity into account, we modelled the mean (m) using restricted cubic splines with four knots. This was described in the analysis protocol and is listed here for reference only.

# 4 Analysis based on $FEV_6$ and FVC instead of $FEV_1$

In the analysis protocol, we had specified that we would repeat the primary analysis, using $FEV_6$ from the copd-6 and FVC from the MicroDL instead of $FEV_1$ from both devices. We wrote that "this analysis will be limited to those persons where all acceptable blows with the MicroDL took ≤ 6 seconds (without adjusting for slow starts)." [1] However, very few persons fulfilled this criterion. We instead analyzed data for all the persons where the best FVC recorded with the MicroDL was from a curve that had reached a plateau and stopped before 6 seconds had passed (without adjusting for slow starts). I.e., the non-best curves did not have to reach a plateau or stop within 6 seconds.

## 5 Analysis with alternative quality criteria

To rule out that any differences between the MicroDL and copd-6 devices were due to the number of blows performed with each device (5-9 with the MicroDL, always 3 with the copd-6), we planned to repeat our analysis, based on the first three blows only for the MicroDL. Our analysis protocol stated that "For the MicroDL, we will only use the first three blows performed, and exclude blows where spirograms show slow start or cough. For the copd-6, we will use all three blows and exclude blows where the device showed a warning (because of either slow start or cough). We will base our analyses on only those participants who have three blows with the MicroDL and three blows with the copd-6 fulfilling these criteria."[1]

To ensure the maximum amount of data for analysis, without compromising on data quality, the inclusion criteria for this analysis were changed to the following:

- copd-6: Two blows or more have to be without warnings, and the difference in best and second-best $FEV_1$ and $FEV_6$ has to be < 0.25 liters.
- MicroDL: Use only the first three blows. Exclude examinations that do not have at least two "acceptable" blows according to ATS criteria [2] out of these three blows. Calculate best and second-best FEV1 and FVC based on the "usable" [2] blows only. The difference in best and second-best $FEV_1$ and FVC has to be < 0.25 liters.

## 6 Post-hoc analysis: Stricter repeatability criteria

As a post-hoc analysis, the main analysis was repeated with stricter criteria for repeatability:

| | **copd-6** | **MicroDL** |
|---|---|---|
| **$FEV_1$** | Δ < 0.15 liter<br>(0.10 liter if best $FEV_6$ < 1.00 liter) | Δ < 0.15 liter<br>(0.10 liter if best FVC < 1.00 liter) |
| **$FEV_6$** | Δ < 0.15 liter<br>(0.10 liter if best $FEV_6$ < 1.00 liter) | N/A |
| **FVC** | N/A | Δ < 0.15 liter<br>(0.10 liter if best FVC < 1.00 liter) |

Δ = difference between best and second-best value.

# 7 Post-hoc analysis: Analysis based on $FEV_1/FEV_6$

As a post-hoc analysis, we calculated the $FEV_1/FEV_6$ ratio for both the copd-6 and MicroDL spirometers, and compared the ratios in the same manner as we did with the absolute values of $FEV_1$. This analysis was limited to the same persons as the analysis based on $FEV_6$.

# 8 References for this appendix


1. Hansen MRH, Jørs E, Sandbæk A, Sekabojja D, Ssempebwa J, Mubeezi R, et al. Protocol for statistical analyses in the study entitled "Pesticide exposure, asthma and diabetes in Uganda (PEXADU)". Zenodo 2019. doi: 10.5281/zenodo.3386273.
2. Miller MR, Hankinson J, Brusasco V, Burgos F, Casaburi R, Coates A, et al. Standardisation of spirometry. European Respiratory Journal. 2005;26(2):319-38. doi: 10.1183/09031936.05.00034805.
