# Supplementary material for: Precision and accuracy of FEV1 measurements from the Vitalograph copd-6 mini-spirometer in a healthy Ugandan population
Source: PLoS One. 2021 Jun 28;16(6):e0253319. doi: 10.1371/journal.pone.0253319 (PMC8238209; doi:10.1371/journal.pone.0253319)
Supplement: S2 File — (PDF) [file pone.0253319.s002.pdf]

S2 File: Supplementary results for participant data

Contents

1 Stratified by order of testing 2

1.1 copd-6 before MicroDL 2

1.2 MicroDL before copd-6 3

2 Stratified by devices used 4

1.3 Excluded combinations of copd-6 and MicroDL devices 4

1.4 Combination of copd-6 device number 2 and MicroDL device number 2 4

1.5 Combination of copd-6 device number 2 and MicroDL device number 3 5

3 Analysis based on FEV_6 instead of FEV_1 6

4 Analysis based on FEV_1/FEV_6 7

5 Analysis restricted to first three blows with the MicroDL device 8

1.6 Overall results 8

1.7 Stratified by order of testing: copd-6 before MicroDL 9

1.8 Stratified by order of testing: MicroDL before copd-6 10

6 Analysis with stricter repeatability criteria 11

1 Stratified by order of testing

1.1 copd-6 before MicroDL

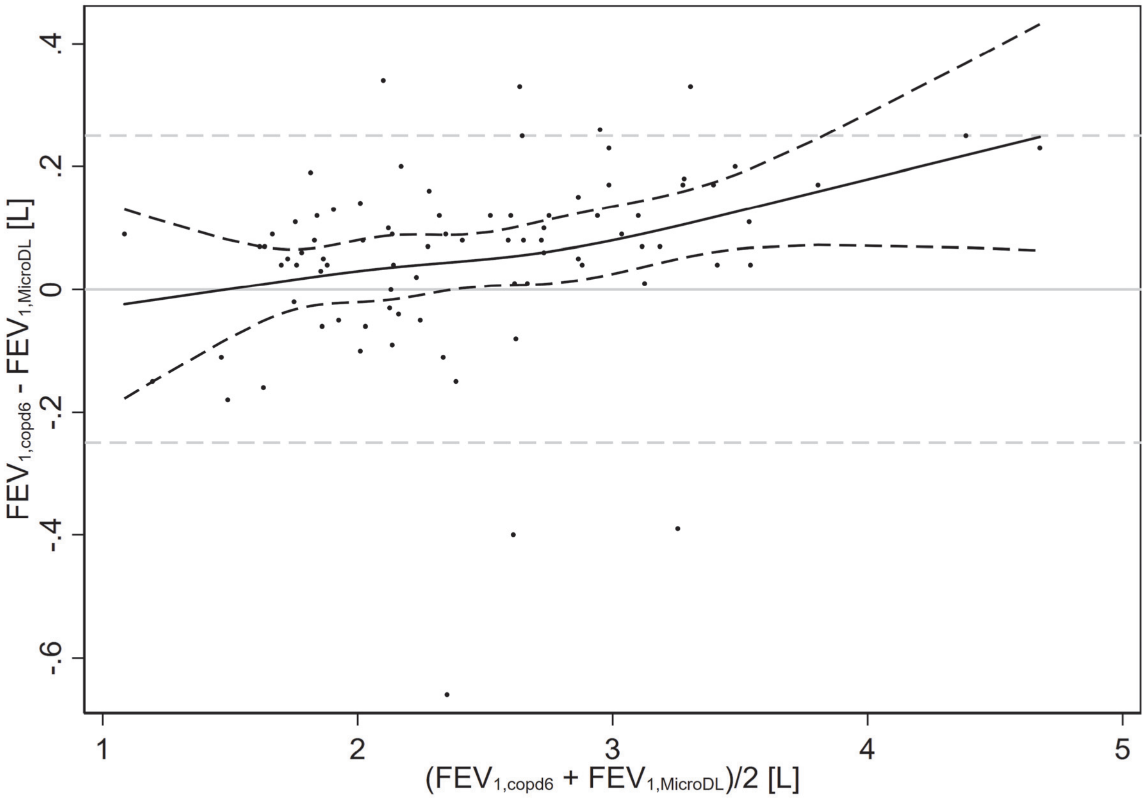

$FEV_{1,copd6}$ - $FEV_{1,MicroDL}$

Number of observations	84
Median	0.08
Interquartile range	[0.01 ; 0.12]
[2.5 percentile ; 97.5 percentile]	[-0.39 ; 0.33]
Range	[-0.66 ; 0.34]
Mean	0.05
95% confidence interval for mean	[0.02 ; 0.09]

1.2 MicroDL before copd-6

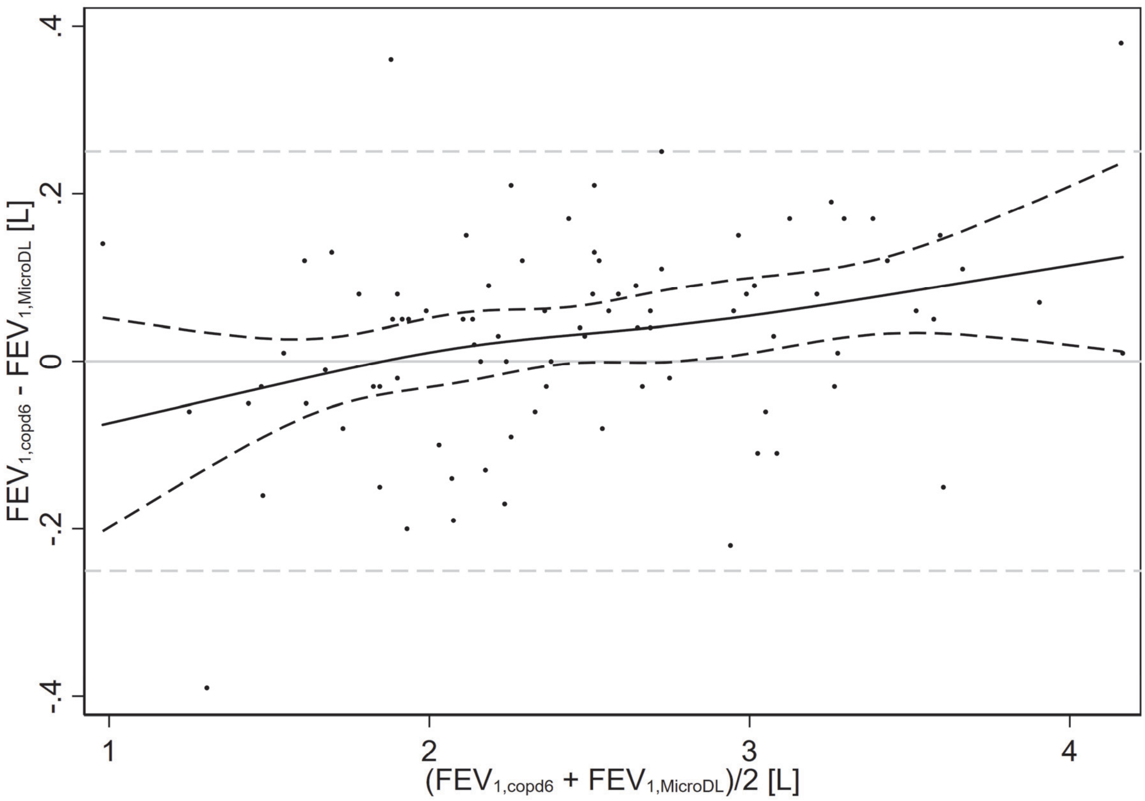

**$FEV_{1,copd6}$ - $FEV_{1,MicroDL}$**	
Number of observations	87
Median	0.05
Interquartile range	[-0.03 ; 0.11]
[2.5 percentile ; 97.5 percentile]	[-0.20 ; 0.25]
Range	[-0.39 ; 0.38]
Mean	0.03
95% confidence interval for mean	[0.00 ; 0.06]

2 Stratified by devices used

1.3 Excluded combinations of copd-6 and MicroDL devices

Eight individuals were excluded from these stratified analyses, because fewer than 15 persons had been tested with the same combination of devices and fulfilled quality criteria. This amount of data was deemed insufficient for statistical analysis, as pre-specified in the analysis protocol.

1.4 Combination of copd-6 device number 2 and MicroDL device number 2

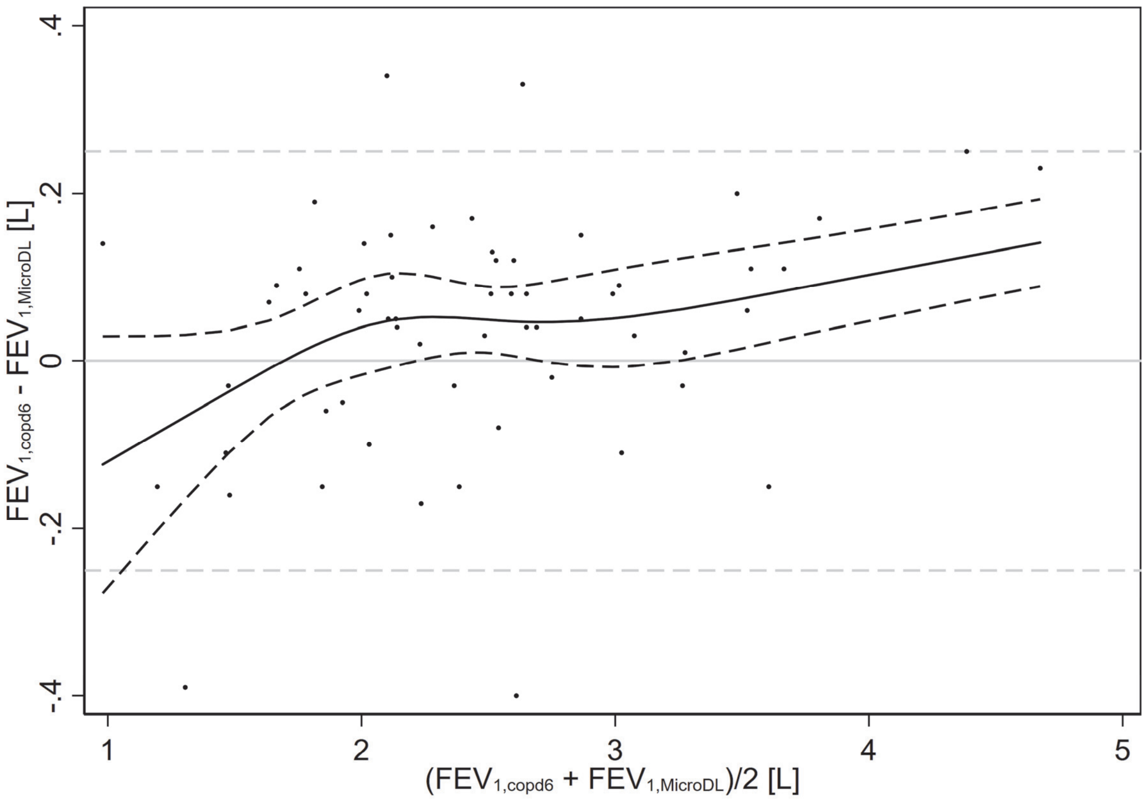

$FEV_{1,copd6}$ - $FEV_{1,MicroDL}$	
Number of observations	60
Median	0.06
Interquartile range	[-0.03 ; 0.12]
[2.5 percentile ; 97.5 percentile]	[-0.39 ; 0.33]
Range	[-0.40 ; 0.34]
Mean	0.04
95% confidence interval for mean	[0.00 ; 0.08]

1.5 Combination of copd-6 device number 2 and MicroDL device number 3

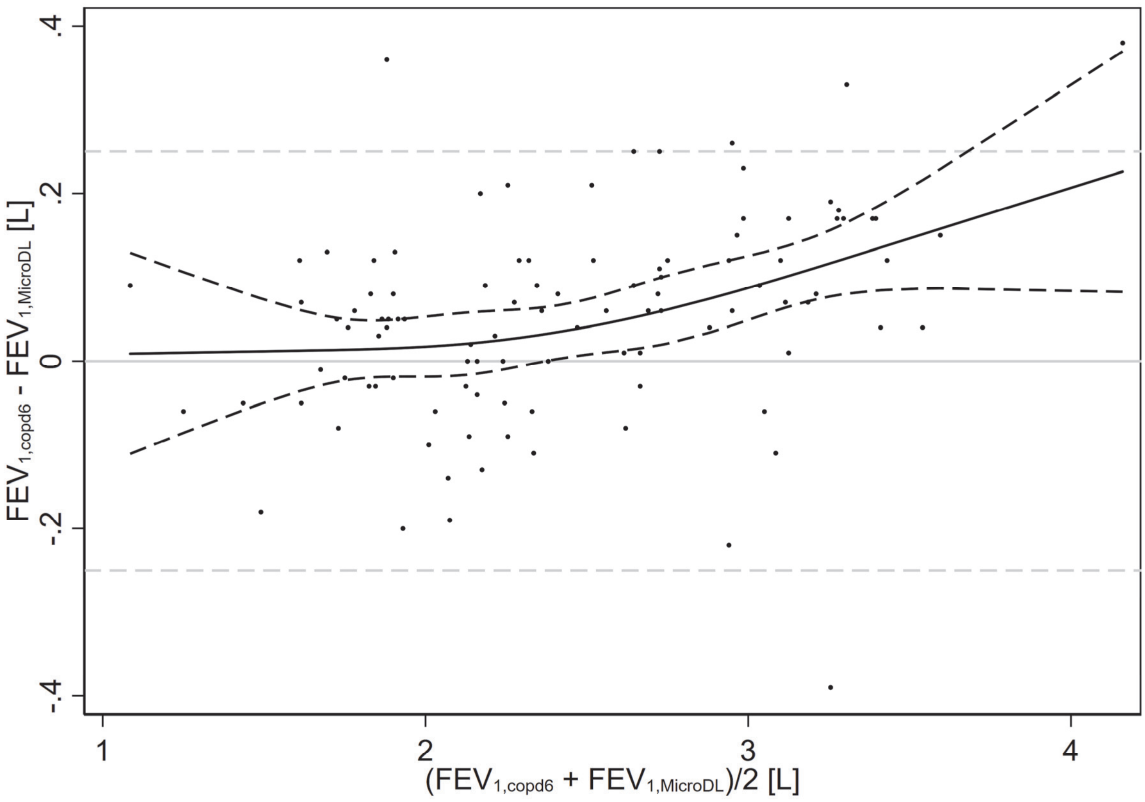

$FEV_{1,copd6}$ - $FEV_{1,MicroDL}$

Number of observations	103
Median	0.06
Interquartile range	[-0.03 ; 0.12]
[2.5 percentile ; 97.5 percentile]	[-0.20 ; 0.33]
Range	[-0.39 ; 0.38]
Mean	0.05
95% confidence interval for mean	[0.03 ; 0.08]

3 Analysis based on FEV_6 instead of FEV_1

The results below are based on data from 21 individuals who performed acceptable spirometry with both the copd-6 and the MicroDL devices, and whose best FVC with the MicroDL was from a blow that reached a plateau and stopped within 6 seconds of the start of the blow. In these cases, FEV_6 = FVC, meaning we can directly compare FEV_6 from the copd-6 with FVC from the MicroDL. Due to the low number of data points, no trend line has been plotted.

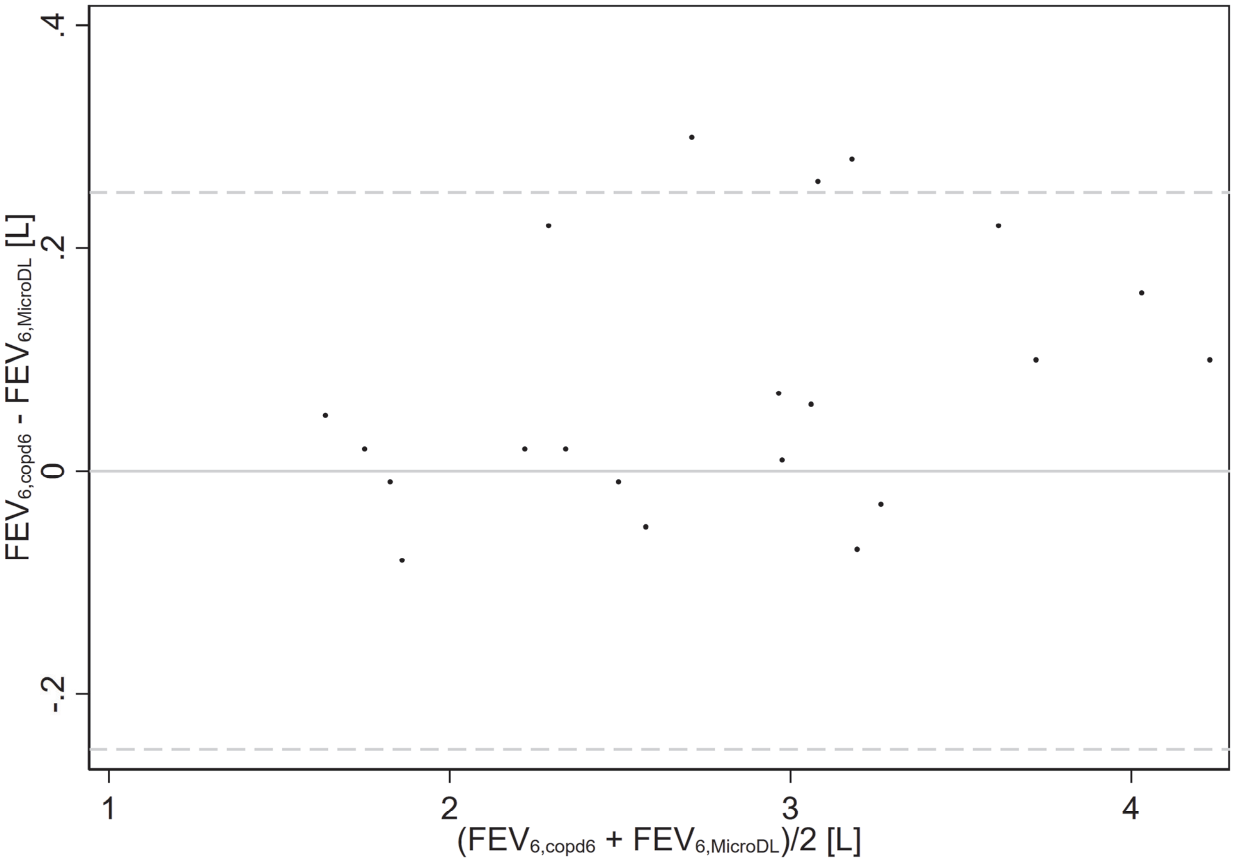

**$FEV_{6,copd6} – FEV_{6,MicroDL}$**	
Number of observations	21
Median	0.05
Interquartile range	[-0.01 ; 0.16]
95% prediction interval	[-0.08 ; 0.30]
Range	[-0.08 ; 0.30]
Mean	0.08
95% confidence interval for mean	[0.02 ; 0.13]

4 Analysis based on FEV_1/FEV_6

The results below are based on the 21 individuals as the preceding analysis. I.e., subjects who performed acceptable spirometry with both the MicroDL and copd-6 devices, and whose spirograms showed that they had FEV_6 = FVC. Due to the low number of points, no trend line has been fitted.

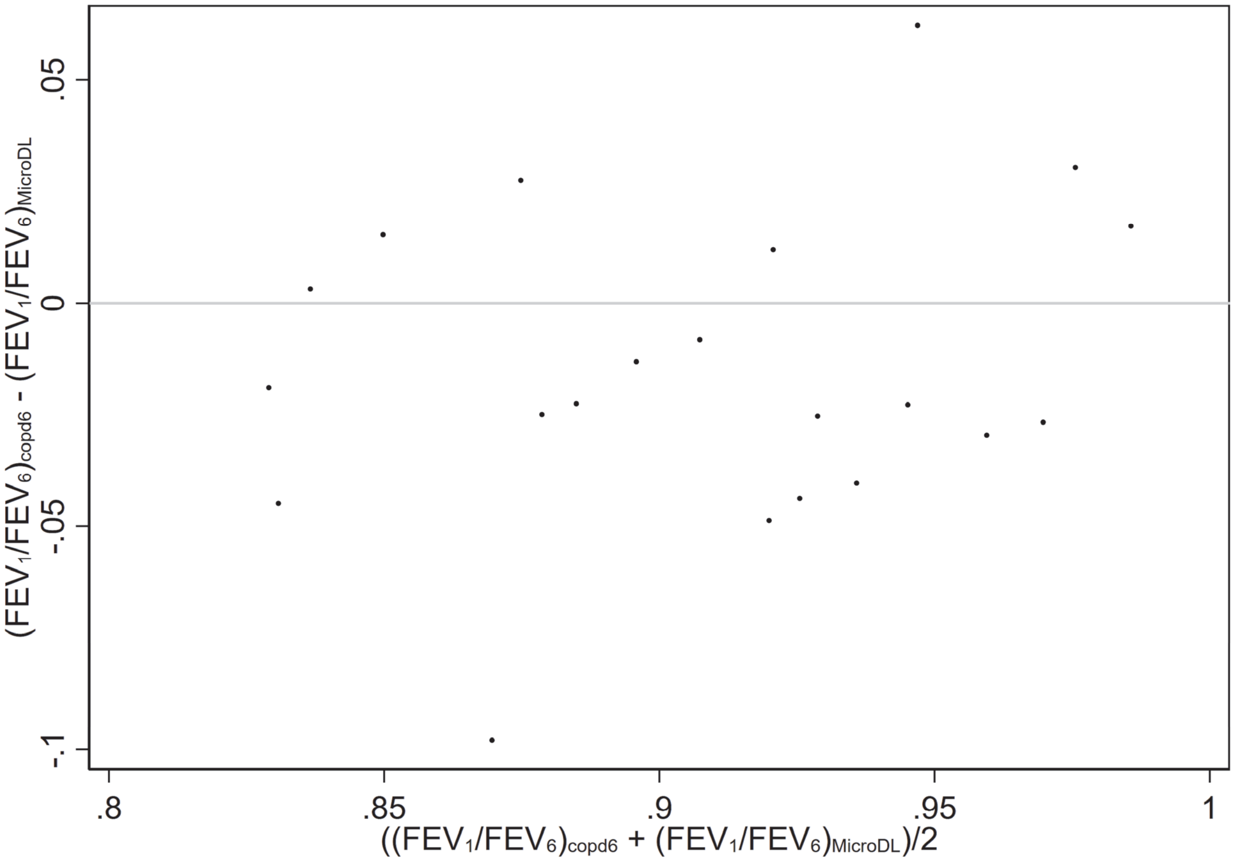

$(FEV_1/FEV_6)_{copd6} - (FEV_1/FEV_6)_{copd6}$	
Number of observations	21
Median	-0.02
Interquartile range	[-0.03 ; 0.01]
95% prediction interval	[-0.10 ; 0.06]
Range	[-0.10 ; 0.06]
Mean	-0.01
95% confidence interval for mean	[-0.03 ; 0.00]

5 Analysis restricted to first three blows with the MicroDL device

To rule out that any differences between the MicroDL and copd-6 devices were due to the number of blows performed with each device (5-9 with the MicroDL, always 3 with the copd-6), analyses were repeated based on the first three blows only for the MicroDL. Otherwise, the same quality criteria were applied as in the main analysis (at least 2 acceptable blows with the MicroDL, difference in best and second-best FEV_1 and FVC both < 0.25 liter).

1.6 Overall results

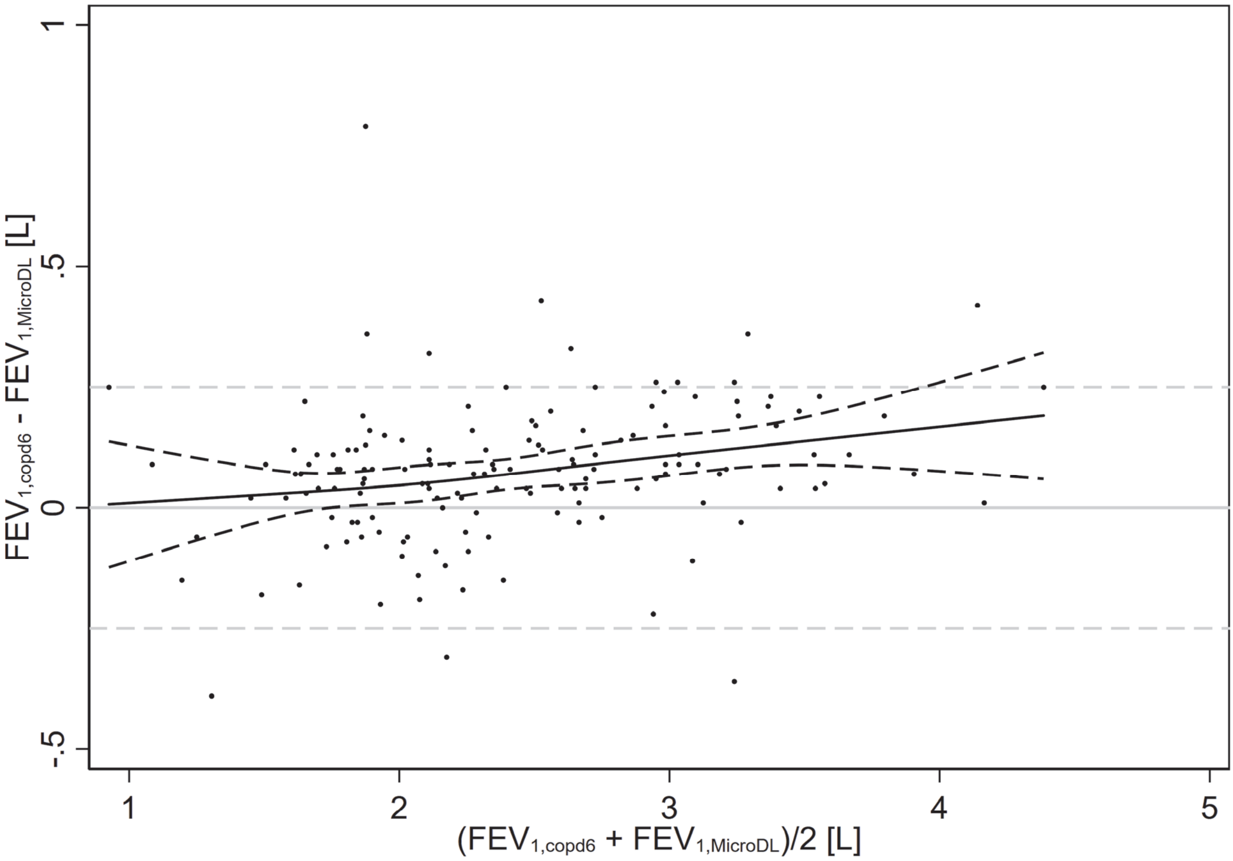

**$FEV_{1,copd6}$ - $FEV_{1,MicroDL}$**	
Number of observations	150
Median	0.08
Interquartile range	[0.01 ; 0.14]
[2.5 percentile ; 97.5 percentile]	[-0.22 ; 0.36]
Range	[-0.39 ; 0.79]
Mean	0.07
95% confidence interval for mean	[0.05 ; 0.10]

1.7 Stratified by order of testing: copd-6 before MicroDL

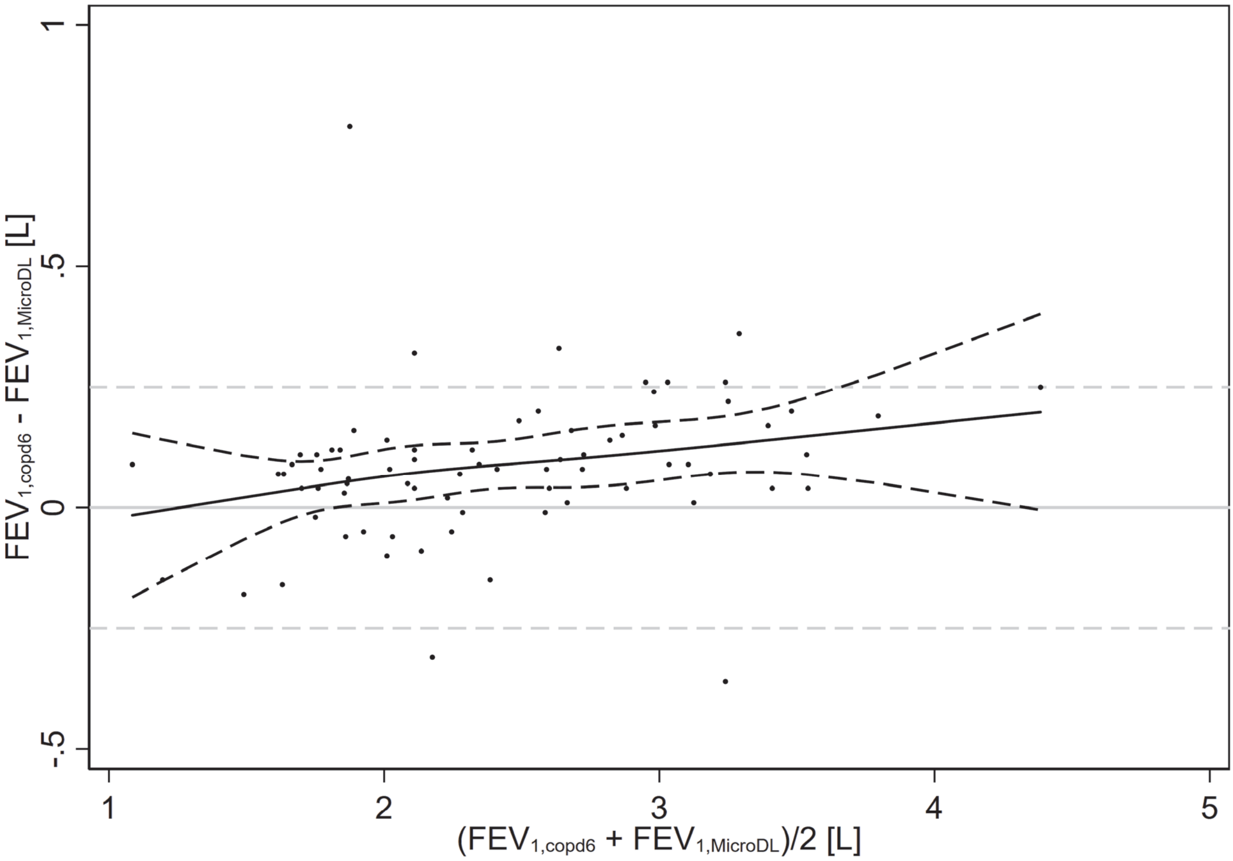

$FEV_{1,copd6}$ - $FEV_{1,MicroDL}$

Number of observations	74
Median	0.08
Interquartile range	[0.03 ; 0.15]
[2.5 percentile ; 97.5 percentile]	[-0.31 ; 0.36]
Range	[-0.36 ; 0.79]
Mean	0.08
95% confidence interval for mean	[0.05 ; 0.12]

1.8 Stratified by order of testing: MicroDL before copd-6

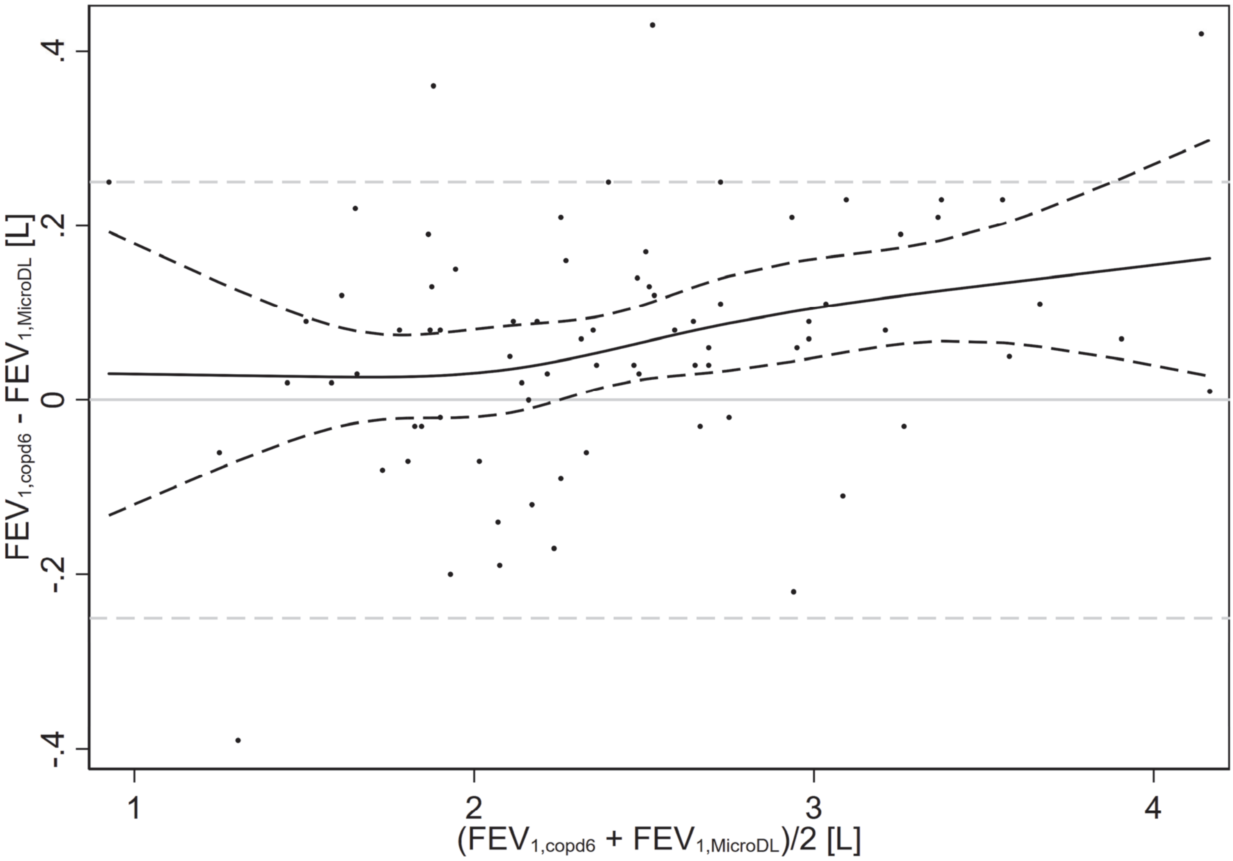

$FEV_{1,copd6}$ - $FEV_{1,MicroDL}$	
Number of observations	76
Median	0.07
Interquartile range	[-0.02 ; 0.13]
[2.5 percentile ; 97.5 percentile]	[-0.22 ; 0.42]
Range	[-0.39 ; 0.43]
Mean	0.06
95% confidence interval for mean	[0.03 ; 0.10]

6 Analysis with stricter repeatability criteria

For this sensitivity analysis, we used stricter repeatability criteria: For the copd-6, the difference between the best and second-best values of FEV_1 and FEV_6 had to be < 0.15 liters (< 0.10 liters if the best FEV_6 was < 1.00 liters). For the MicroDL, we used the same cut-offs, but for FVC instead of FEV_6.

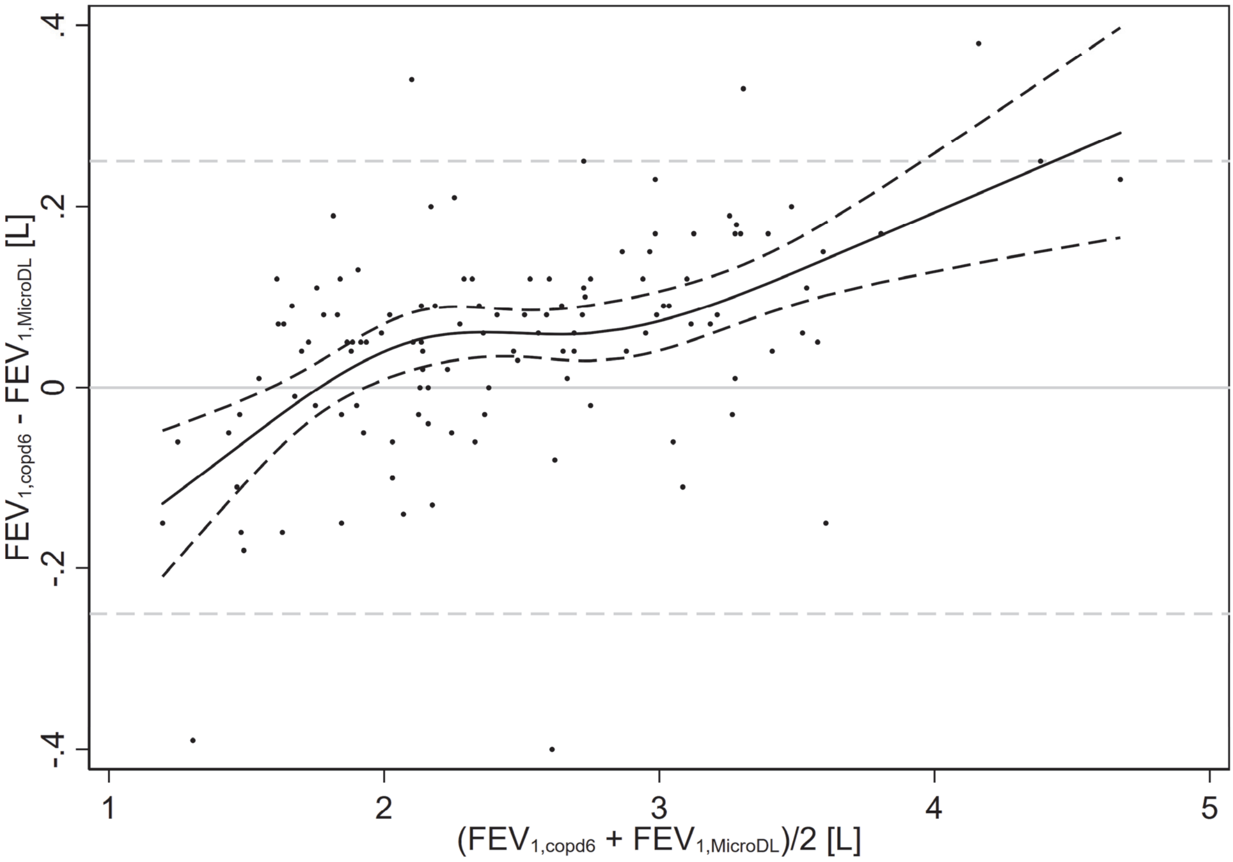

$FEV_{1,copd6}$ - $FEV_{1,MicroDL}$

Number of observations	120
Median	0.06
Interquartile range	[-0.01 ; 0.12]
[2.5 percentile ; 97.5 percentile]	[-0.17 ; 0.29]
Range	[-0.40 ; 0.38]
Mean	0.05
95% confidence interval for mean	[0.03 ; 0.07]
