# Supplementary material for: Precision and accuracy of FEV1 measurements from the Vitalograph copd-6 mini-spirometer in a healthy Ugandan population
Source: PLoS One. 2021 Jun 28;16(6):e0253319. doi: 10.1371/journal.pone.0253319 (PMC8238209; doi:10.1371/journal.pone.0253319)
Supplement: S3 File — (PDF) [file pone.0253319.s003.pdf]

S3 File: Supplementary results for copd-6 calibration check data

Summary statistics

The following table provides summary statistics for the calibration results, both overall and stratified by the speed of pushing the piston of the calibration syringe:

		**Slow**	**Medium**	**Fast**	**Total**
**Measurements**	n	102	102	102	306
**Reported FEV_1**	Geometric mean [95% CI]	0.83 [0.79; 0.87]	2.59 [2.49; 2.69]	3.10 [3.09; 3.11]	1.88 [1.76; 2.01]
**Reported FEV_6**	Geometric mean [95% CI]	2.92 [2.89; 2.95]	3.01 [2.97; 3.05]	3.11 [3.10; 3.11]	3.01 [2.99; 3.03]
**FEV_6 < 2.91 L**	n (%)	31 (30%)	1 (1%)	1 (1%)	33 (11%)
**FEV_6 > 3.09 L**	n (%)	0 (0%)	8 (8%)	59 (58%)	67 (22%)

For a graphical representation of these data, please refer to Fig 3 in the main text.

Scatterplots stratified by device used and day of testing

Two different copd-6 devices were used in the project. To check whether any imprecision or inaccuracy in the calibration check data was due to temperature differences between days, spirometer turbines wearing down, differences in device calibration etc., the analyses of calibration check data have been repeated with simultaneous stratification by day of testing and device used. The following pages list each stratified analysis separately.

Calibration data for copd-6: Day 1, device 2

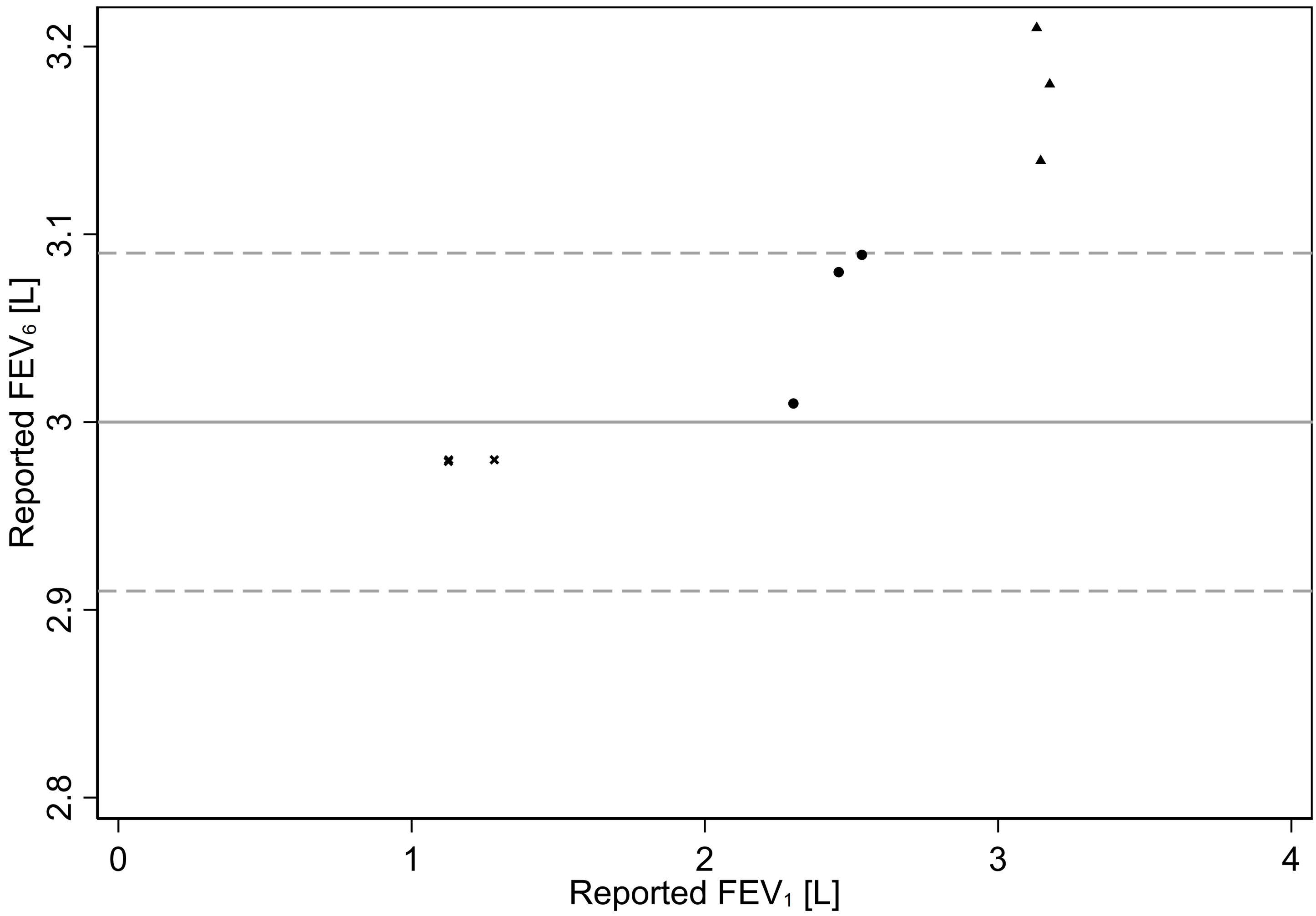

$\varrho_{Spearman}$ = 0.96, $p_{Spearman}$ < 0.001

Legend: × = slow, ● = medium, ▲ = fast

Gray lines = 3.00 L ± 3%

		Slow	Medium	Fast	Total
Measurements	n	3	3	3	9
Reported FEV_1	Geometric mean [95% CI]	1.17 [1.07; 1.29]	2.43 [2.30; 2.56]	3.15 [3.13; 3.17]	2.08 [1.55; 2.78]
Reported FEV_6	Geometric mean [95% CI]	2.98 [2.98; 2.98]	3.06 [3.01; 3.11]	3.18 [3.14; 3.22]	3.07 [3.01; 3.13]
Measurements with FEV_6 < 2.91 L	n (%)	0 (0%)	0 (0%)	0 (0%)	0 (0%)
Measurements with FEV_6 > 3.09 L	n (%)	0 (0%)	0 (0%)	3 (100%)	3 (33%)

Calibration data for copd-6: Day 2, device 1

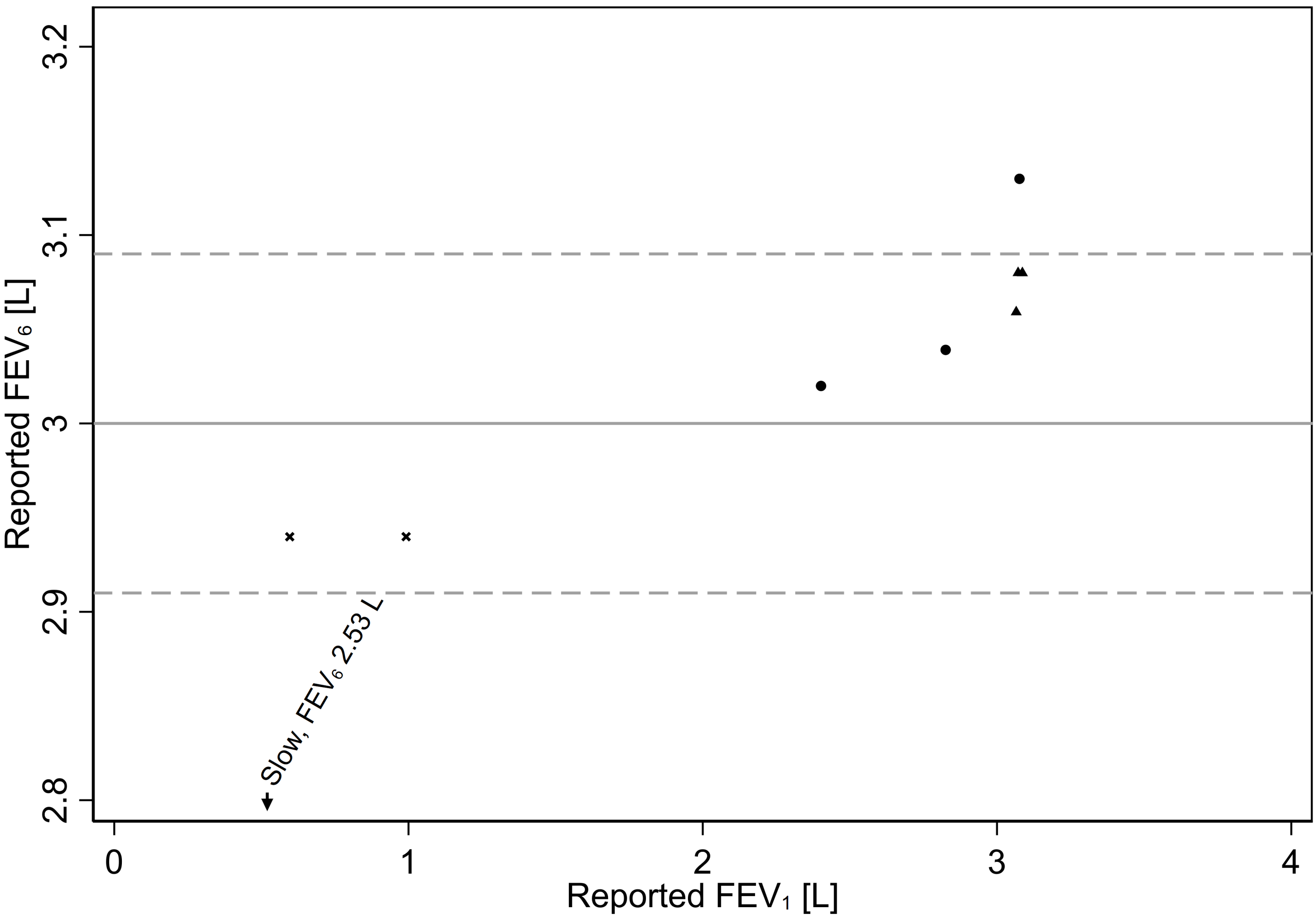

$\varrho_{Spearman} = 0.80$, $p_{Spearman} = 0.010$

Legend: × = slow, ● = medium, ▲ = fast

Gray lines = 3.00 L ± 3%

		Slow	Medium	Fast	Total
Measurements	n	3	3	3	9
Reported FEV_1	Geometric mean [95% CI]	0.67 [0.46; 1.00]	2.75 [2.40; 3.16]	3.07 [3.06; 3.09]	1.79 [1.09; 2.93]
Reported FEV_6	Geometric mean [95% CI]	2.80 [2.54; 3.08]	3.06 [3.00; 3.13]	3.07 [3.06; 3.09]	2.97 [2.85; 3.10]
Measurements with FEV_6 < 2.91 L	n (%)	1 (33%)	0 (0%)	0 (0%)	1 (11%)
Measurements with FEV_6 > 3.09 L	n (%)	0 (0%)	1 (33%)	0 (0%)	1 (11%)

Calibration data for copd-6: Day 2, device 2

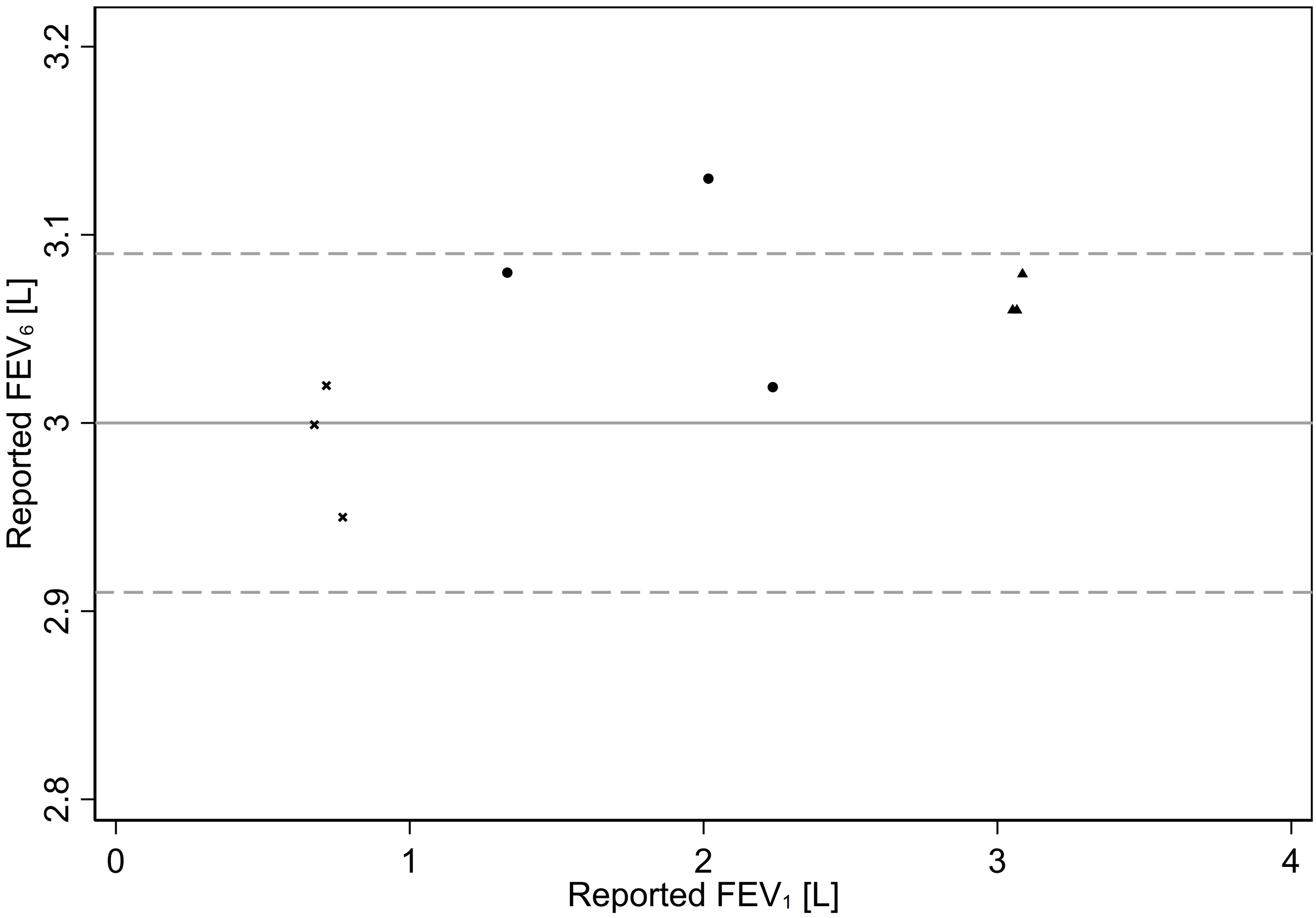

$\varrho_{Spearman} = 0.64$, $p_{Spearman} = 0.063$

Legend: × = slow, ● = medium, ▲ = fast

Gray lines = 3.00 L ± 3%

		Slow	Medium	Fast	Total
Measurements	n	3	3	3	9
Reported FEV_1	Geometric mean [95% CI]	0.72 [0.66; 0.78]	1.82 [1.34; 2.46]	3.07 [3.05; 3.08]	1.59 [1.04; 2.43]
Reported FEV_6	Geometric mean [95% CI]	2.99 [2.95; 3.03]	3.08 [3.01; 3.14]	3.07 [3.05; 3.08]	3.04 [3.01; 3.08]
Measurements with $FEV_6 < 2.91$ L	n (%)	0 (0%)	0 (0%)	0 (0%)	0 (0%)
Measurements with $FEV_6 > 3.09$ L	n (%)	0 (0%)	1 (33%)	0 (0%)	1 (11%)

Calibration data for copd-6: Day 3, device 1

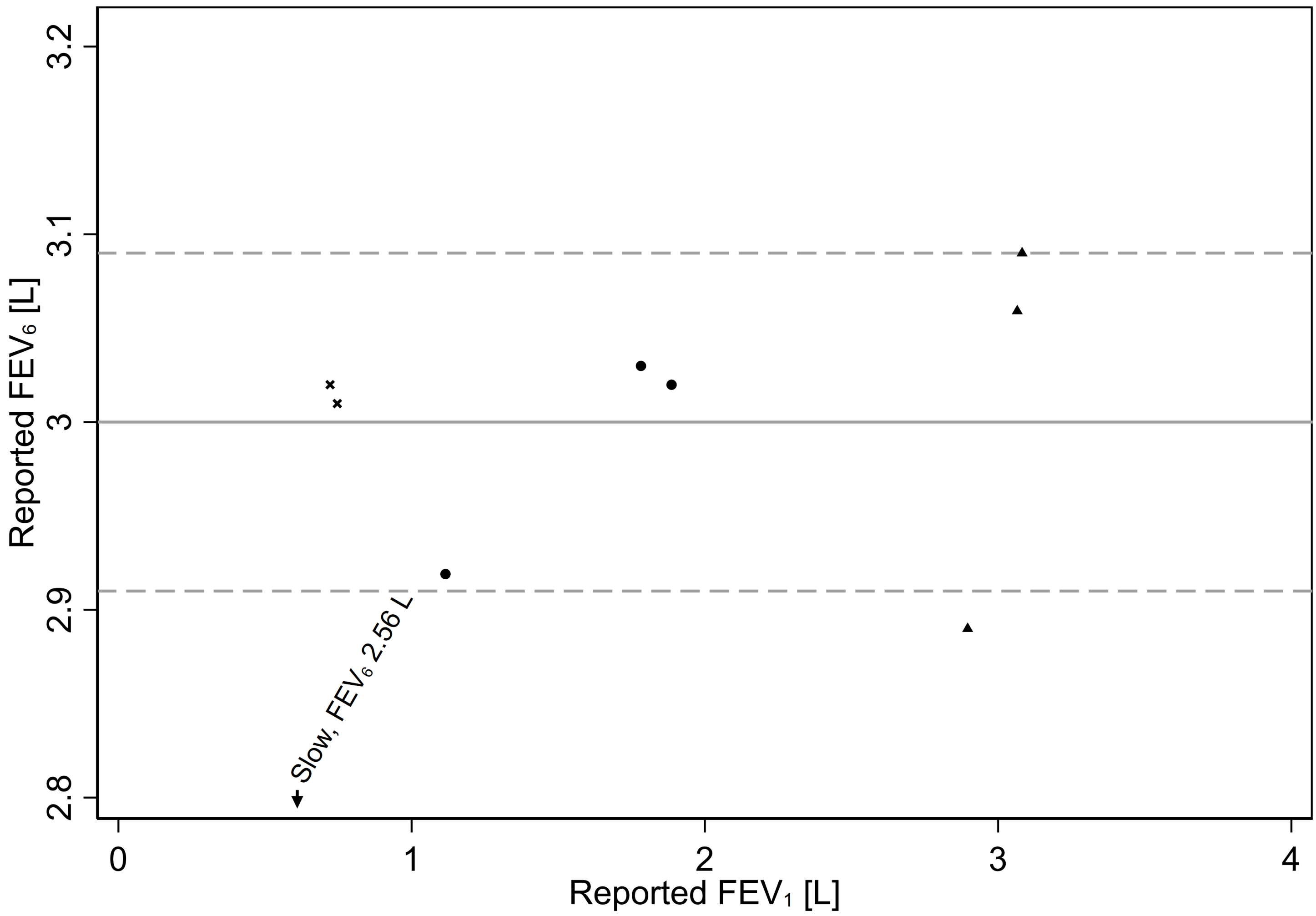

$\varrho_{Spearman} = 0.45$, $p_{Spearman} = 0.224$

Legend: × = slow, ● = medium, ▲ = fast

Gray lines = 3.00 L ± 3%

		Slow	Medium	Fast	Total
Measurements	n	3	3	3	9
Reported FEV_1	Geometric mean [95% CI]	0.69 [0.61; 0.78]	1.55 [1.12; 2.16]	3.01 [2.89; 3.14]	1.48 [0.96; 2.27]
Reported FEV_6	Geometric mean [95% CI]	2.85 [2.57; 3.18]	2.99 [2.92; 3.06]	3.01 [2.89; 3.14]	2.95 [2.84; 3.06]
Measurements with FEV_6 < 2.91 L	n (%)	1 (33%)	0 (0%)	1 (33%)	2 (22%)
Measurements with FEV_6 > 3.09 L	n (%)	0 (0%)	0 (0%)	0 (0%)	0 (0%)

Calibration data for copd-6: Day 3, device 2

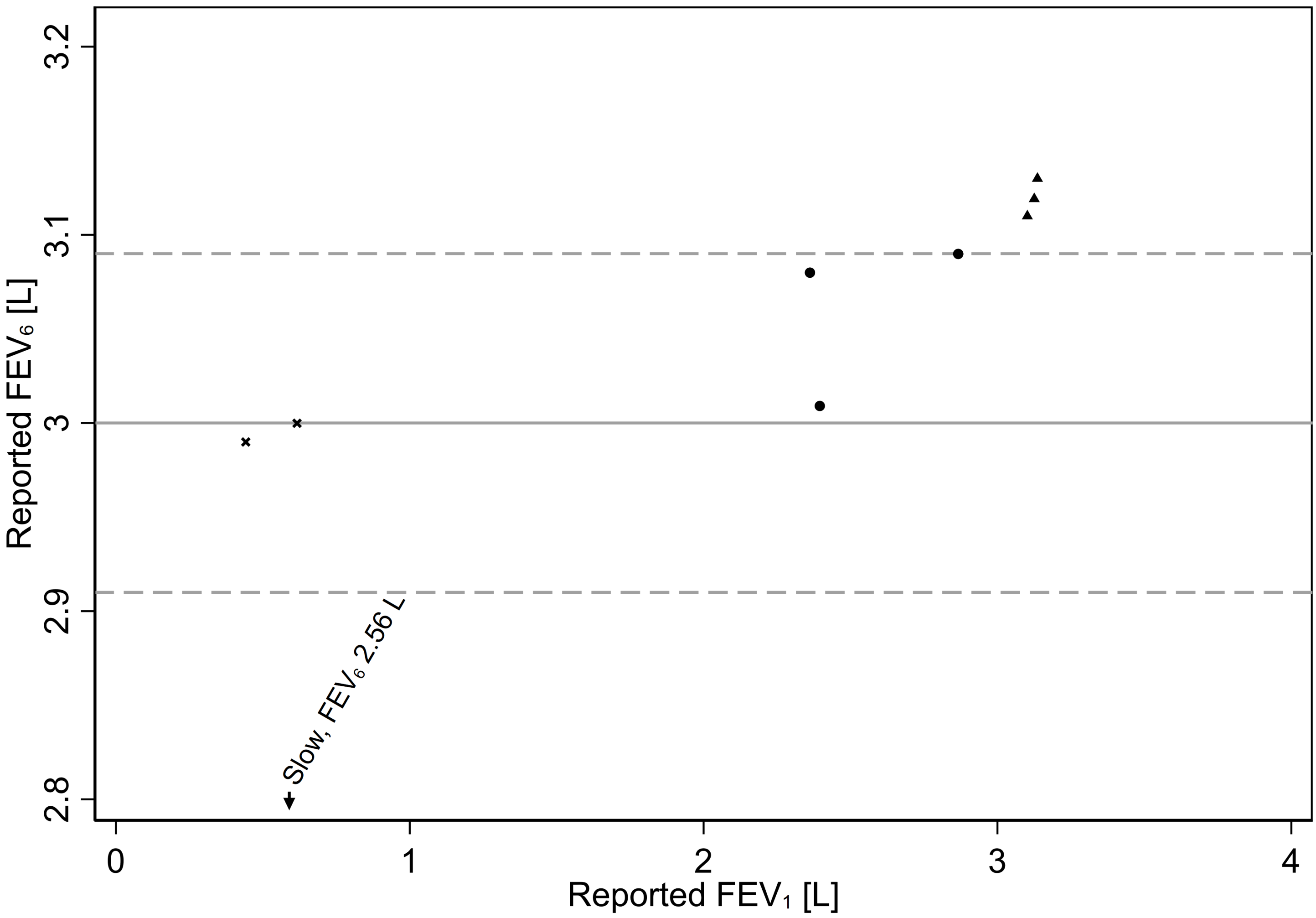

$\varrho_{Spearman} = 0.95$, $p_{Spearman} < 0.001$

Legend: × = slow, ● = medium, ▲ = fast

Gray lines = 3.00 L ± 3%

		Slow	Medium	Fast	Total
Measurements	n	3	3	3	9
Reported FEV_1	Geometric mean [95% CI]	0.55 [0.45; 0.66]	2.53 [2.24; 2.85]	3.12 [3.11; 3.13]	1.63 [0.95; 2.80]
Reported FEV_6	Geometric mean [95% CI]	2.84 [2.57; 3.15]	3.06 [3.01; 3.11]	3.12 [3.11; 3.13]	3.00 [2.88; 3.13]
Measurements with FEV_6 < 2.91 L	n (%)	1 (33%)	0 (0%)	0 (0%)	1 (11%)
Measurements with FEV_6 > 3.09 L	n (%)	0 (0%)	0 (0%)	3 (100%)	3 (33%)

Calibration data for copd-6: Day 4, device 1

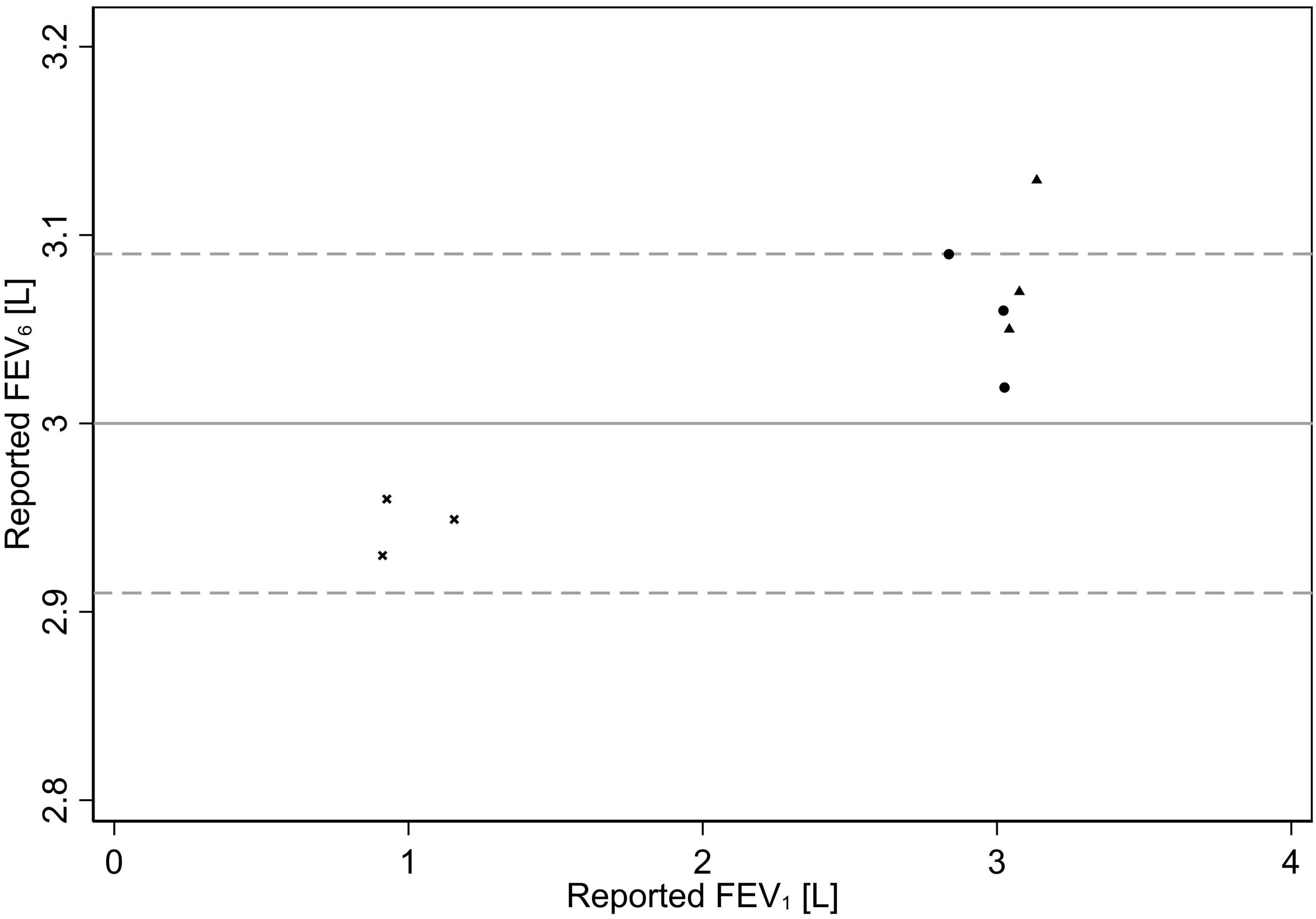

$\varrho_{Spearman}$ = 0.79, $p_{Spearman}$ = 0.011

Legend: × = slow, ● = medium, ▲ = fast

Gray lines = 3.00 L ± 3%

		Slow	Medium	Fast	Total
Measurements	n	3	3	3	9
Reported FEV_1	Geometric mean [95% CI]	0.99 [0.86; 1.15]	2.96 [2.83; 3.09]	3.08 [3.04; 3.13]	2.08 [1.44; 3.01]
Reported FEV_6	Geometric mean [95% CI]	2.95 [2.93; 2.96]	3.06 [3.02; 3.10]	3.08 [3.04; 3.13]	3.03 [2.98; 3.07]
Measurements with FEV_6 < 2.91 L	n (%)	0 (0%)	0 (0%)	0 (0%)	0 (0%)
Measurements with FEV_6 > 3.09 L	n (%)	0 (0%)	0 (0%)	1 (33%)	1 (11%)

Calibration data for copd-6: Day 4, device 2

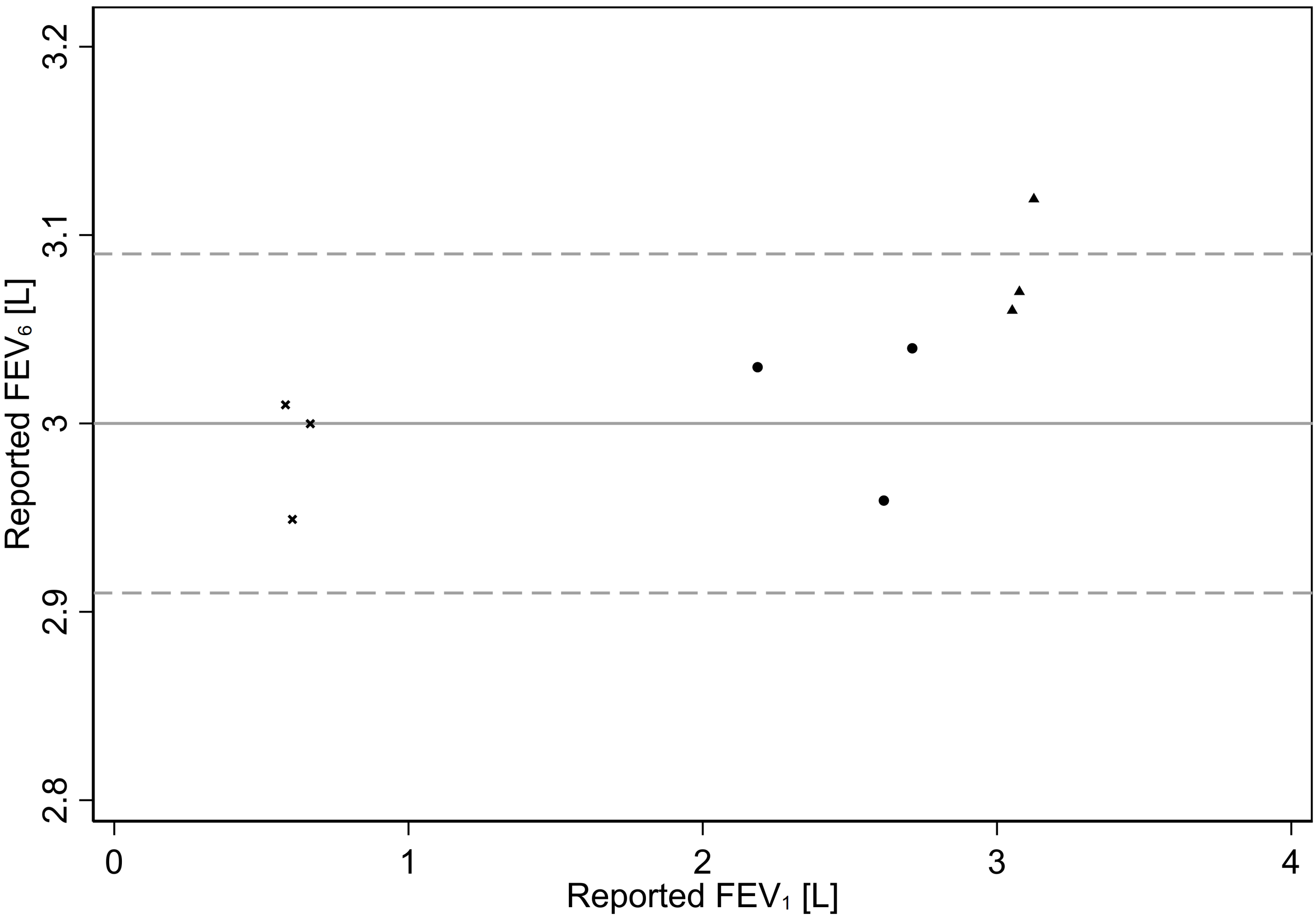

$\varrho_{Spearman}$ = 0.84, $p_{Spearman}$ = 0.004

Legend: × = slow, ● = medium, ▲ = fast

Gray lines = 3.00 L ± 3%

		Slow	Medium	Fast	Total
Measurements	n	3	3	3	9
Reported FEV_1	Geometric mean [95% CI]	0.62 [0.58; 0.66]	2.49 [2.18; 2.85]	3.08 [3.05; 3.12]	1.68 [1.02; 2.76]
Reported FEV_6	Geometric mean [95% CI]	2.99 [2.95; 3.02]	3.01 [2.96; 3.06]	3.08 [3.05; 3.12]	3.03 [2.99; 3.06]
Measurements with FEV_6 < 2.91 L	n (%)	0 (0%)	0 (0%)	0 (0%)	0 (0%)
Measurements with FEV_6 > 3.09 L	n (%)	0 (0%)	0 (0%)	1 (33%)	1 (11%)

Calibration data for copd-6: Day 5, device 1

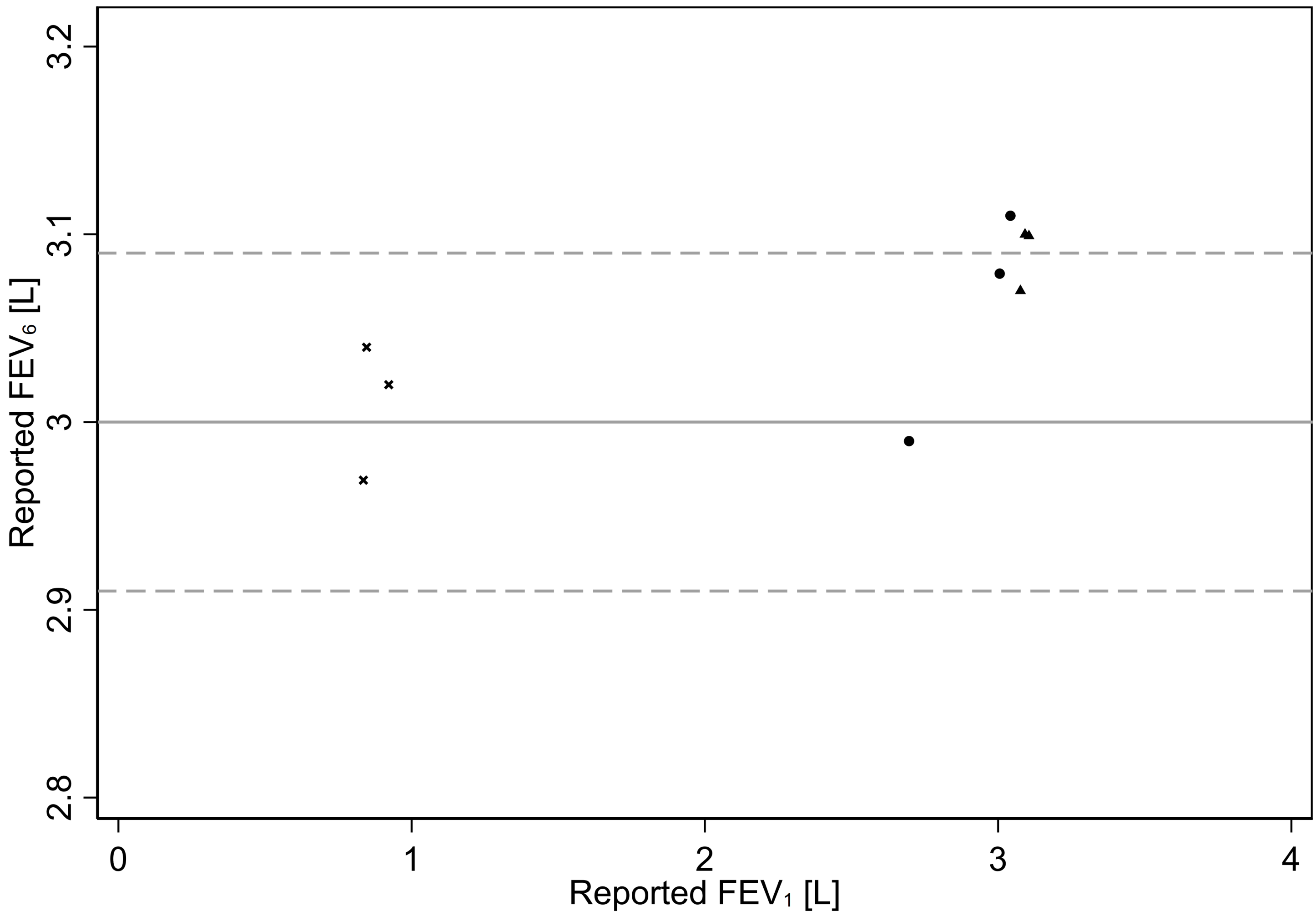

$\varrho_{Spearman}$ = 0.64, $p_{Spearman}$ = 0.066

Legend: × = slow, ● = medium, ▲ = fast

Gray lines = 3.00 L ± 3%

		Slow	Medium	Fast	Total
Measurements	n	3	3	3	9
Reported FEV_1	Geometric mean [95% CI]	0.87 [0.81; 0.93]	2.91 [2.69; 3.14]	3.09 [3.07; 3.11]	1.98 [1.32; 2.98]
Reported FEV_6	Geometric mean [95% CI]	3.01 [2.97; 3.05]	3.06 [2.99; 3.13]	3.09 [3.07; 3.11]	3.05 [3.02; 3.09]
Measurements with FEV_6 < 2.91 L	n (%)	0 (0%)	0 (0%)	0 (0%)	0 (0%)
Measurements with FEV_6 > 3.09 L	n (%)	0 (0%)	1 (33%)	2 (67%)	3 (33%)

Calibration data for copd-6: Day 5, device 2

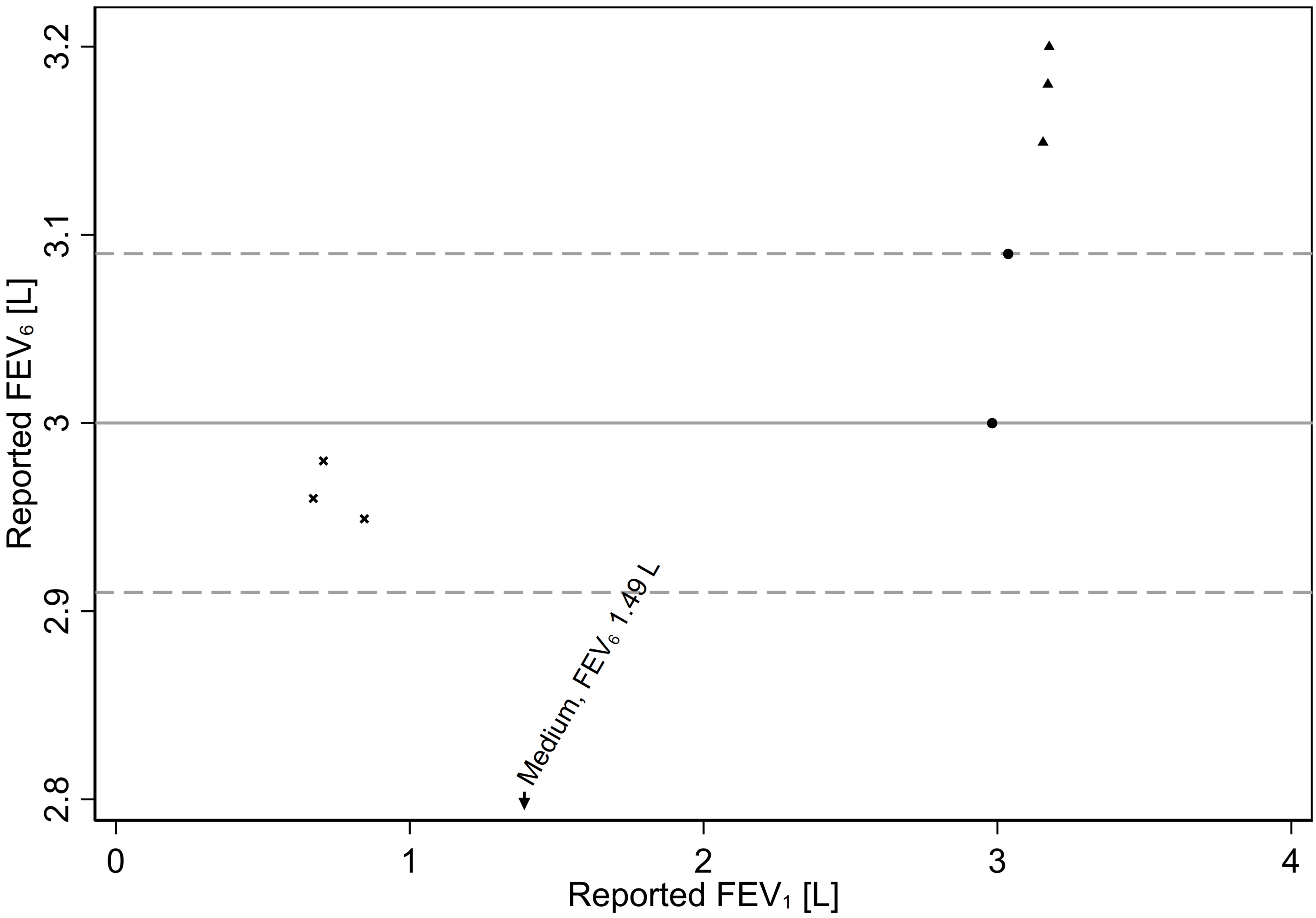

$\varrho_{Spearman} = 0.79$, $p_{Spearman} = 0.011$

Legend: × = slow, ● = medium, ▲ = fast

Gray lines = 3.00 L ± 3%

		Slow	Medium	Fast	Total
Measurements	n	3	3	3	9
Reported FEV_1	Geometric mean [95% CI]	0.74 [0.65; 0.84]	2.33 [1.40; 3.85]	3.17 [3.15; 3.18]	1.76 [1.11; 2.78]
Reported FEV_6	Geometric mean [95% CI]	2.96 [2.95; 2.98]	2.40 [1.50; 3.83]	3.18 [3.15; 3.21]	2.83 [2.41; 3.31]
Measurements with FEV_6 < 2.91 L	n (%)	0 (0%)	1 (33%)	0 (0%)	1 (11%)
Measurements with FEV_6 > 3.09 L	n (%)	0 (0%)	0 (0%)	3 (100%)	3 (33%)

Calibration data for copd-6: Day 6, device 1

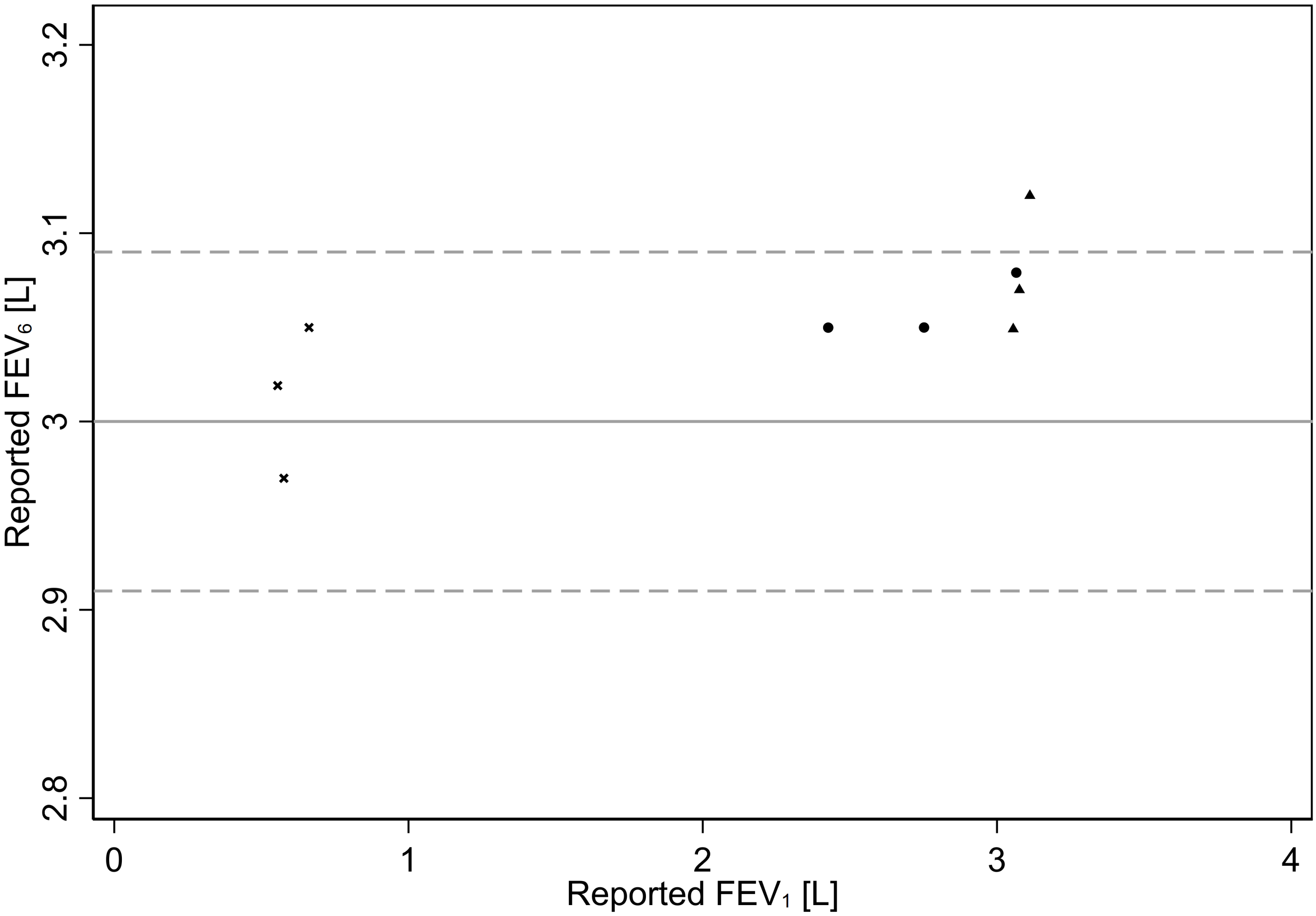

$\varrho_{Spearman} = 0.72$, $p_{Spearman} = 0.030$

Legend: × = slow, ● = medium, ▲ = fast

Gray lines = 3.00 L ± 3%

		Slow	Medium	Fast	Total
Measurements	n	3	3	3	9
Reported FEV_1	Geometric mean [95% CI]	0.59 [0.53; 0.67]	2.73 [2.39; 3.12]	3.08 [3.04; 3.12]	1.71 [1.02; 2.88]
Reported FEV_6	Geometric mean [95% CI]	3.01 [2.97; 3.06]	3.06 [3.04; 3.08]	3.08 [3.04; 3.12]	3.05 [3.02; 3.08]
Measurements with FEV_6 < 2.91 L	n (%)	0 (0%)	0 (0%)	0 (0%)	0 (0%)
Measurements with FEV_6 > 3.09 L	n (%)	0 (0%)	0 (0%)	1 (33%)	1 (11%)

Calibration data for copd-6: Day 6, device 2

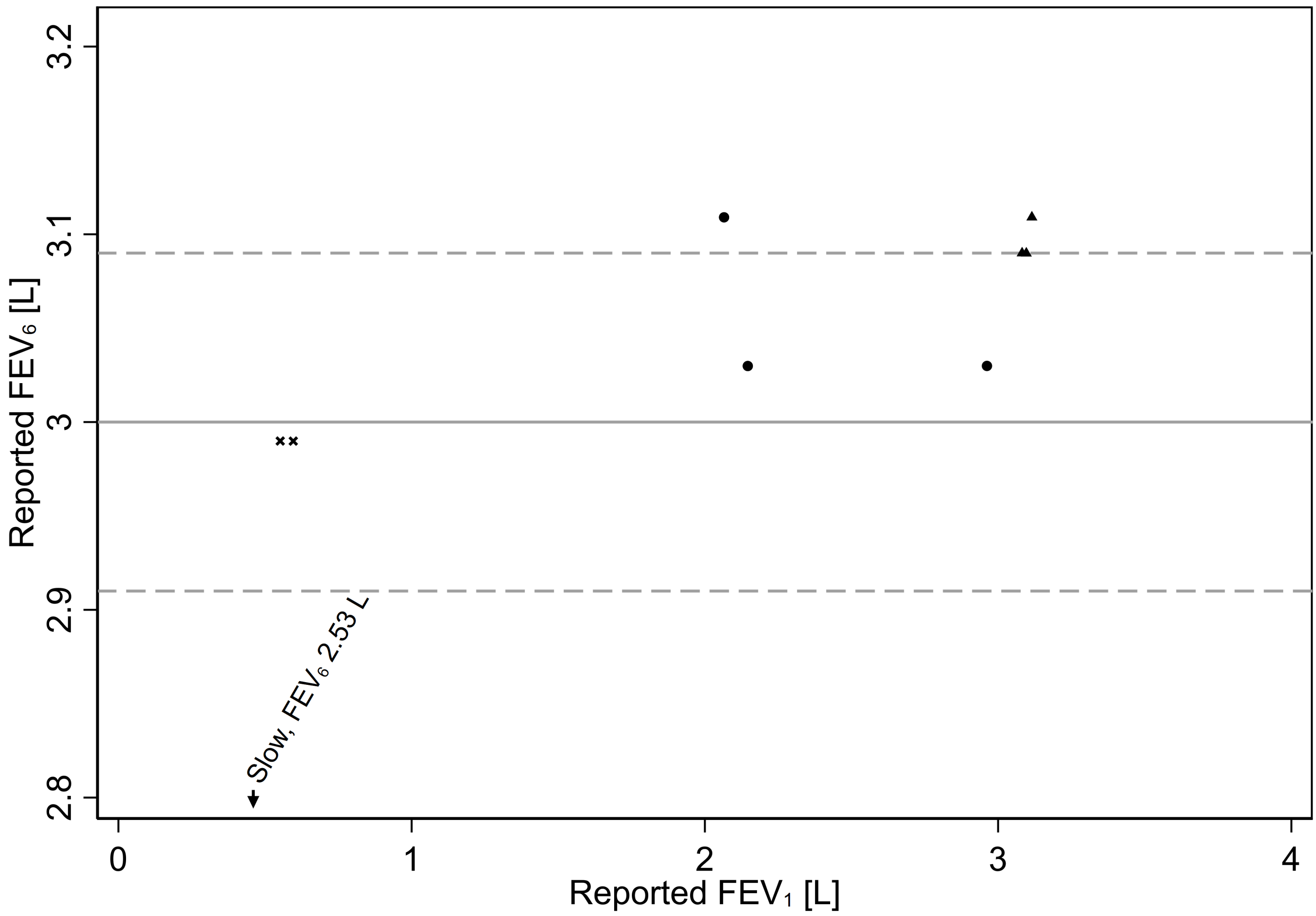

$\varrho_{Spearman}$ = 0.83, $p_{Spearman}$ = 0.006

Legend: × = slow, ● = medium, ▲ = fast

Gray lines = 3.00 L ± 3%

		Slow	Medium	Fast	Total
Measurements	n	3	3	3	9
Reported FEV_1	Geometric mean [95% CI]	0.53 [0.46; 0.62]	2.36 [1.88; 2.96]	3.10 [3.08; 3.11]	1.57 [0.92; 2.70]
Reported FEV_6	Geometric mean [95% CI]	2.83 [2.54; 3.15]	3.06 [3.00; 3.11]	3.10 [3.08; 3.11]	2.99 [2.87; 3.12]
Measurements with FEV_6 < 2.91 L	n (%)	1 (33%)	0 (0%)	0 (0%)	1 (11%)
Measurements with FEV_6 > 3.09 L	n (%)	0 (0%)	1 (33%)	1 (33%)	2 (22%)

Calibration data for copd-6: Day 7, device 1

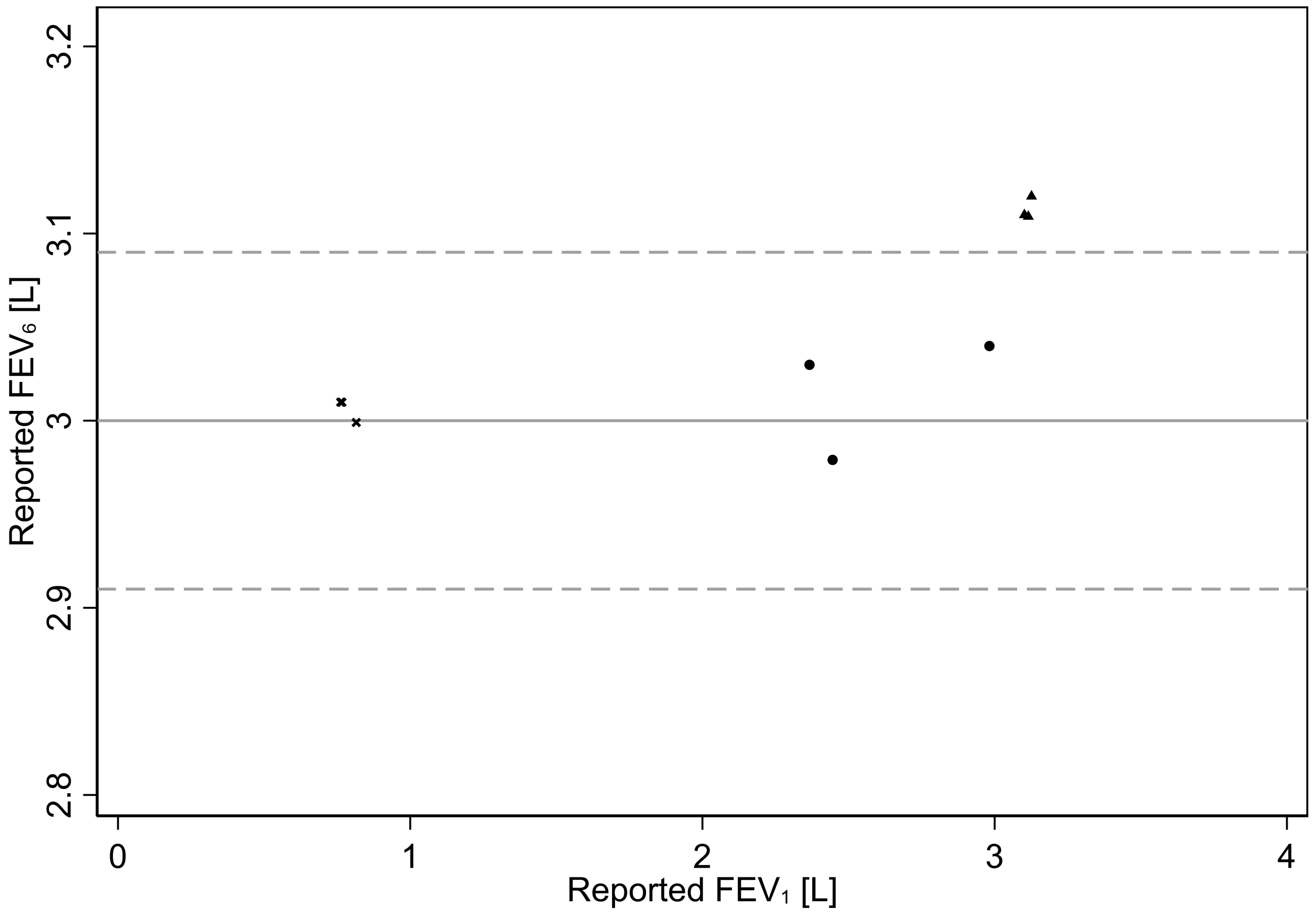

$\varrho_{Spearman} = 0.80$, $p_{Spearman} = 0.010$

Legend: × = slow, ● = medium, ▲ = fast

Gray lines = 3.00 L ± 3%

		Slow	Medium	Fast	Total
Measurements	n	3	3	3	9
Reported FEV_1	Geometric mean [95% CI]	0.78 [0.75; 0.81]	2.58 [2.23; 2.98]	3.11 [3.11; 3.12]	1.84 [1.20; 2.83]
Reported FEV_6	Geometric mean [95% CI]	3.01 [3.00; 3.01]	3.02 [2.98; 3.05]	3.11 [3.11; 3.12]	3.05 [3.01; 3.08]
Measurements with FEV_6 < 2.91 L	n (%)	0 (0%)	0 (0%)	0 (0%)	0 (0%)
Measurements with FEV_6 > 3.09 L	n (%)	0 (0%)	0 (0%)	3 (100%)	3 (33%)

Calibration data for copd-6: Day 7, device 2

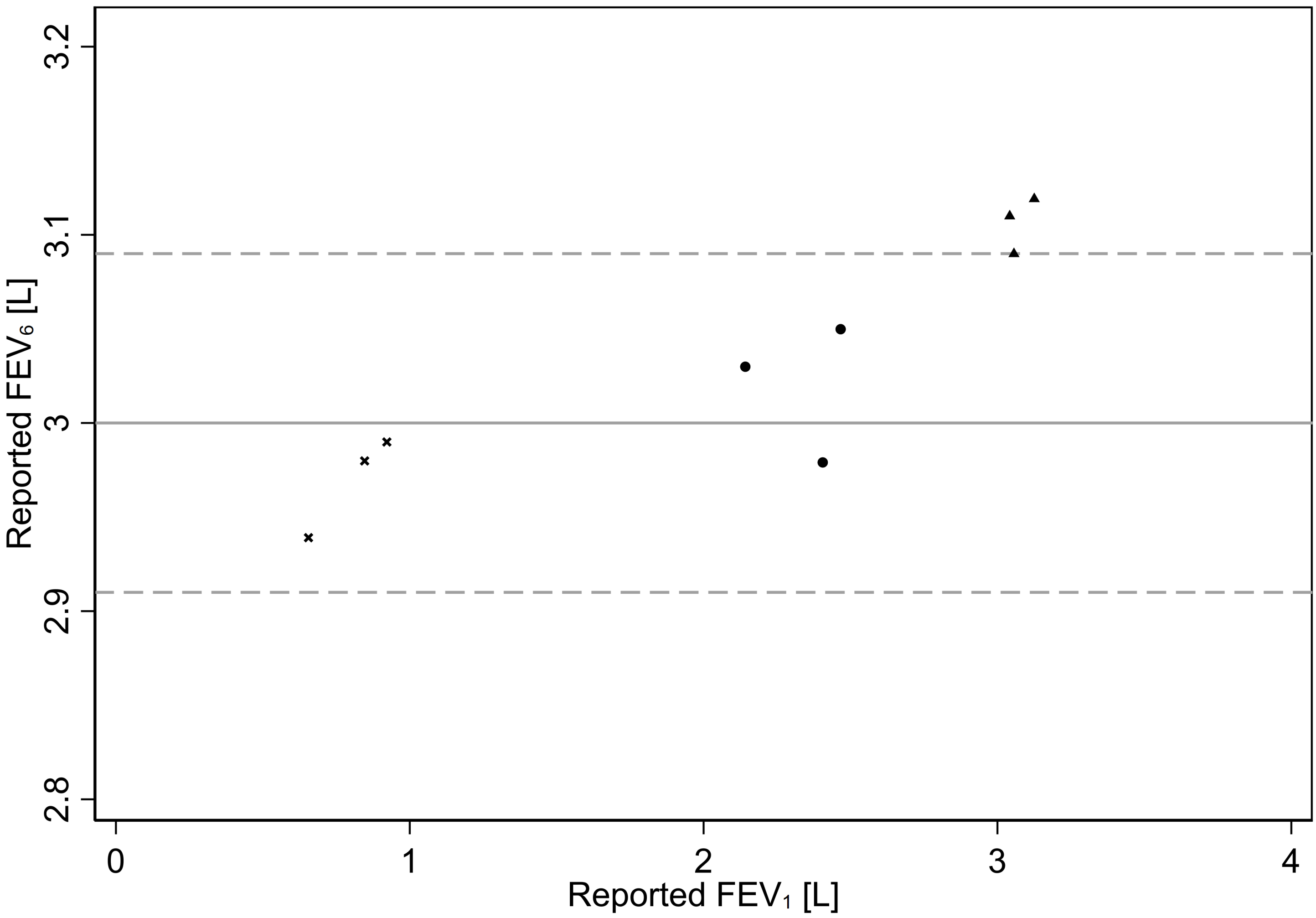

$\varrho_{Spearman} = 0.87$, $p_{Spearman} = 0.002$

Legend: × = slow, ● = medium, ▲ = fast

Gray lines = 3.00 L ± 3%

		Slow	Medium	Fast	Total
Measurements	n	3	3	3	9
Reported FEV_1	Geometric mean [95% CI]	0.80 [0.65; 0.98]	2.33 [2.15; 2.53]	3.07 [3.03; 3.12]	1.79 [1.19; 2.69]
Reported FEV_6	Geometric mean [95% CI]	2.97 [2.94; 3.00]	3.02 [2.98; 3.06]	3.11 [3.09; 3.12]	3.03 [2.99; 3.07]
Measurements with FEV_6 < 2.91 L	n (%)	0 (0%)	0 (0%)	0 (0%)	0 (0%)
Measurements with FEV_6 > 3.09 L	n (%)	0 (0%)	0 (0%)	2 (67%)	2 (22%)

Calibration data for copd-6: Day 8, device 1

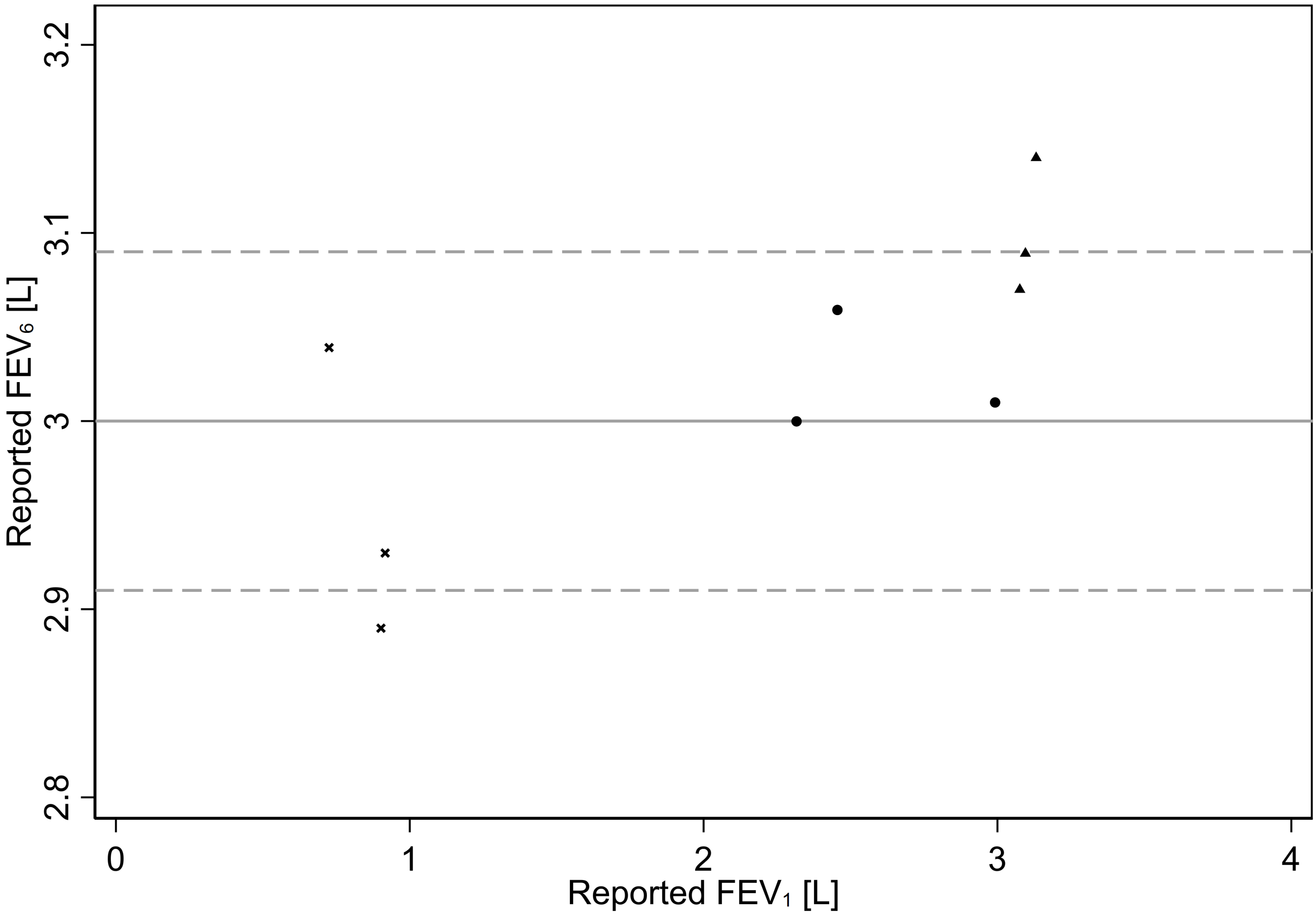

$\varrho_{Spearman} = 0.84$, $p_{Spearman} = 0.004$

Legend: × = slow, ● = medium, ▲ = fast

Gray lines = 3.00 L ± 3%

		Slow	Medium	Fast	Total
Measurements	n	3	3	3	9
Reported FEV_1	Geometric mean [95% CI]	0.84 [0.72; 0.98]	2.57 [2.20; 3.00]	3.10 [3.06; 3.14]	1.89 [1.26; 2.82]
Reported FEV_6	Geometric mean [95% CI]	2.95 [2.87; 3.04]	3.02 [2.99; 3.06]	3.10 [3.06; 3.14]	3.02 [2.97; 3.08]
Measurements with FEV_6 < 2.91 L	n (%)	1 (33%)	0 (0%)	0 (0%)	1 (11%)
Measurements with FEV_6 > 3.09 L	n (%)	0 (0%)	0 (0%)	1 (33%)	1 (11%)

Calibration data for copd-6: Day 8, device 2

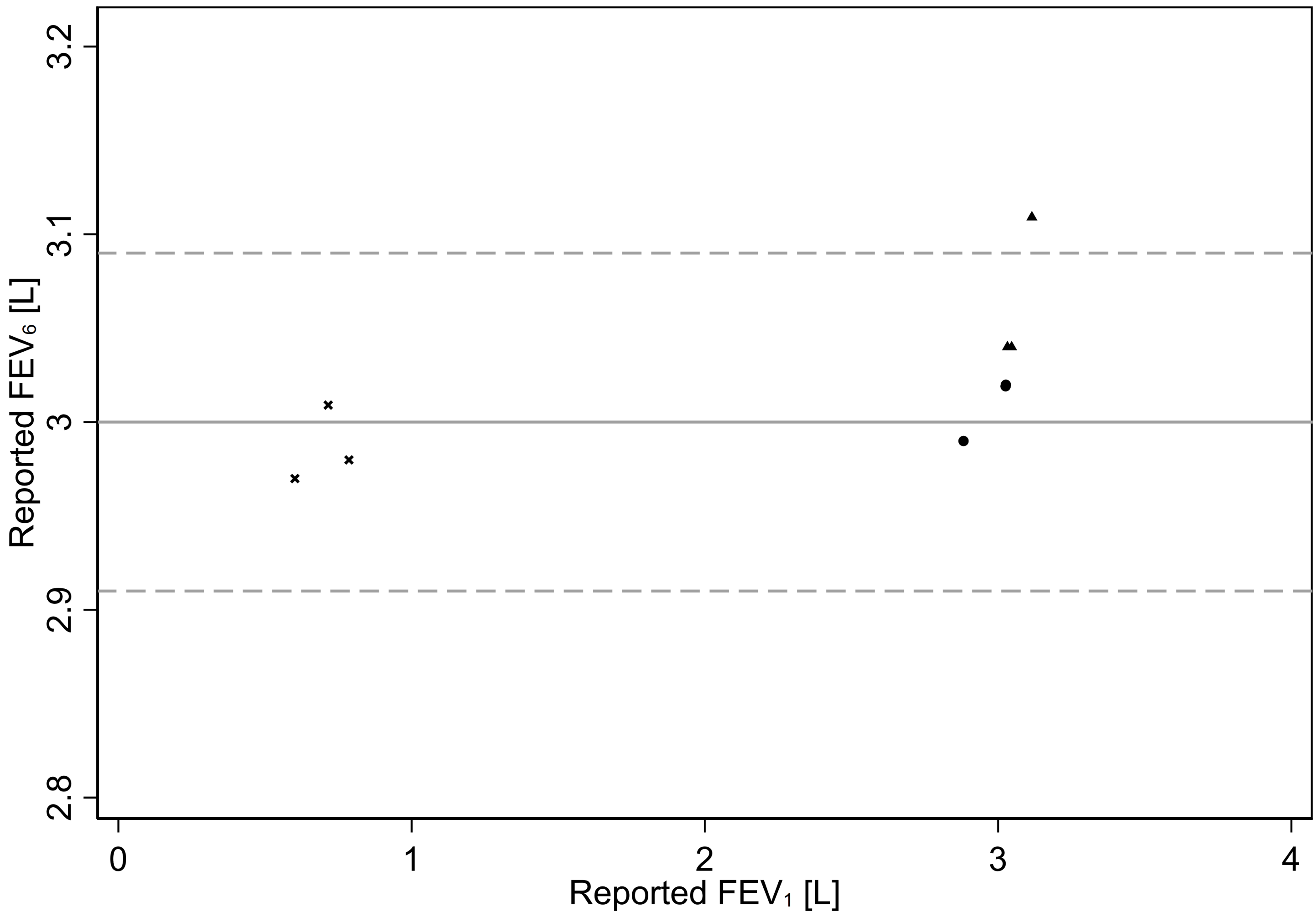

$\varrho_{Spearman}$ = 0.90, $p_{Spearman}$ = 0.001

Legend: × = slow, ● = medium, ▲ = fast

Gray lines = 3.00 L ± 3%

		Slow	Medium	Fast	Total
Measurements	n	3	3	3	9
Reported FEV_1	Geometric mean [95% CI]	0.70 [0.61; 0.80]	2.98 [2.89; 3.06]	3.06 [3.02; 3.11]	1.85 [1.14; 3.00]
Reported FEV_6	Geometric mean [95% CI]	2.99 [2.96; 3.01]	3.01 [2.99; 3.03]	3.06 [3.02; 3.11]	3.02 [2.99; 3.05]
Measurements with FEV_6 < 2.91 L	n (%)	0 (0%)	0 (0%)	0 (0%)	0 (0%)
Measurements with FEV_6 > 3.09 L	n (%)	0 (0%)	0 (0%)	1 (33%)	1 (11%)

Calibration data for copd-6: Day 9, device 1

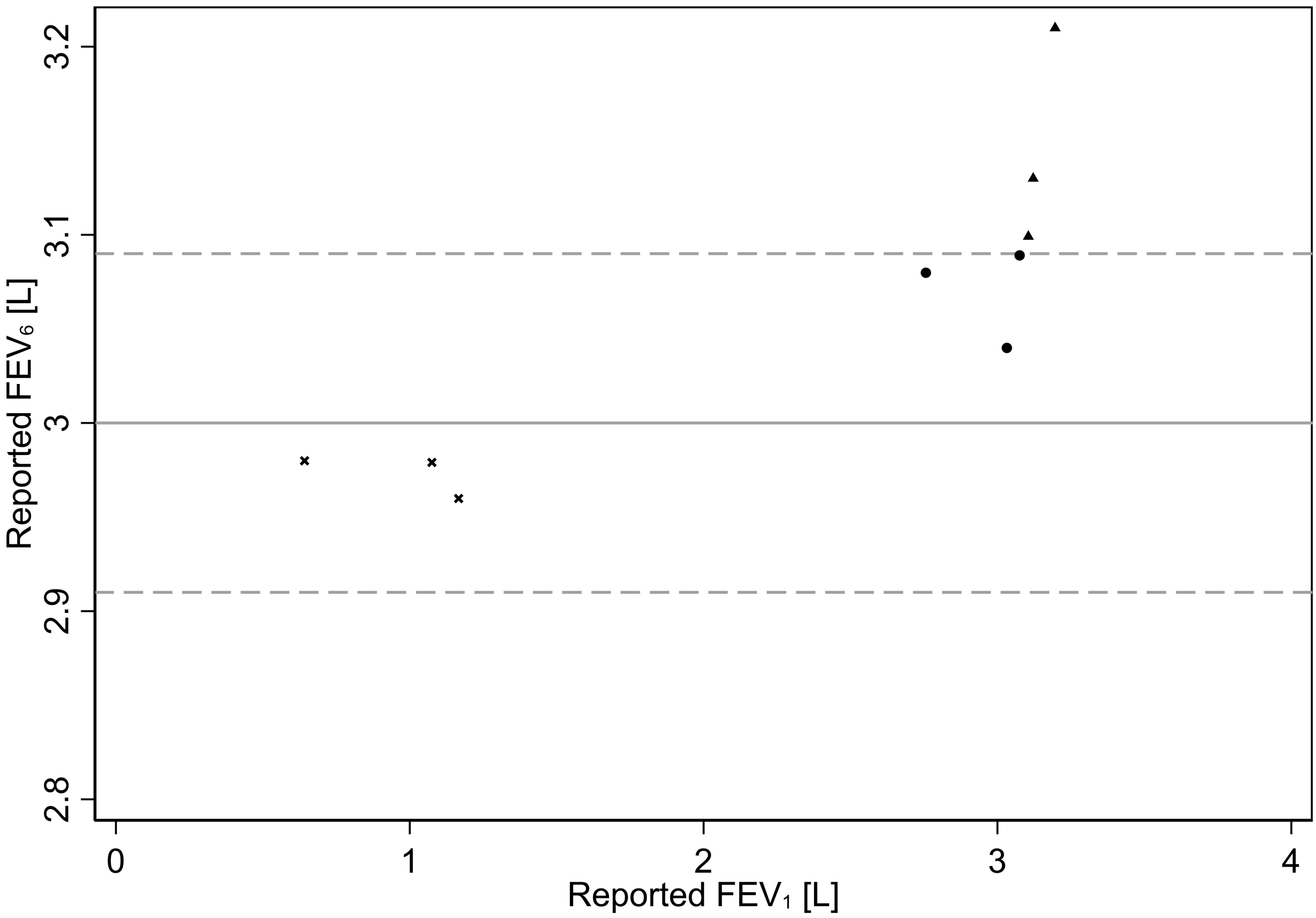

$\varrho_{Spearman} = 0.95$, $p_{Spearman} < 0.001$

Legend: × = slow, ● = medium, ▲ = fast

Gray lines = 3.00 L ± 3%

		Slow	Medium	Fast	Total
Measurements	n	3	3	3	9
Reported FEV_1	Geometric mean [95% CI]	0.93 [0.65; 1.33]	2.95 [2.75; 3.16]	3.14 [3.09; 3.19]	2.05 [1.37; 3.06]
Reported FEV_6	Geometric mean [95% CI]	2.97 [2.96; 2.99]	3.07 [3.04; 3.10]	3.15 [3.08; 3.21]	3.06 [3.01; 3.12]
Measurements with FEV_6 < 2.91 L	n (%)	0 (0%)	0 (0%)	0 (0%)	0 (0%)
Measurements with FEV_6 > 3.09 L	n (%)	0 (0%)	0 (0%)	3 (100%)	3 (33%)

Calibration data for copd-6: Day 9, device 2

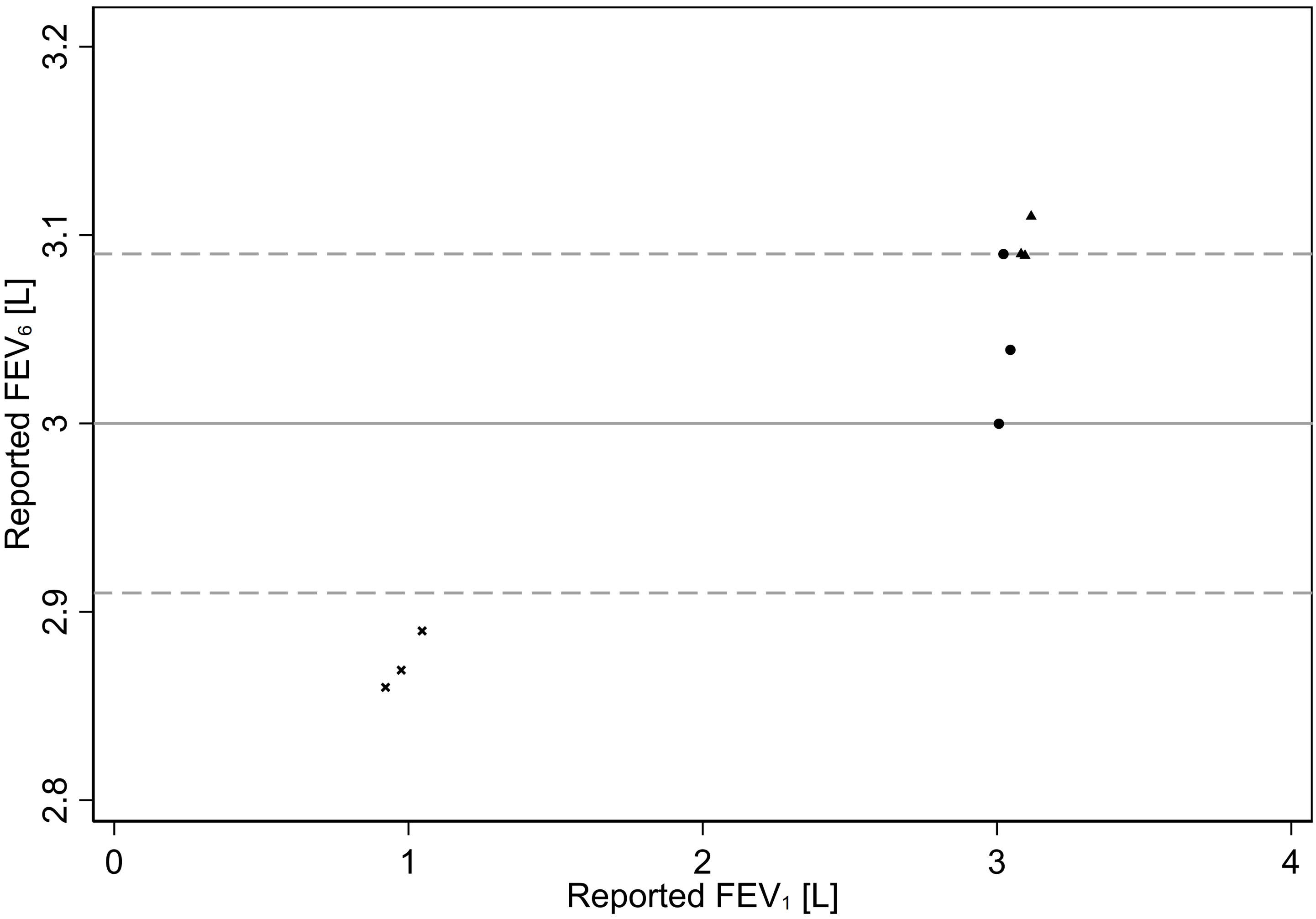

$\varrho_{Spearman} = 0.91$, $p_{Spearman} = 0.001$

Legend: × = slow, ● = medium, ▲ = fast

Gray lines = 3.00 L ± 3%

		Slow	Medium	Fast	Total
Measurements	n	3	3	3	9
Reported FEV_1	Geometric mean [95% CI]	0.98 [0.92; 1.04]	3.02 [3.00; 3.05]	3.10 [3.08; 3.11]	2.09 [1.44; 3.04]
Reported FEV_6	Geometric mean [95% CI]	2.87 [2.86; 2.89]	3.04 [2.99; 3.09]	3.10 [3.08; 3.11]	3.00 [2.94; 3.07]
Measurements with FEV_6 < 2.91 L	n (%)	3 (100%)	0 (0%)	0 (0%)	3 (33%)
Measurements with FEV_6 > 3.09 L	n (%)	0 (0%)	0 (0%)	1 (33%)	1 (11%)

Calibration data for copd-6: Day 10, device 1

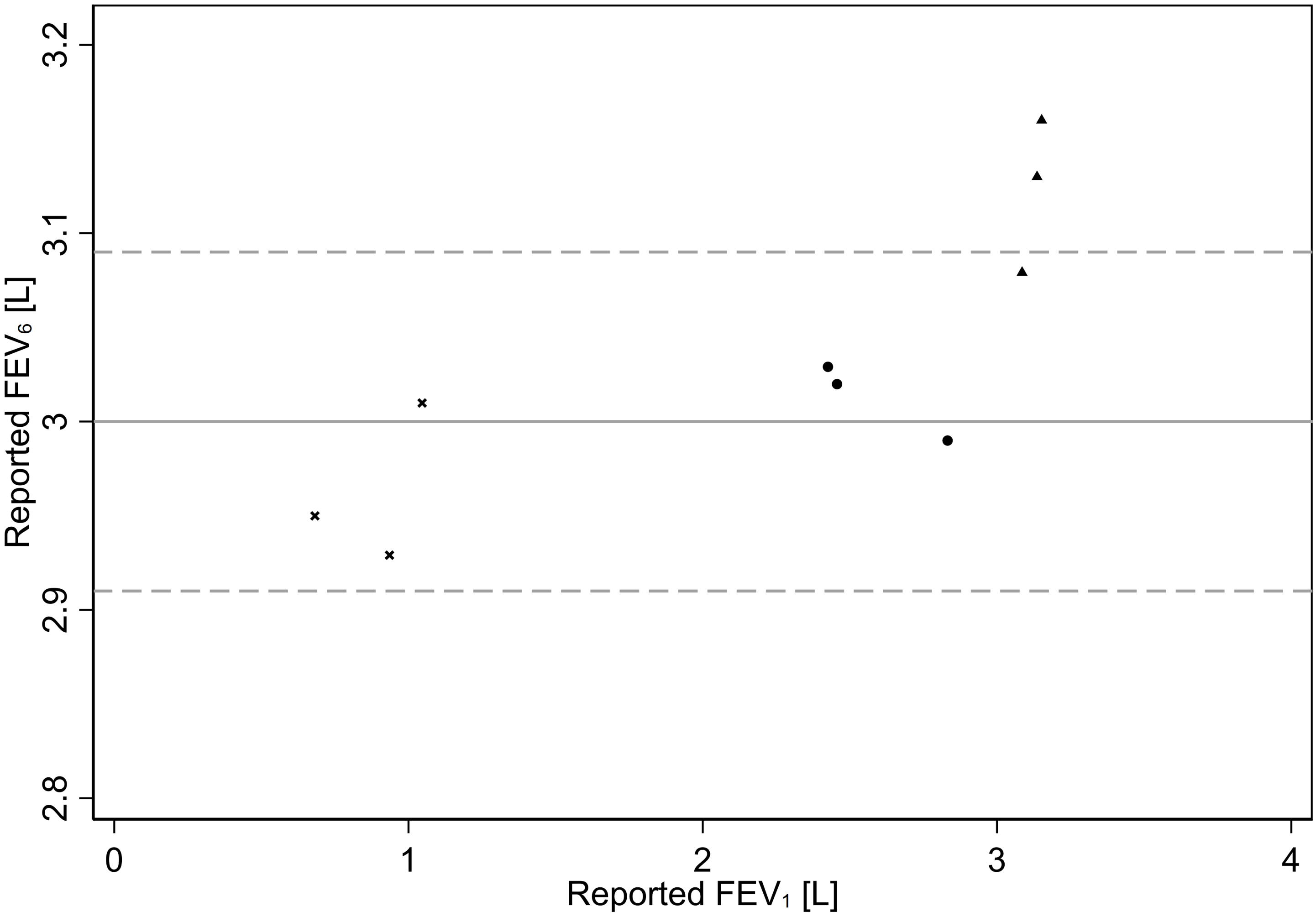

$\varrho_{Spearman} = 0.90$, $p_{Spearman} = 0.001$

Legend: × = slow, ● = medium, ▲ = fast

Gray lines = 3.00 L ± 3%

		Slow	Medium	Fast	Total
Measurements	n	3	3	3	9
Reported FEV_1	Geometric mean [95% CI]	0.87 [0.69; 1.11]	2.56 [2.32; 2.83]	3.12 [3.08; 3.17]	1.91 [1.29; 2.84]
Reported FEV_6	Geometric mean [95% CI]	2.96 [2.92; 3.01]	3.01 [2.99; 3.04]	3.12 [3.08; 3.17]	3.03 [2.98; 3.08]
Measurements with FEV_6 < 2.91 L	n (%)	0 (0%)	0 (0%)	0 (0%)	0 (0%)
Measurements with FEV_6 > 3.09 L	n (%)	0 (0%)	0 (0%)	2 (67%)	2 (22%)

Calibration data for copd-6: Day 10, device 2

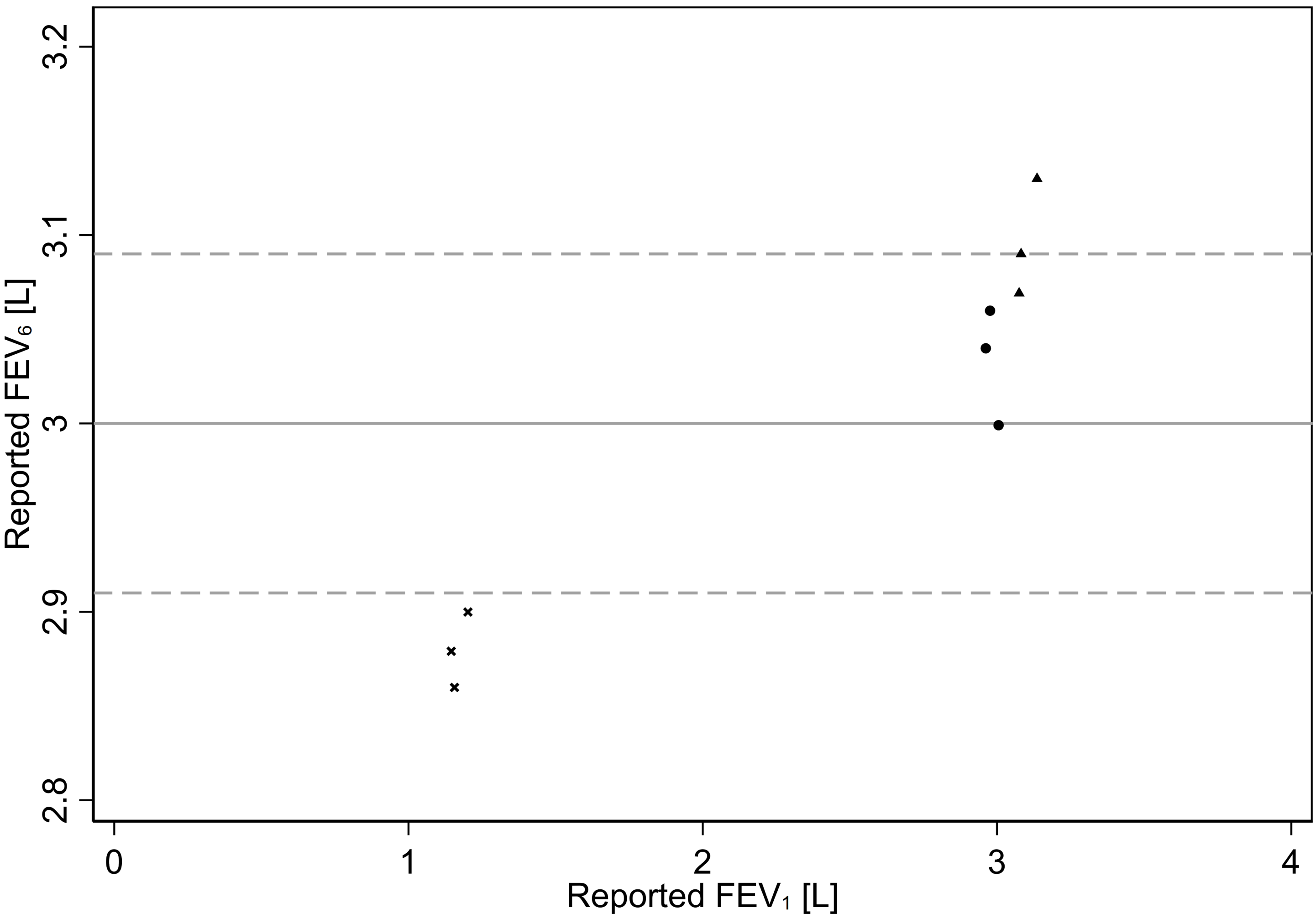

$\varrho_{Spearman} = 0.95$, $p_{Spearman} < 0.001$

Legend: × = slow, ● = medium, ▲ = fast

Gray lines = 3.00 L ± 3%

		Slow	Medium	Fast	Total
Measurements	n	3	3	3	9
Reported FEV_1	Geometric mean [95% CI]	1.17 [1.12; 1.21]	2.98 [2.96; 3.00]	3.10 [3.06; 3.13]	2.21 [1.61; 3.02]
Reported FEV_6	Geometric mean [95% CI]	2.88 [2.86; 2.90]	3.03 [3.00; 3.07]	3.10 [3.06; 3.13]	3.00 [2.94; 3.07]
Measurements with FEV_6 < 2.91 L	n (%)	3 (100%)	0 (0%)	0 (0%)	3 (33%)
Measurements with FEV_6 > 3.09 L	n (%)	0 (0%)	0 (0%)	1 (33%)	1 (11%)

Calibration data for copd-6: Day 11, device 1

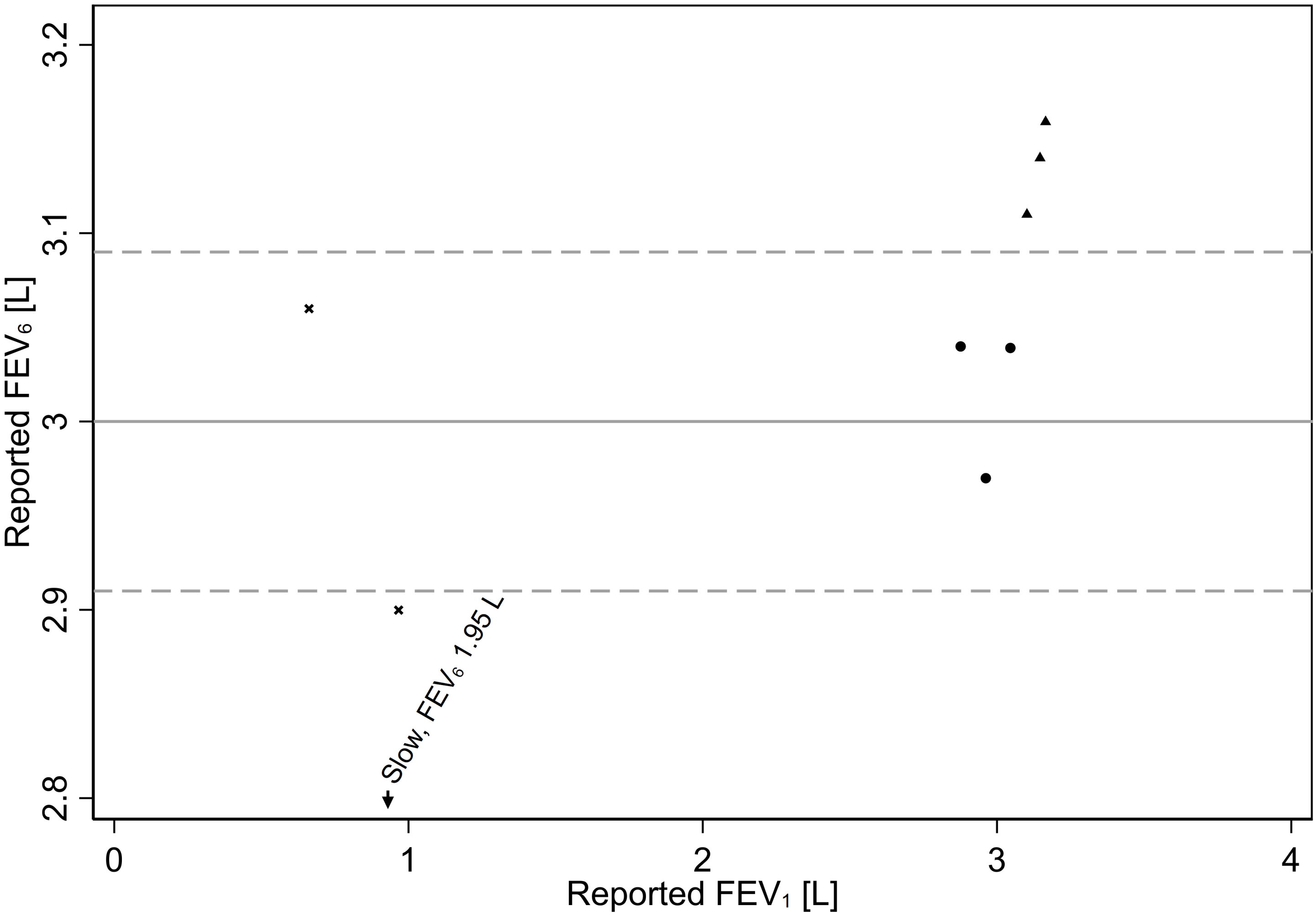

$\varrho_{Spearman} = 0.79$, $p_{Spearman} = 0.011$

Legend: × = slow, ● = medium, ▲ = fast

Gray lines = 3.00 L ± 3%

		Slow	Medium	Fast	Total
Measurements	n	3	3	3	9
Reported FEV_1	Geometric mean [95% CI]	0.84 [0.67; 1.06]	2.96 [2.86; 3.06]	3.14 [3.11; 3.17]	1.98 [1.30; 3.04]
Reported FEV_6	Geometric mean [95% CI]	2.59 [1.96; 3.42]	3.02 [2.97; 3.06]	3.14 [3.11; 3.17]	2.90 [2.63; 3.21]
Measurements with FEV_6 < 2.91 L	n (%)	2 (67%)	0 (0%)	0 (0%)	2 (22%)
Measurements with FEV_6 > 3.09 L	n (%)	0 (0%)	0 (0%)	3 (100%)	3 (33%)

Calibration data for copd-6: Day 11, device 2

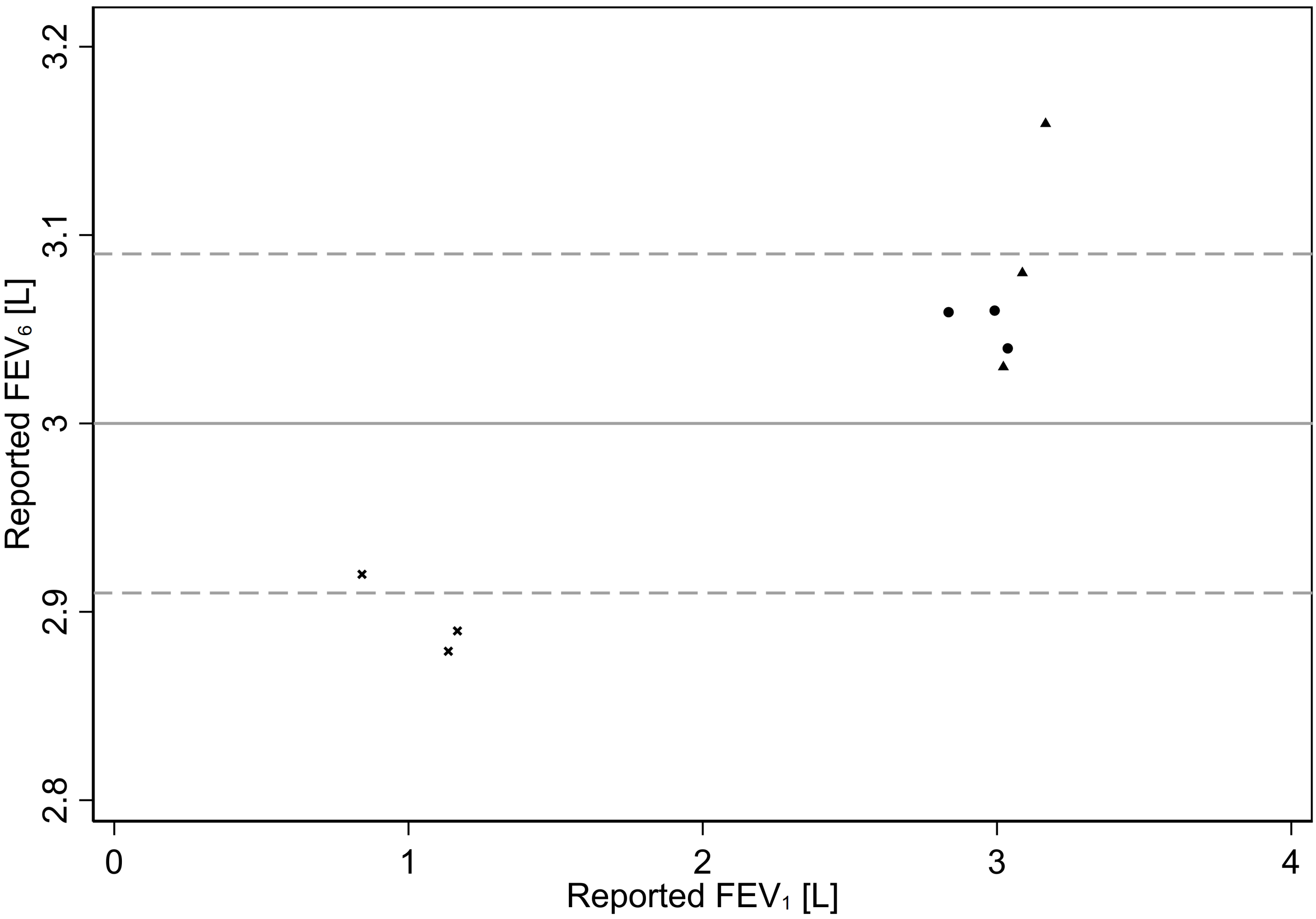

$\varrho_{Spearman} = 0.79$, $p_{Spearman} = 0.011$

Legend: × = slow, ● = medium, ▲ = fast

Gray lines = 3.00 L ± 3%

		Slow	Medium	Fast	Total
Measurements	n	3	3	3	9
Reported FEV_1	Geometric mean [95% CI]	1.04 [0.85; 1.26]	2.95 [2.83; 3.08]	3.09 [3.02; 3.16]	2.11 [1.48; 3.01]
Reported FEV_6	Geometric mean [95% CI]	2.90 [2.87; 2.92]	3.05 [3.04; 3.07]	3.09 [3.02; 3.16]	3.01 [2.95; 3.08]
Measurements with FEV_6 < 2.91 L	n (%)	2 (67%)	0 (0%)	0 (0%)	2 (22%)
Measurements with FEV_6 > 3.09 L	n (%)	0 (0%)	0 (0%)	1 (33%)	1 (11%)

Calibration data for copd-6: Day 12, device 1

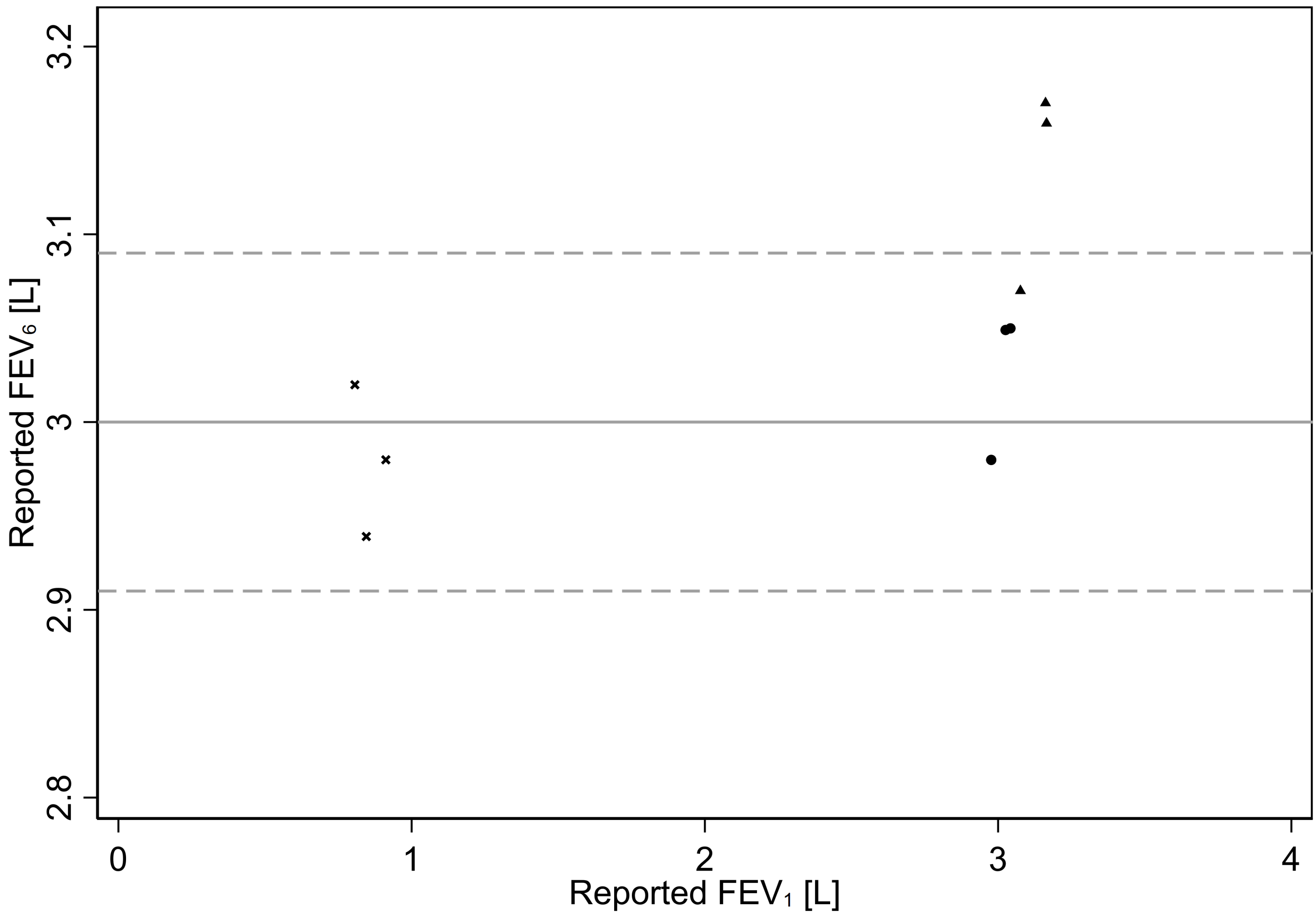

$\varrho_{Spearman} = 0.88$, $p_{Spearman} = 0.002$

Legend: × = slow, ● = medium, ▲ = fast

Gray lines = 3.00 L ± 3%

		**Slow**	**Medium**	**Fast**	**Total**
Measurements	n	3	3	3	9
Reported FEV_1	Geometric mean [95% CI]	0.85 [0.79; 0.92]	3.01 [2.97; 3.06]	3.13 [3.07; 3.20]	2.00 [1.32; 3.05]
Reported FEV_6	Geometric mean [95% CI]	2.98 [2.93; 3.03]	3.03 [2.98; 3.07]	3.13 [3.07; 3.20]	3.05 [3.00; 3.10]
Measurements with FEV_6 < 2.91 L	n (%)	0 (0%)	0 (0%)	0 (0%)	0 (0%)
Measurements with FEV_6 > 3.09 L	n (%)	0 (0%)	0 (0%)	2 (67%)	2 (22%)

Calibration data for copd-6: Day 12, device 2

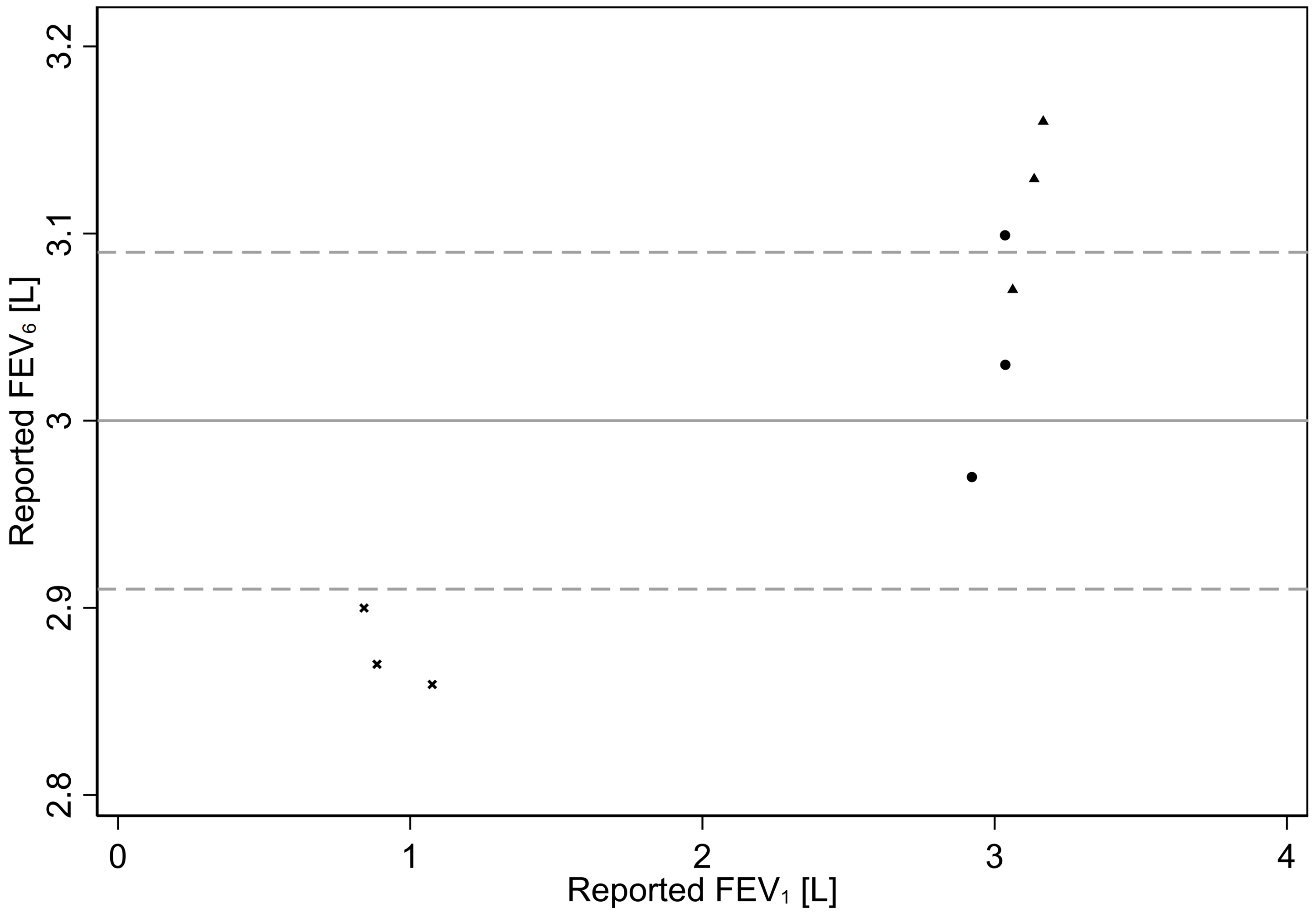

$\varrho_{Spearman} = 0.90$, $p_{Spearman} = 0.001$

Legend: × = slow, ● = medium, ▲ = fast

Gray lines = 3.00 L ± 3%

		Slow	Medium	Fast	Total
Measurements	n	3	3	3	9
Reported FEV_1	Geometric mean [95% CI]	0.93 [0.81; 1.07]	3.00 [2.93; 3.06]	3.12 [3.07; 3.17]	2.06 [1.39; 3.04]
Reported FEV_6	Geometric mean [95% CI]	2.88 [2.85; 2.90]	3.03 [2.96; 3.11]	3.12 [3.07; 3.17]	3.01 [2.93; 3.08]
Measurements with FEV_6 < 2.91 L	n (%)	3 (100%)	0 (0%)	0 (0%)	3 (33%)
Measurements with FEV_6 > 3.09 L	n (%)	0 (0%)	1 (33%)	2 (67%)	3 (33%)

Calibration data for copd-6: Day 13, device 1

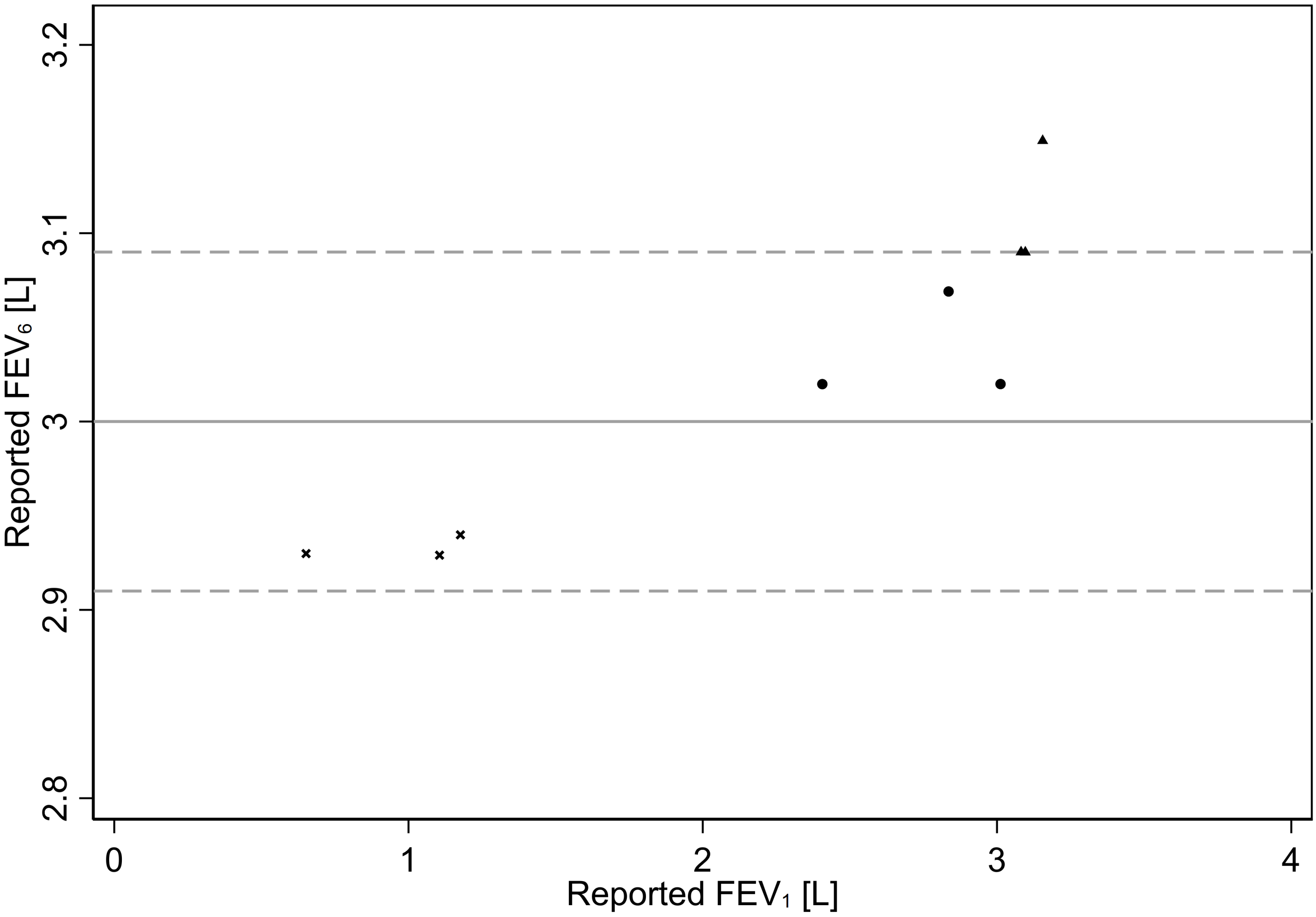

$\varrho_{Spearman} = 0.96$, $p_{Spearman} < 0.001$

Legend: × = slow, ● = medium, ▲ = fast

Gray lines = 3.00 L ± 3%

		Slow	Medium	Fast	Total
Measurements	n	3	3	3	9
Reported FEV_1	Geometric mean [95% CI]	0.95 [0.66; 1.35]	2.74 [2.39; 3.13]	3.11 [3.07; 3.15]	2.01 [1.36; 2.95]
Reported FEV_6	Geometric mean [95% CI]	2.93 [2.93; 2.94]	3.04 [3.00; 3.07]	3.11 [3.07; 3.15]	3.03 [2.97; 3.08]
Measurements with FEV_6 < 2.91 L	n (%)	0 (0%)	0 (0%)	0 (0%)	0 (0%)
Measurements with FEV_6 > 3.09 L	n (%)	0 (0%)	0 (0%)	1 (33%)	1 (11%)

Calibration data for copd-6: Day 13, device 2

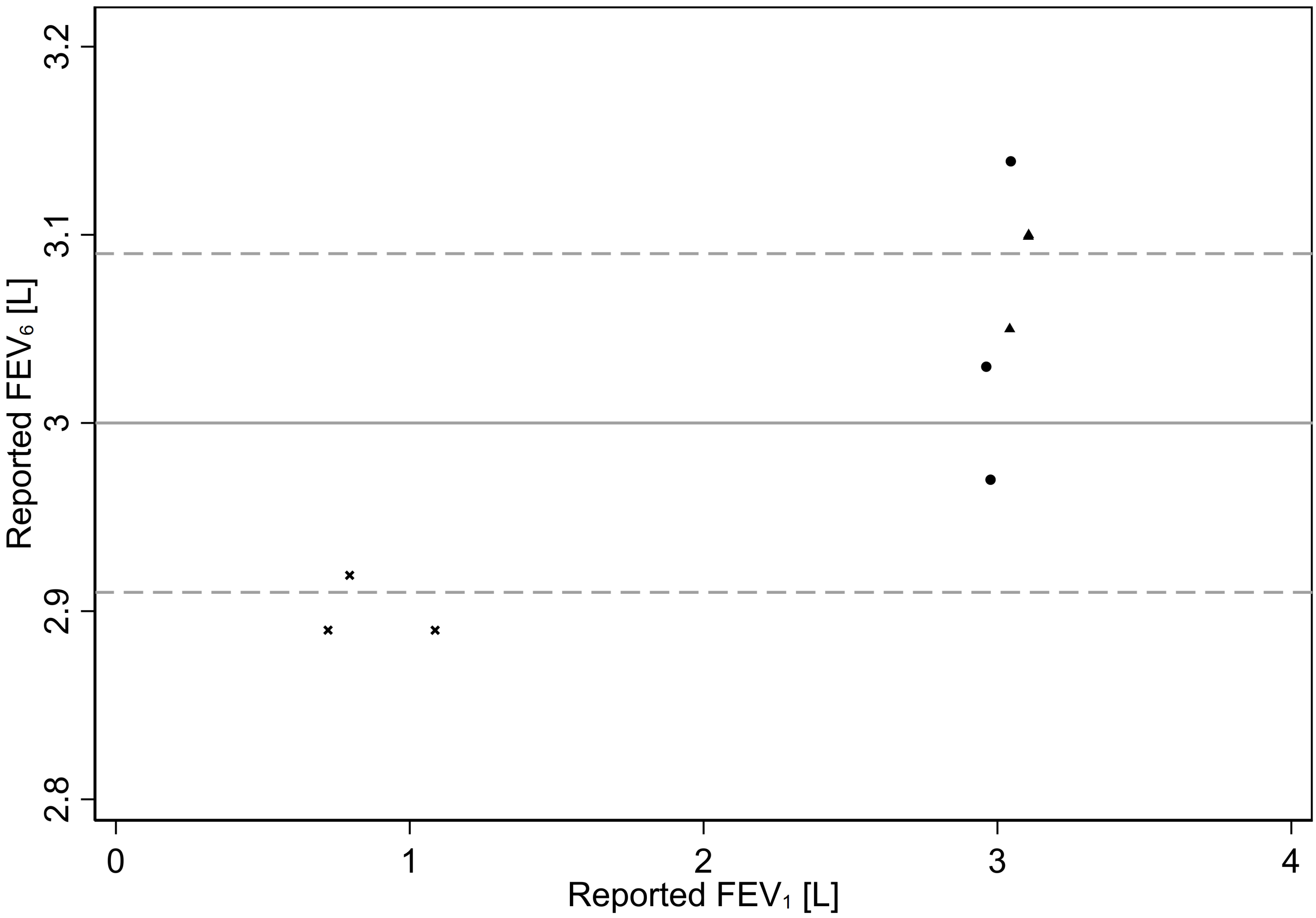

$\varrho_{Spearman} = 0.80$, $p_{Spearman} = 0.010$

Legend: × = slow, ● = medium, ▲ = fast

Gray lines = 3.00 L ± 3%

		Slow	Medium	Fast	Total
Measurements	n	3	3	3	9
Reported FEV_1	Geometric mean [95% CI]	0.85 [0.68; 1.08]	2.99 [2.95; 3.04]	3.08 [3.05; 3.12]	1.99 [1.31; 3.03]
Reported FEV_6	Geometric mean [95% CI]	2.90 [2.88; 2.92]	3.05 [2.95; 3.14]	3.08 [3.05; 3.12]	3.01 [2.95; 3.07]
Measurements with FEV_6 < 2.91 L	n (%)	2 (67%)	0 (0%)	0 (0%)	2 (22%)
Measurements with FEV_6 > 3.09 L	n (%)	0 (0%)	1 (33%)	2 (67%)	3 (33%)

Calibration data for copd-6: Day 14, device 1

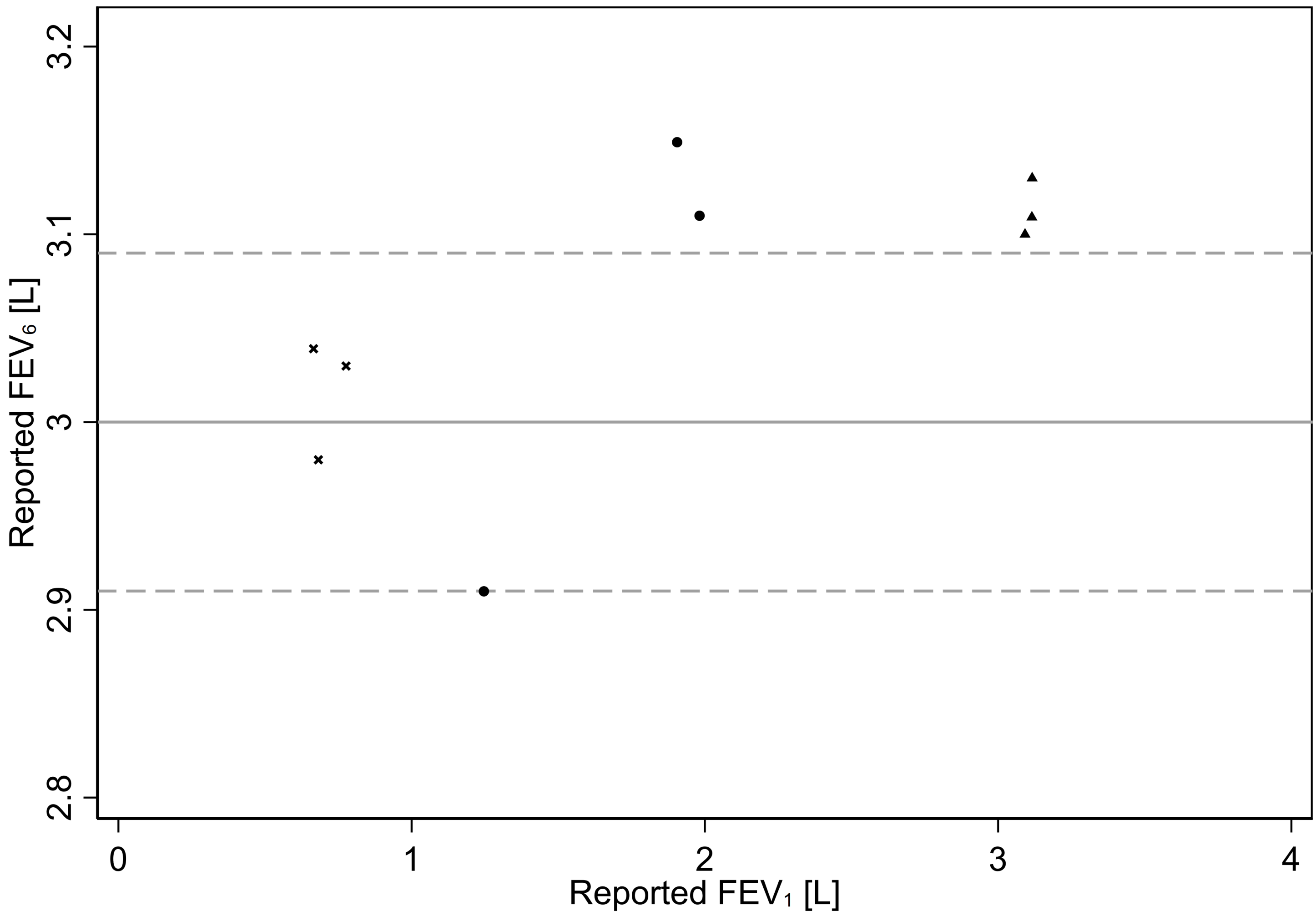

$\varrho_{Spearman}$ = 0.56, $p_{Spearman}$ = 0.120

Legend: × = slow, ● = medium, ▲ = fast

Gray lines = 3.00 L ± 3%

		**Slow**	**Medium**	**Fast**	**Total**
Measurements	n	3	3	3	9
Reported FEV_1	Geometric mean [95% CI]	0.71 [0.64; 0.77]	1.67 [1.25; 2.25]	3.11 [3.10; 3.11]	1.54 [1.00; 2.37]
Reported FEV_6	Geometric mean [95% CI]	3.02 [2.98; 3.05]	3.05 [2.91; 3.21]	3.11 [3.10; 3.13]	3.06 [3.01; 3.11]
Measurements with FEV_6 < 2.91 L	n (%)	0 (0%)	0 (0%)	0 (0%)	0 (0%)
Measurements with FEV_6 > 3.09 L	n (%)	0 (0%)	2 (67%)	3 (100%)	5 (56%)

Calibration data for copd-6: Day 14, device 2

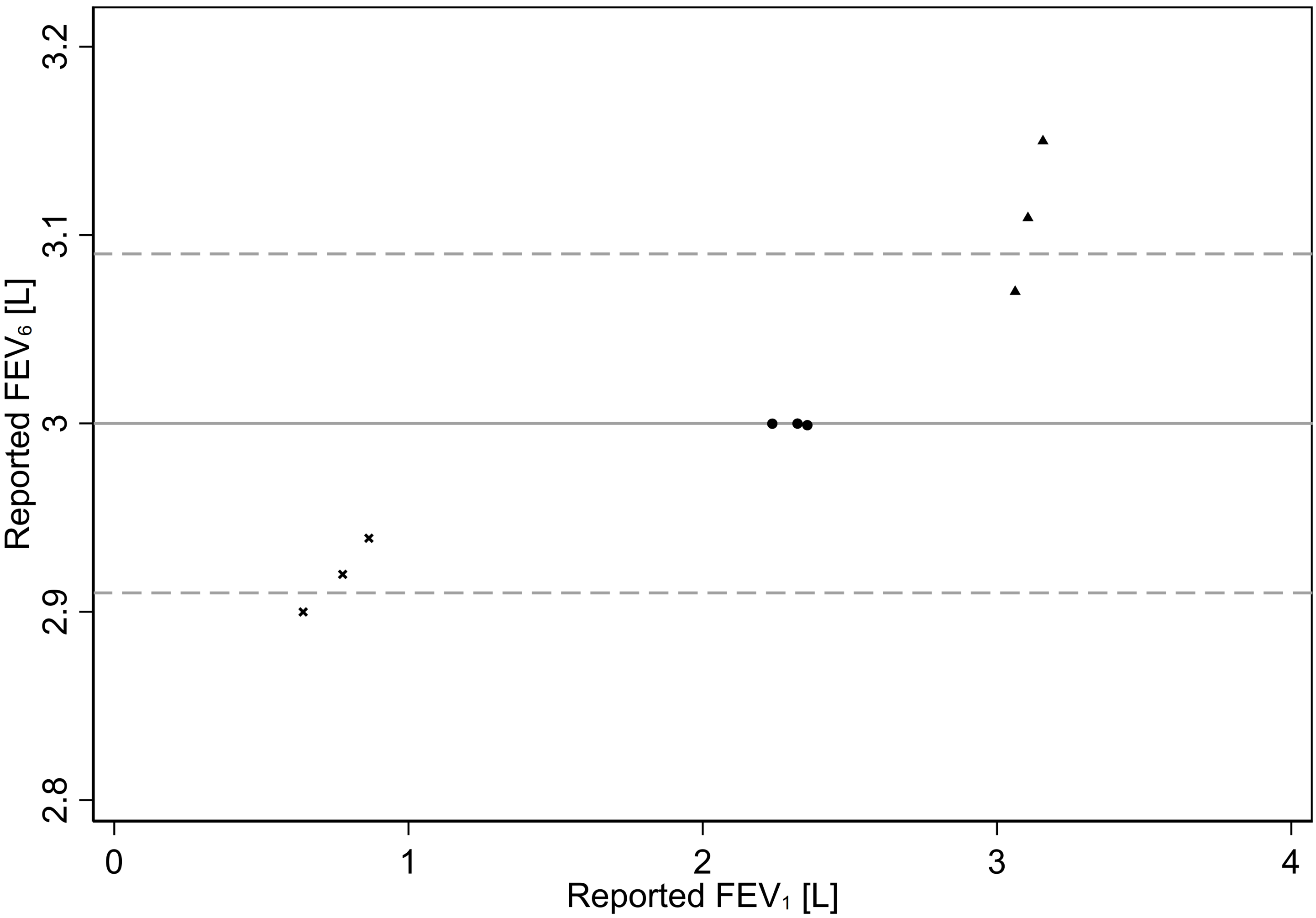

$\varrho_{Spearman} = 0.96$, $p_{Spearman} < 0.001$

Legend: × = slow, ● = medium, ▲ = fast

Gray lines = 3.00 L ± 3%

		Slow	Medium	Fast	Total
Measurements	n	3	3	3	9
Reported FEV_1	Geometric mean [95% CI]	0.76 [0.64; 0.89]	2.30 [2.23; 2.38]	3.11 [3.06; 3.15]	1.75 [1.15; 2.68]
Reported FEV_6	Geometric mean [95% CI]	2.92 [2.90; 2.94]	3.00 [3.00; 3.00]	3.11 [3.06; 3.16]	3.01 [2.95; 3.07]
Measurements with FEV_6 < 2.91 L	n (%)	1 (33%)	0 (0%)	0 (0%)	1 (11%)
Measurements with FEV_6 > 3.09 L	n (%)	0 (0%)	0 (0%)	2 (67%)	2 (22%)

Calibration data for copd-6: Day 15, device 1

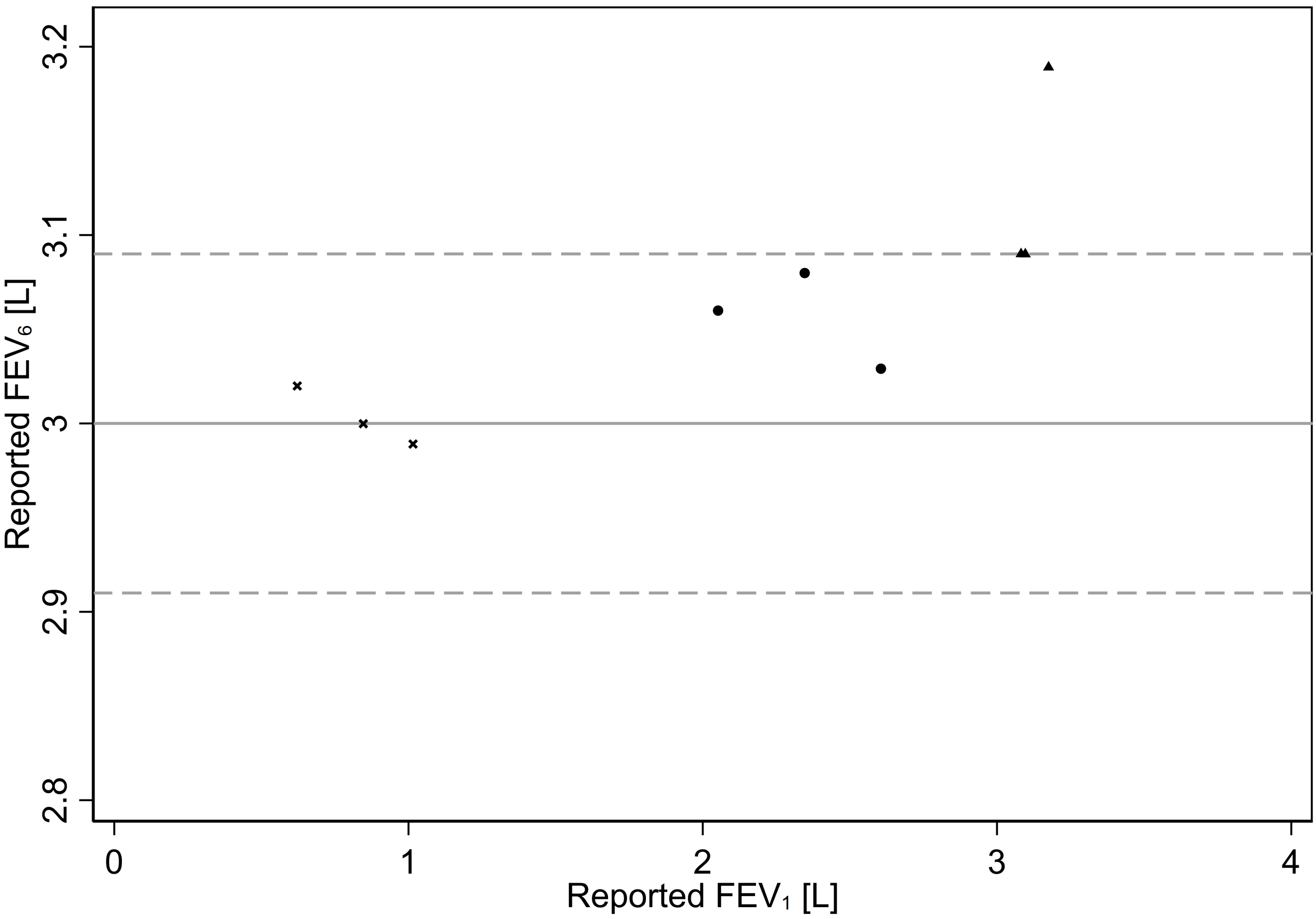

$\varrho_{Spearman} = 0.95$, $p_{Spearman} < 0.001$

Legend: × = slow, ● = medium, ▲ = fast

Gray lines = 3.00 L ± 3%

		Slow	Medium	Fast	Total
Measurements	n	3	3	3	9
Reported FEV_1	Geometric mean [95% CI]	0.81 [0.62; 1.06]	2.32 [2.04; 2.65]	3.12 [3.06; 3.17]	1.80 [1.20; 2.72]
Reported FEV_6	Geometric mean [95% CI]	3.00 [2.99; 3.02]	3.06 [3.03; 3.09]	3.12 [3.06; 3.19]	3.06 [3.02; 3.10]
Measurements with FEV_6 < 2.91 L	n (%)	0 (0%)	0 (0%)	0 (0%)	0 (0%)
Measurements with FEV_6 > 3.09 L	n (%)	0 (0%)	0 (0%)	1 (33%)	1 (11%)

Calibration data for copd-6: Day 15, device 2

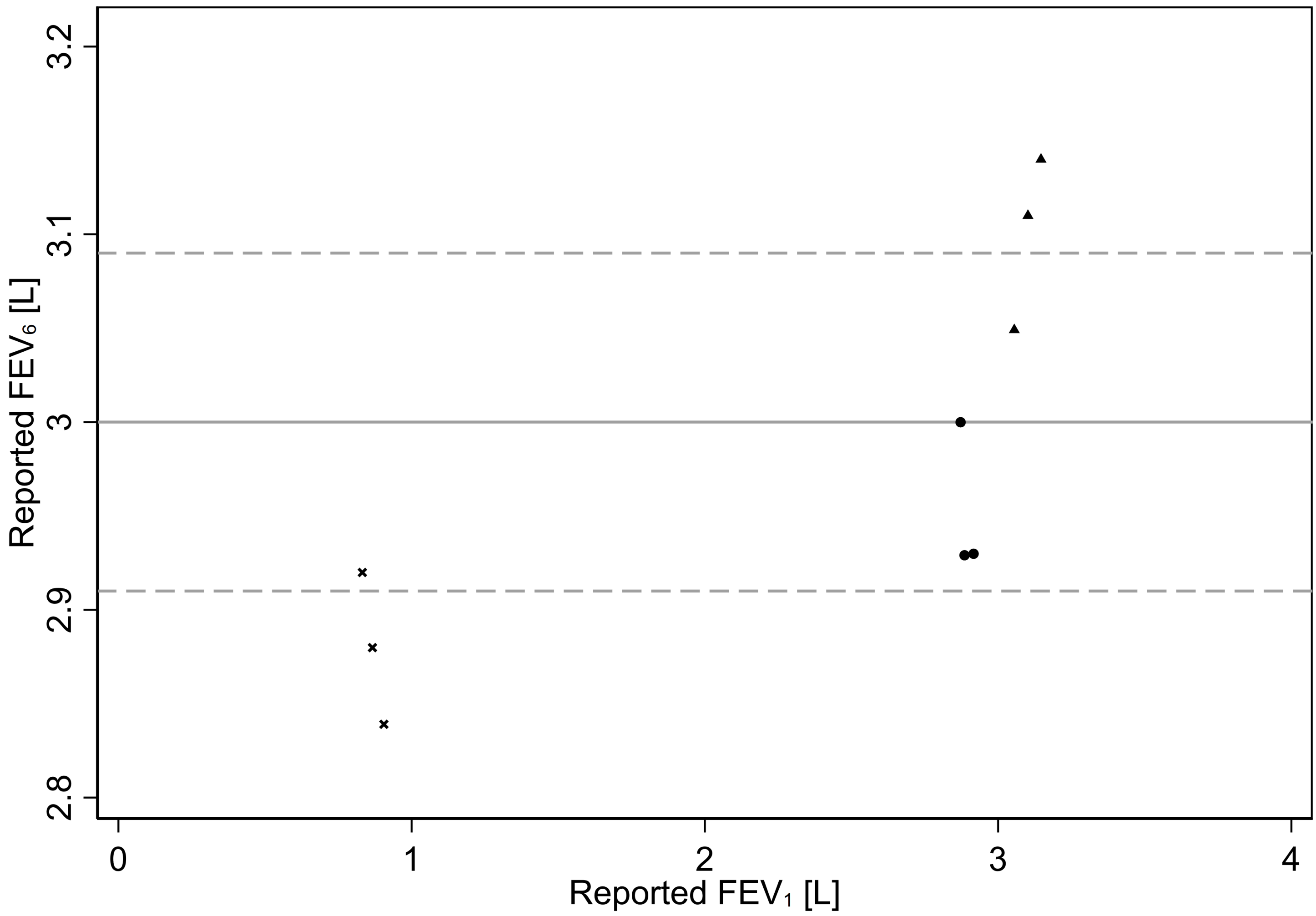

$\varrho_{Spearman} = 0.95$, $p_{Spearman} < 0.001$

Legend: × = slow, ● = medium, ▲ = fast

Gray lines = 3.00 L ± 3%

		Slow	Medium	Fast	Total
Measurements	n	3	3	3	9
Reported FEV_1	Geometric mean [95% CI]	0.87 [0.83; 0.90]	2.89 [2.87; 2.91]	3.10 [3.05; 3.15]	1.98 [1.32; 2.97]
Reported FEV_6	Geometric mean [95% CI]	2.88 [2.83; 2.93]	2.95 [2.91; 3.00]	3.10 [3.05; 3.15]	2.98 [2.91; 3.04]
Measurements with FEV_6 < 2.91 L	n (%)	2 (67%)	0 (0%)	0 (0%)	2 (22%)
Measurements with FEV_6 > 3.09 L	n (%)	0 (0%)	0 (0%)	2 (67%)	2 (22%)

Calibration data for copd-6: Day 16, device 2

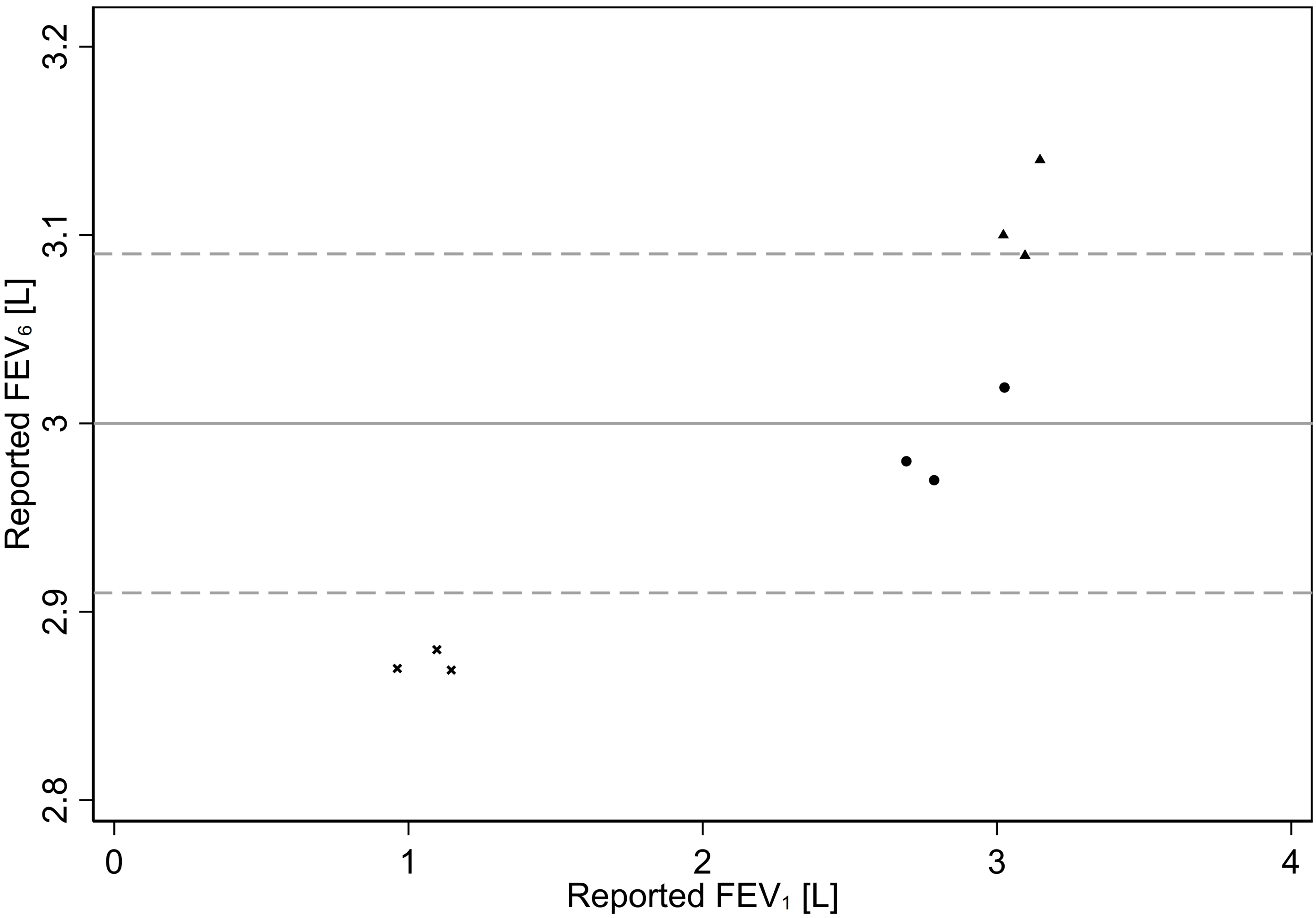

$\varrho_{Spearman} = 0.95$, $p_{Spearman} < 0.001$

Legend: × = slow, ● = medium, ▲ = fast

Gray lines = 3.00 L ± 3%

		Slow	Medium	Fast	Total
Measurements	n	3	3	3	9
Reported FEV_1	Geometric mean [95% CI]	1.06 [0.97; 1.17]	2.83 [2.65; 3.02]	3.09 [3.02; 3.15]	2.10 [1.50; 2.94]
Reported FEV_6	Geometric mean [95% CI]	2.87 [2.87; 2.88]	2.99 [2.96; 3.02]	3.11 [3.08; 3.14]	2.99 [2.92; 3.06]
Measurements with $FEV_6 < 2.91$ L	n (%)	3 (100%)	0 (0%)	0 (0%)	3 (33%)
Measurements with $FEV_6 > 3.09$ L	n (%)	0 (0%)	0 (0%)	2 (67%)	2 (22%)

Calibration data for copd-6: Day 17, device 1

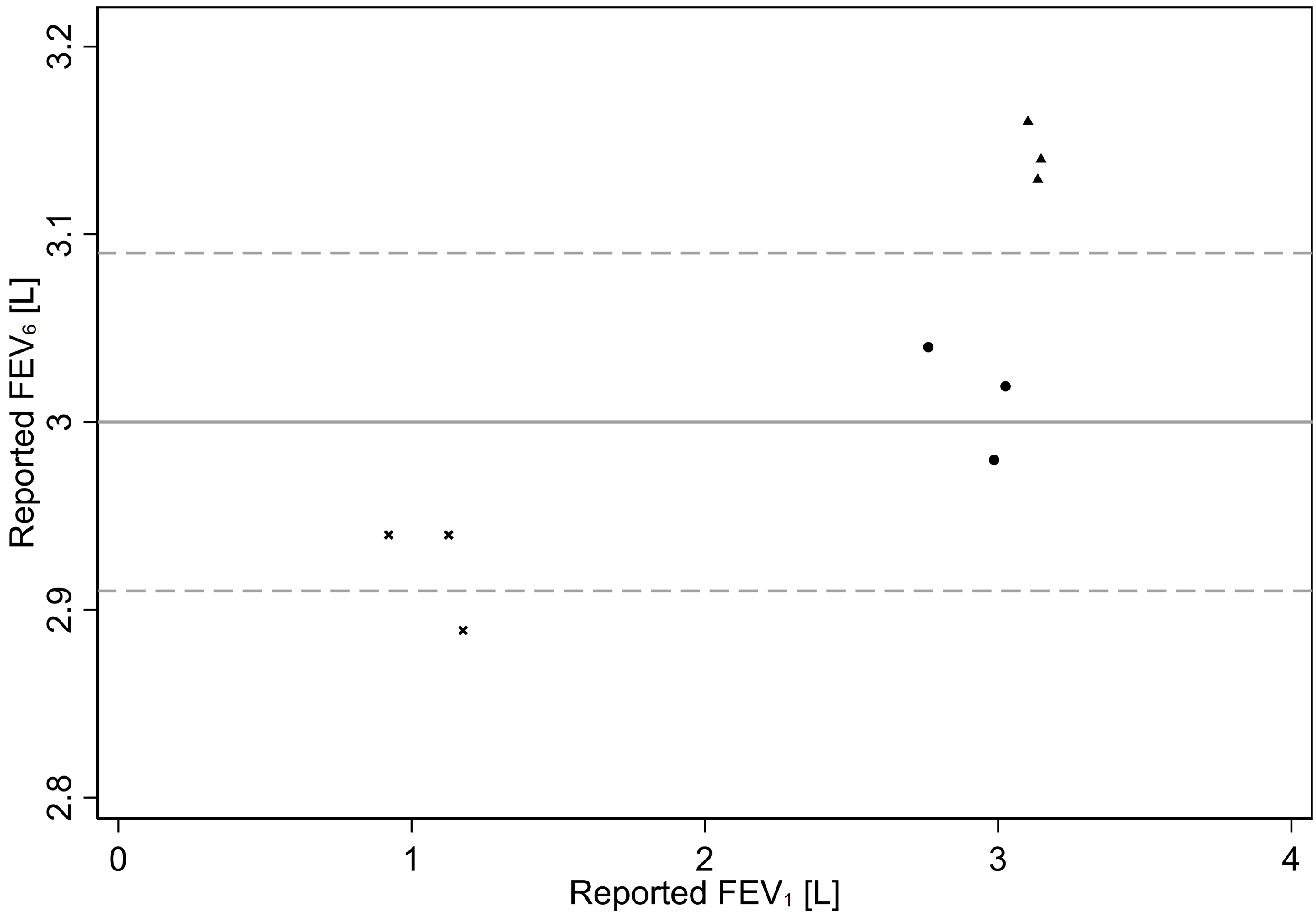

$\varrho_{Spearman} = 0.95$, $p_{Spearman} < 0.001$

Legend: × = slow, ● = medium, ▲ = fast

Gray lines = 3.00 L ± 3%

		Slow	Medium	Fast	Total
Measurements	n	3	3	3	9
Reported FEV_1	Geometric mean [95% CI]	1.07 [0.93; 1.23]	2.92 [2.77; 3.08]	3.13 [3.11; 3.14]	2.14 [1.52; 3.01]
Reported FEV_6	Geometric mean [95% CI]	2.92 [2.89; 2.96]	3.01 [2.98; 3.05]	3.14 [3.13; 3.16]	3.03 [2.96; 3.09]
Measurements with FEV_6 < 2.91 L	n (%)	1 (33%)	0 (0%)	0 (0%)	1 (11%)
Measurements with FEV_6 > 3.09 L	n (%)	0 (0%)	0 (0%)	3 (100%)	3 (33%)

Calibration data for copd-6: Day 17, device 2

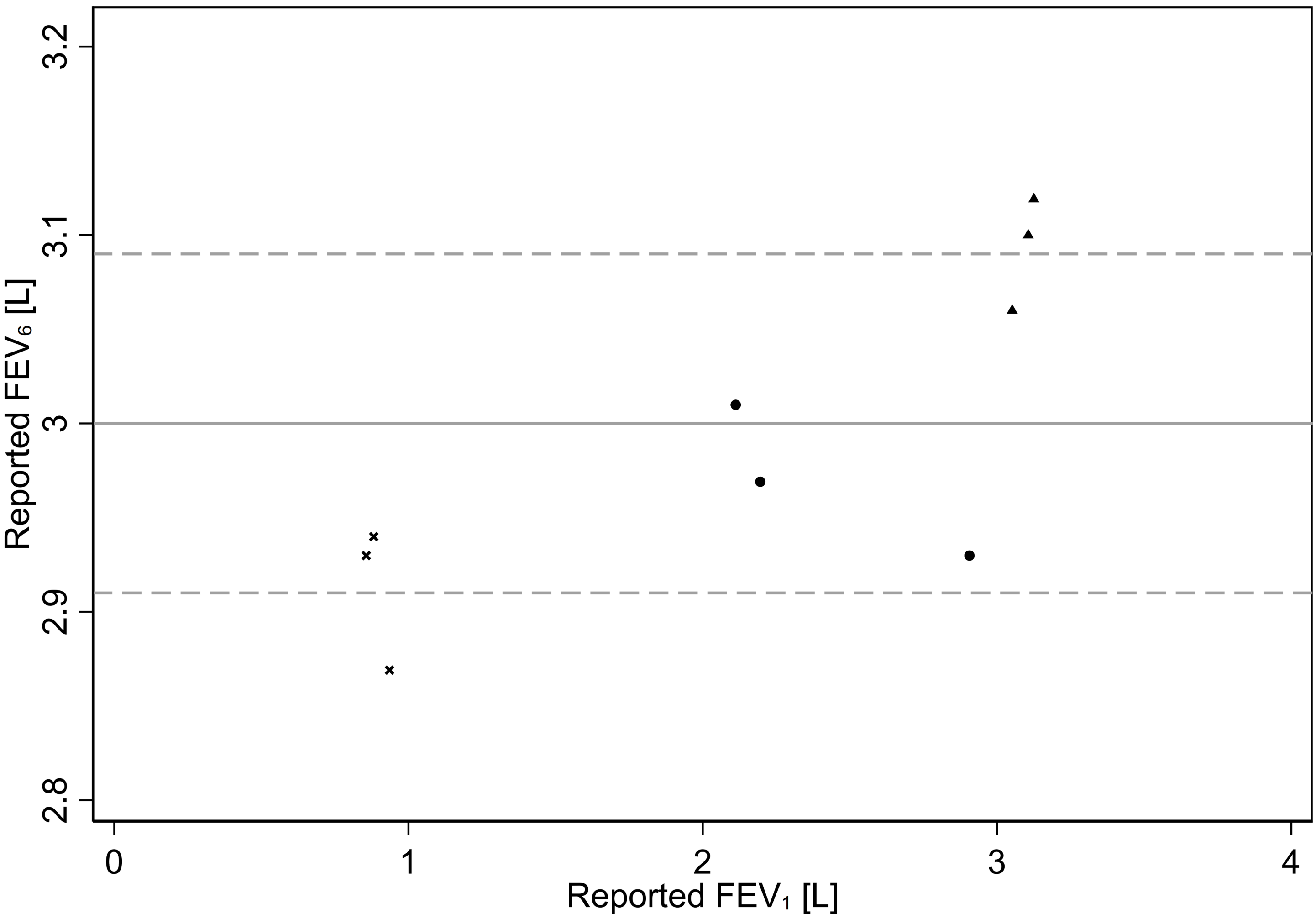

$\varrho_{Spearman}$ = 0.87, $p_{Spearman}$ = 0.002

Legend: × = slow, ● = medium, ▲ = fast

Gray lines = 3.00 L ± 3%

		Slow	Medium	Fast	Total
Measurements	n	3	3	3	9
Reported FEV_1	Geometric mean [95% CI]	0.89 [0.85; 0.94]	2.38 [1.96; 2.89]	3.09 [3.06; 3.13]	1.87 [1.28; 2.73]
Reported FEV_6	Geometric mean [95% CI]	2.91 [2.87; 2.96]	2.97 [2.92; 3.02]	3.09 [3.06; 3.13]	2.99 [2.94; 3.05]
Measurements with FEV_6 < 2.91 L	n (%)	1 (33%)	0 (0%)	0 (0%)	1 (11%)
Measurements with FEV_6 > 3.09 L	n (%)	0 (0%)	0 (0%)	2 (67%)	2 (22%)

Calibration data for copd-6: Day 18, device 1

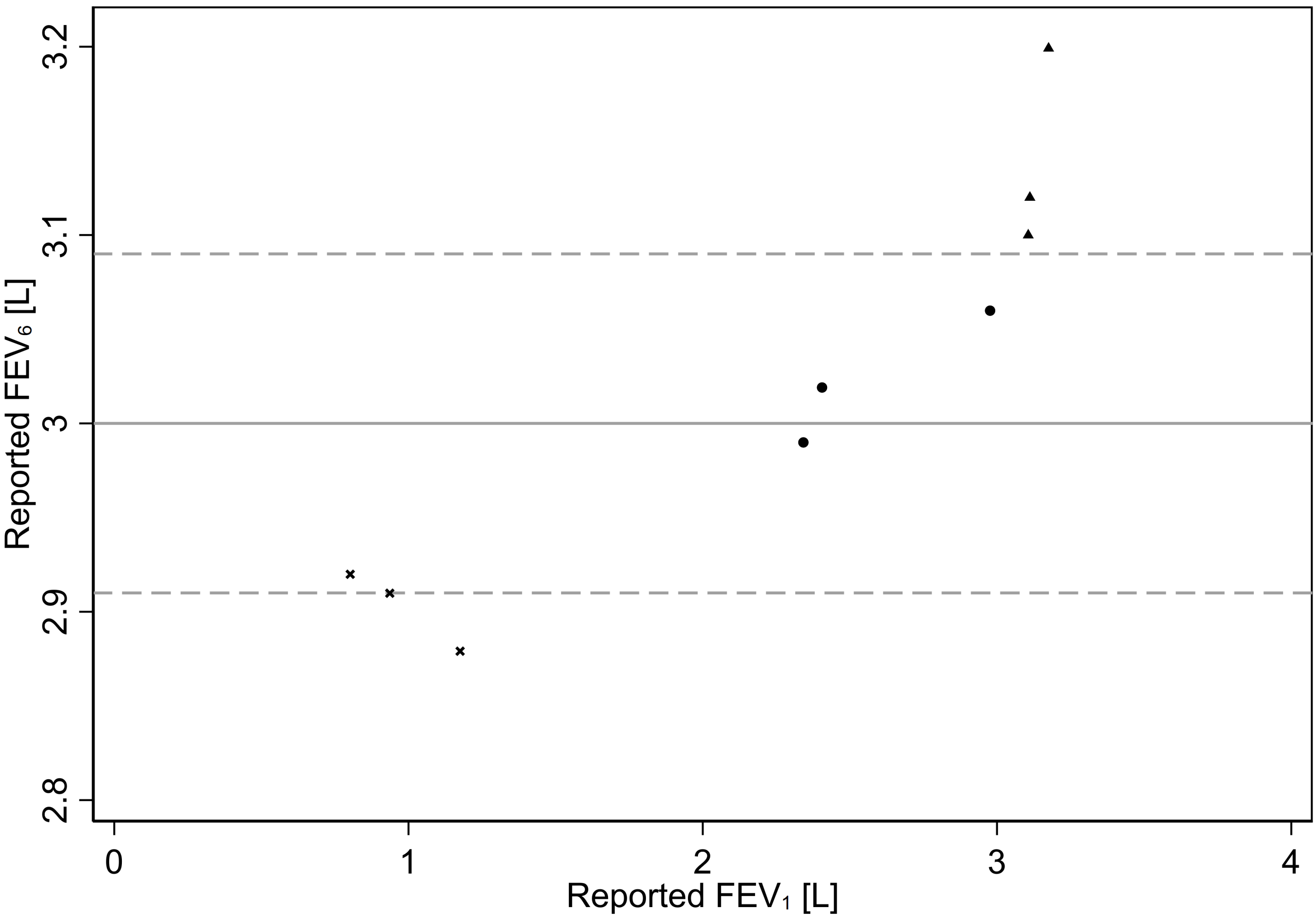

$\varrho_{Spearman} = 0.95$, $p_{Spearman} < 0.001$

Legend: × = slow, ● = medium, ▲ = fast

Gray lines = 3.00 L ± 3%

		Slow	Medium	Fast	Total
Measurements	n	3	3	3	9
Reported FEV_1	Geometric mean [95% CI]	0.96 [0.78; 1.18]	2.56 [2.21; 2.96]	3.13 [3.09; 3.17]	1.97 [1.37; 2.84]
Reported FEV_6	Geometric mean [95% CI]	2.90 [2.88; 2.93]	3.02 [2.98; 3.06]	3.14 [3.08; 3.20]	3.02 [2.95; 3.09]
Measurements with FEV_6 < 2.91 L	n (%)	1 (33%)	0 (0%)	0 (0%)	1 (11%)
Measurements with FEV_6 > 3.09 L	n (%)	0 (0%)	0 (0%)	3 (100%)	3 (33%)

Calibration data for copd-6: Day 18, device 2

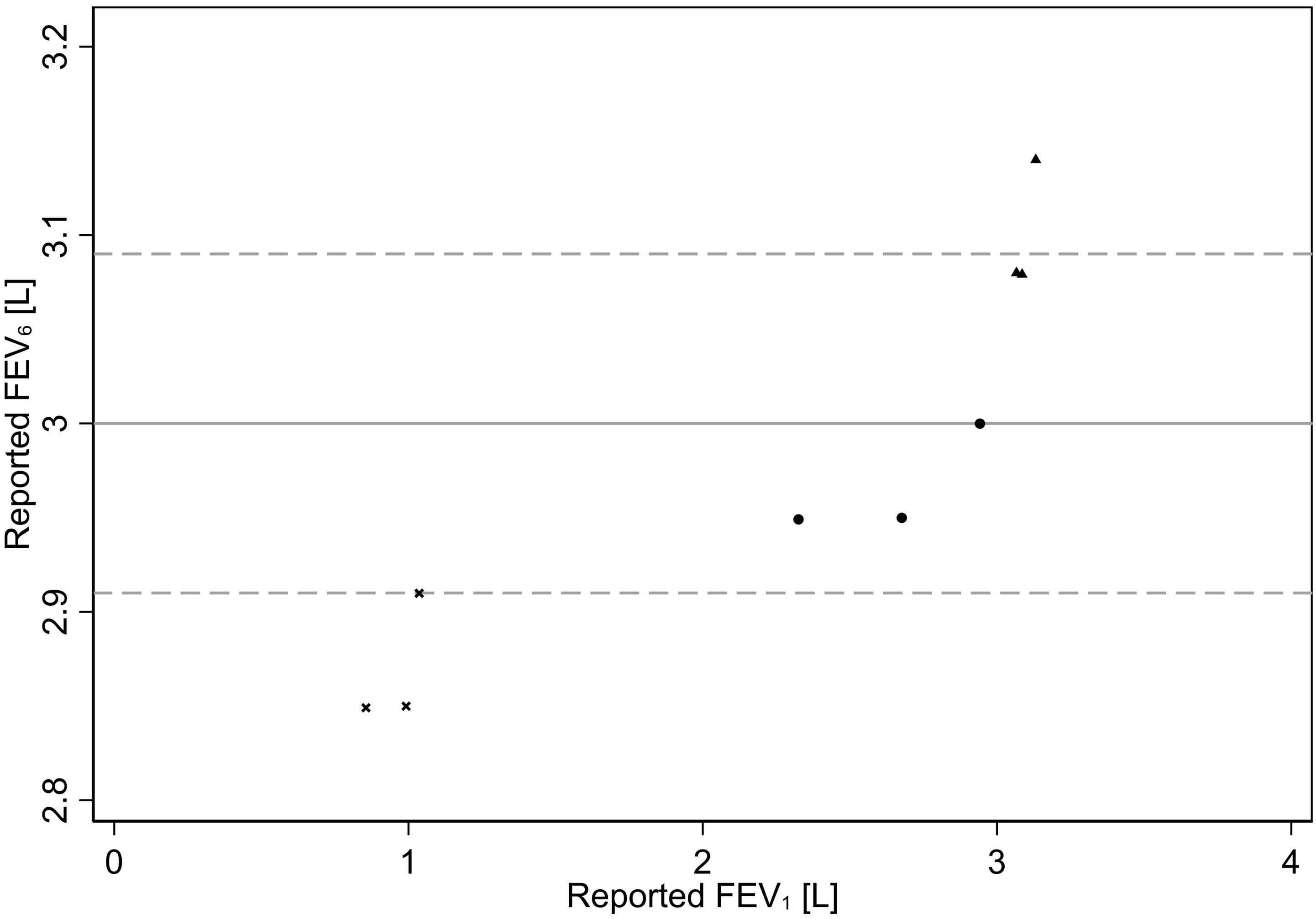

$\varrho_{Spearman} = 0.96$, $p_{Spearman} < 0.001$

Legend: × = slow, ● = medium, ▲ = fast

Gray lines = 3.00 L ± 3%

		Slow	Medium	Fast	Total
Measurements	n	3	3	3	9
Reported FEV_1	Geometric mean [95% CI]	0.96 [0.85; 1.08]	2.63 [2.30; 3.02]	3.09 [3.05; 3.14]	1.98 [1.38; 2.85]
Reported FEV_6	Geometric mean [95% CI]	2.87 [2.83; 2.91]	2.97 [2.93; 3.00]	3.10 [3.06; 3.14]	2.98 [2.91; 3.05]
Measurements with FEV_6 < 2.91 L	n (%)	2 (67%)	0 (0%)	0 (0%)	2 (22%)
Measurements with FEV_6 > 3.09 L	n (%)	0 (0%)	0 (0%)	1 (33%)	1 (11%)
