# Supplementary material for: Precision and accuracy of FEV1 measurements from the Vitalograph copd-6 mini-spirometer in a healthy Ugandan population
Source: PLoS One. 2021 Jun 28;16(6):e0253319. doi: 10.1371/journal.pone.0253319 (PMC8238209; doi:10.1371/journal.pone.0253319)
Supplement: Attachment — Submitted filename: PLOS_ONE_copd-6_rebuttal_letter_20201125.docx [file pone.0253319.s006.docx]

Rebuttal letter

Manuscript ID: PONE-D-20-31069

Title: “The Vitalograph copd-6 mini-spirometer as more than a screening device: Validation of FEV_1_ in a healthy Ugandan population”

# Journal requirements

**Comment**

*1. Please ensure that your manuscript meets PLOS ONE's style requirements, including those for file naming. The PLOS ONE style templates can be found at*

[*https://journals.plos.org/plosone/s/file?id=wjVg/PLOSOne_formatting_sample_main_body.pdf*](https://journals.plos.org/plosone/s/file?id=wjVg/PLOSOne_formatting_sample_main_body.pdf) *and*

[*https://journals.plos.org/plosone/s/file?id=ba62/PLOSOne_formatting_sample_title_authors_affiliations.pdf*](https://journals.plos.org/plosone/s/file?id=ba62/PLOSOne_formatting_sample_title_authors_affiliations.pdf)

**Response**

We confirm that the manuscript meets the style requirements, including those for file naming.

**Comment**

*2. In your Methods section, please provide additional information about the participant recruitment method and the demographic details of your participants. Please ensure you have provided sufficient details to replicate the analyses such as: a) the recruitment date range (month and year),*

**Response**

More details on recruitment methods and the demographic details are now added to the section “Methods” 🡺 “Study population”. All data presented in the current paper are from the baseline examinations of the PEXADU study that were conducted in September-October 2018 (see <https://doi.org/10.1136/oemed-2020-106439>).

**Comment**

*b) a description of any inclusion/exclusion criteria that were applied to participant recruitment,*

**Response**

We have now added this information in the section “Methods” 🡺 “Study population”. We recruited participants from a number of farmer’s groups, and invited all members of the visited farmer’s groups, except persons aged < 18 and pregnant women.

**Comment**

*c) a table of relevant demographic details,*

**Response**

Demographic information is provided in Table 1.

**Comment**

*d) a statement as to whether your sample can be considered representative of a larger population,*

**Response**

We have added a paragraph in the “Discussion” section where we discuss any problems with external validity. Study participants were recruited in a non-random manner by inviting members of a number of farmer's groups in one specific district of Uganda, meaning that the study population may not be representative of Ugandan smallholder farmers overall. However, as the goal of the present study was not to assess the prevalence of lung function impairment among Ugandan smallholder farmers in general, but rather to compare FEV_1_ measurements made with the copd-6 and MicroDL spirometers in a specific study population, we do not think that this poses a threat to the validity of our findings.

**Comment**

*e) a description of how participants were recruited, and*

**Response**

A description of the recruitment has been added to the section “Methods” 🡺 “Study population”. The section includes a reference to a previous paper from the same study population (<https://doi.org/10.1136/oemed-2020-106439>), where additional details on inclusion/exclusion are provided.

**Comment**

*f) descriptions of where participants were recruited and where the research took place.*

**Response**

The section “Methods” 🡺 “Study population” describes that the project took place in the Wakiso District of Uganda. As described above, we have now added that participants were recruited by attending meetings held by a number of farmer’s groups belonging to two local farmer’s organizations in the district. As indicated in the section, participants came to a project examination center to participate.

**Comment**

*3. We noted in your submission details that a portion of your manuscript may have been presented or published elsewhere.*

*"Summary statistics for demographic variables and for MicroDL spirometry results have been included in two previous papers that have been uploaded as "Related papers". These two papers focused on health effects of pesticides in the same study population. Since we have not previously published any results for the copd-6 mini-spirometer, and since the demographic information in table 1 is included only to provide context for the main analyses related to the performance of the spirometers, we do not find that this constitutes dual publication."*

*Please clarify whether this [conference proceeding or publication] was peer-reviewed and formally published. If this work was previously peer-reviewed and published, in the cover letter please provide the reason that this work does not constitute dual publication and should be included in the current manuscript.*

**Response**

The previous paper on MicroDL results has not yet been published, but has been accepted for publication in Thorax (<https://thorax.bmj.com/>). Some descriptive statistics for demographic variables have also been included in a peer-reviewed paper published on <https://doi.org/10.1136/oemed-2020-106439> (but that paper did not include any spirometry results). Furthermore, the summary statistics for the MicroDL that are presented in the second column of Table 2 have also been included in the paper submitted to Thorax. If you prefer, we can remove Table 1 and the second column of Table 2 from the paper. However, we find that without these purely descriptive statistics, readers will have trouble putting the findings of the current paper into proper context. Again, we would like to emphasize that none of our results related to the copd-6 have previously been published.

**Comment**

*4. We note that you have indicated that data from this study are available upon request. PLOS only allows data to be available upon request if there are legal or ethical restrictions on sharing data publicly. For information on unacceptable data access restrictions, please see* [*http://journals.plos.org/plosone/s/data-availability#loc-unacceptable-data-access-restrictions*](http://journals.plos.org/plosone/s/data-availability#loc-unacceptable-data-access-restrictions)*.*

*In your revised cover letter, please address the following prompts:*

*a) If there are ethical or legal restrictions on sharing a de-identified data set, please explain them in detail (e.g., data contain potentially identifying or sensitive patient information) and who has imposed them (e.g., an ethics committee). Please also provide contact information for a data access committee, ethics committee, or other institutional body to which data requests may be sent.*

*b) If there are no restrictions, please upload the minimal anonymized data set necessary to replicate your study findings as either Supporting Information files or to a stable, public repository and provide us with the relevant URLs, DOIs, or accession numbers. Please see http://www.bmj.com/content/340/bmj.c181.long for guidelines on how to de-identify and prepare clinical data for publication. For a list of acceptable repositories, please see http://journals.plos.org/plosone/s/data-availability#loc-recommended-repositories.*

*We will update your Data Availability statement on your behalf to reflect the information you provide.*

**Response**

A minimal data set including the microdata for copd-6 and MicroDL results has already been included in the submission as S4 File. As indicated in the manuscript file, information on family relationships and demographics has not been included in the minimal data file, as doing so would pose a threat to participant confidentiality, which would be unethical. Due to the way that participants were recruited (members of two small agricultural organizations in a small, well-defined geographical area), we deem that pseudonymization is insufficient to ensure that participants cannot be re-identified if the microdata on demographics or family relationships are made public.

The full dataset is stored in the archives of Aarhus University, Department of Public Health, and we have updated the contact information in the section “Availability of data and materials” so that requests for data access should now be sent to the department (instead of the corresponding author). This will ensure that the data remains available, even if the corresponding author is no longer affiliated with the department.

# Reviewer: 1

**Comment**

*Considering methods of performing spirometry I have some objections. Investigation team stated 5 efforts on a diagnostic-quality spirometry, and eventually 4 additional blew if participants did not fulfill standard quality criteria, while according to the ATS/ERS guideline it should be at least three, and most 8 maneuvers for acceptable spirometry. On the copd-6 spirometer they measured just three maneuvers, what is an obstacle, because fatigue is expected after too many efforts of forces expiration. Here were 12 blew into devises in some patients, what it much, and put in question quality of results.*

**Response**

We agree that in principle, participant fatigue could have posed a problem for the validity of our findings, due to the number of blows that each participant performed. However, sensitivity analyses stratified by order of testing (copd-6 before or after MicroDL), plus limited to the first three blows with the MicroDL device, gave similar results as the main analysis, indicating that our findings are unlikely to be considerably influenced by patient fatigue. This is discussed in a new paragraph in the “Discussion” section.

**Comment**

*Time frame between two spirometric measurements is not stated on a diagnostic-quality spirometer and on a copd-6 spirometer, what is another question.*

**Response**

We have now added the following sentence in the section “Methods” 🡺 “Spirometry testing”:

“Only a few minutes passed between testing with each of the two devices.”

**Comment**

*Also, due to diurnal variation of bronchial muscle tone, it is necessary to put the information of time during the day when the measurements were performed.*

**Response**

In the section “Methods” 🡺 “Spirometry testing”, we have added the sentence “Tests were conducted between 7 AM and 5 PM.”

We do not think that diurnal variation in dynamic lung volumes poses a threat to our analyses. As described above, only a few minutes passed between the copd-6 test and the MicroDL test for each participant. Any diurnal effect on FEV_1_, FVC, and FEV_6_ should therefore affect the copd-6 and MicroDL devices equally. To keep the manuscript concise, we have chosen not to include discussion of diurnal effects in the paper, but if the editor prefers we will be glad to include it.

**Comment**

*Again, considering cut-off value for non repeatable spirometric result between two best FEV1 and FVC measurements, the chosen value of difference < 0.25 L is too high, due to the ATS/ERS guideline, the cut-off for difference should be < 0.15 L.*

**Response**

We are aware that according to the ATS guidelines, the best and second-best values of FEV_1_ and FVC should be < 0.15 L. However, using this reproducibility criterion would leave only 120 measurements of FEV_1_ to be analyzed. Due to the theoretical risk of selection bias when discarding the majority of the measurements, we decided to use the less strict reproducibility criterion of < 0.25 L for our main analysis, which allowed us to analyze 171 measurements instead. A sensitivity analysis limited to the 120 measurements with stricter repeatability gave similar results as the main analysis. This is discussed in a new paragraph in the “Discussion” section.

**Comment**

*Another unusual results is that in healthy, rather young and not obese population (46.6 years and BMI 23.3), only 47% of the investigated subjects fulfilled quality criteria for both spirometry devices. Does it mean that the personal was not enough motivated for the participants, or why – this fact need further explanation.*

**Response**

We have added a new paragraph in the “Discussion” section where we discuss the high number of copd-6 results that were discarded. Since the same technicians were responsible for testing with the copd-6 and MicroDL devices, this difference is unlikely to be due to problems with coaching, as poor coaching would have influenced both copd-6 and MicroDL results. Furthermore, the corresponding author supervised the spirometry technicians during data collection, and it was his impression that the technicians vigorously coached the participants during each blow. Out of the 125 persons whose copd-6 results were excluded, 98 (78%) were excluded because they had performed less than two blows without warnings. It could be speculated that the copd-6 is perhaps too conservative when assessing the quality of individual blows (and labelling them as “unacceptable” by giving a warning), but since the device does not export spirograms for manual review, we cannot investigate this further.

# Reviewer: 2

**Comment**

*The high drop out in the COPD6 group should be explained in more details and elaborated in the discussion and the conclusion.*

**Response**

As described above, we have added a new paragraph in the “Discussion” section where we discuss the high number of copd-6 results that were discarded.

**Comment**

*The fact that the investigation was done in the population without obstruction should be more accentuated in the conclusion.*

**Response**

We have revised the first sentence of the conclusion so that it now reads as follows:

“Overall, in our relatively healthy study population the copd-6 slightly overestimates FEV_1_ by 0.04 L, but the bias is flow-dependent.”

**Comment**

*106- less than two or <2 not mixed*

**Response**

Wording in line 106 changed to “less than two” as suggested.

# Comments for figures

**Comment**

*While revising your submission, please upload your figure files to the Preflight Analysis and Conversion Engine (PACE) digital diagnostic tool,* [*https://pacev2.apexcovantage.com/*](https://pacev2.apexcovantage.com/)*. PACE helps ensure that figures meet PLOS requirements. To use PACE, you must first register as a user. Registration is free. Then, login and navigate to the UPLOAD tab, where you will find detailed instructions on how to use the tool. If you encounter any issues or have any questions when using PACE, please email PLOS at figures@plos.org. Please note that Supporting Information files do not need this step.*

**Response**

The figures have now been processed using PACE and re-uploaded.
