# Supplementary material for: Precision and accuracy of FEV1 measurements from the Vitalograph copd-6 mini-spirometer in a healthy Ugandan population
Source: PLoS One. 2021 Jun 28;16(6):e0253319. doi: 10.1371/journal.pone.0253319 (PMC8238209; doi:10.1371/journal.pone.0253319)
Supplement: Attachment — Submitted filename: PLOS_ONE_copd-6_response_to_reviewers_20210520.docx [file pone.0253319.s007.docx]

Response to reviewers

Manuscript ID: PONE-D-20-31069R1

Original title: “The Vitalograph copd-6 mini-spirometer as more than a screening device: Validation of FEV_1_ in a healthy Ugandan population”

Revised title: “Precision and accuracy of FEV_1_ measurements from the Vitalograph copd-6 mini-spirometer in a healthy Ugandan population”

# Journal requirements

**Comment**

*Please review your reference list to ensure that it is complete and correct. If you have cited papers that have been retracted, please include the rationale for doing so in the manuscript text, or remove these references and replace them with relevant current references. Any changes to the reference list should be mentioned in the rebuttal letter that accompanies your revised manuscript. If you need to cite a retracted article, indicate the article’s retracted status in the References list and also include a citation and full reference for the retraction notice.*

**Response**

We have reviewed our reference list and confirm that is complete and correct. We have not cited any retracted papers. As previously described, parts of table 1 and 2 have been published before and are reproduced in the current paper with permission from the publisher. Since the previous paper has now been published in final form, we have moved its citation text from “Acknowledgements” to “References”.

# Additional editor comments

**Comment**

*Dear Authors, as COPD is not the theme of your research please do the suggested changes by the second reviewer.*

**Response**

Done as requested (please see below).

# Reviewer: 1

**Comment**

*Every line of comments were considered and addressed appropriately and seriously. They have answered and added discussion on every topic rised. I have no additional comments.*

**Response**

Thank you for our comments that have helped us improve the manuscript.

# Reviewer: 2

**Comment**

*COPD is diagnosed based on FEV1/FVC ratio. Since in this article only FEV1 accuracy was measured and compared, the title and the whole text should be arranged accordingly. So only what is prooved should be stated. There is no place of mentioning the COPD diagnosis in the context of these data. All references to COPD should be omitted from the introduction, discussion and conclusion or explained how they are connected to FEV1 measuring. So, we can see only the results of FEV1 accuracy in the healty population.*

**Response**

We acknowledge that COPD is diagnosed based on FEV_1_/FVC ratio and not FEV_1_ itself. As requested, we have updated the title, the introduction, discussion and conclusions so that the text does not suggest otherwise.

We have kept a brief section on COPD prevalence and diagnosis in the introduction, since we think that it is important background knowledge for the reader. The high burden of disease due to COPD is the reason why our study is relevant: An affordable, precise and accurate instrument for the determination of pulmonary function would have the potential to improve epidemiological research on pulmonary function impairment, especially in resource-constrained settings.
